# Peripheral inflammation preceeding ischemia impairs neuronal survival through mechanisms involving miR‐127 in aged animals

**DOI:** 10.1111/acel.13287

**Published:** 2020-12-28

**Authors:** Sanna Loppi, Paula Korhonen, Maria Bouvy‐Liivrand, Simone Caligola, Tiia A. Turunen, Mikko P. Turunen, Ana Hernandez de Sande, Natalia Kołosowska, Flavia Scoyni, Anna Rosell, Teresa García‐Berrocoso, Sighild Lemarchant, Hiramani Dhungana, Joan Montaner, Jari Koistinaho, Katja M. Kanninen, Minna U. Kaikkonen, Rosalba Giugno, Merja Heinäniemi, Tarja Malm

**Affiliations:** ^1^ A.I. Virtanen Institute for Molecular Sciences University of Eastern Finland Kuopio Finland; ^2^ Department of Immunobiology University of Arizona Tucson Arizona USA; ^3^ School of Medicine University of Eastern Finland Kuopio Finland; ^4^ Department of Computer Science University of Verona Verona Italy; ^5^ Neurovascular Research Laboratory Vall d’Hebron Institute of Research (VHIR) Universitat Autònoma de Barcelona Barcelona Spain; ^6^ Neuroscience Center University of Helsinki Helsinki Finland

**Keywords:** aging, inflammation, microRNA, proteasome, sequencing, stroke

## Abstract

Ischemic stroke, the third leading cause of death in the Western world, affects mainly the elderly and is strongly associated with comorbid conditions such as atherosclerosis or diabetes, which are pathologically characterized by increased inflammation and are known to influence the outcome of stroke. Stroke incidence peaks during influenza seasons, and patients suffering from infections such as pneumonia prior to stroke exhibit a worse stroke outcome. Earlier studies have shown that comorbidities aggravate the outcome of stroke, yet the mediators of this phenomenon remain obscure. Here, we show that acute peripheral inflammation aggravates stroke‐induced neuronal damage and motor deficits specifically in aged mice. This is associated with increased levels of plasma proinflammatory cytokines, rather than with an increase of inflammatory mediators in the affected brain parenchyma. Nascent transcriptomics data with mature microRNA sequencing were used to identify the neuron‐specific miRNome, in order to decipher dysregulated miRNAs in the brains of aged animals with stroke and co‐existing inflammation. We pinpoint a previously uninvestigated miRNA in the brain, miR‐127, that is highly neuronal, to be associated with increased cell death in the aged, LPS‐injected ischemic mice. Target prediction tools indicate that miR‐127 interacts with several basally expressed neuronal genes, and of these we verify miR‐127 binding to *Psmd3*. Finally, we report reduced expression of miR‐127 in human stroke brains. Our results underline the impact of peripheral inflammation on the outcome of stroke in aged subjects and pinpoint molecular targets for restoring endogenous neuronal capacity to combat ischemic stroke.

## Graphical abstract

Peripheral inflammation preceeding ischemic stroke impairs neuronal survival specifically in the elderly. This increased vulnerability is associated with downregulation of the expression of miR‐127, in both animals and humans. Our results reveal novel molecular targets for treating ischemic stroke, and underline the impact of peripheral inflammation on the outcome of stroke in aged subjects.

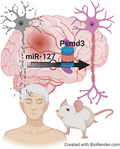

## 1 INTRODUCTION

Stroke is the third leading cause of death in the Western countries (Wang et al., 2016). Despite extensive research, novel neuroprotective treatment paradigms showing promise in preclinical settings have consistently failed in clinical trials, suggesting the inability of preclinical models to mimic human disease. Men seem to be at higher risk of developing stroke until the age of 75 years, but the overall stroke incidence is still higher among women due to their increased life expectancy (Keteepe‐Arachi &Sharma, 2017). This poses a major hurdle in preclinical stroke research: although most of the stroke patients are elderly females with a variable medical background, the majority of the preclinical studies are still carried out using healthy, young males.

The common comorbidities affecting the stroke patients include atherosclerosis, different types of infections, and diabetes, all entailing peripheral inflammation. We together with others have shown that peripheral inflammation preceding stroke aggravates the outcome of brain ischemia where aging is likely to play a significant role (Dénes, 2010; Dhungana et al., 2013). Previous studies have shown that acute lipopolysaccharide (LPS) injection 30 min prior to stroke, modeling a low level of peripheral inflammation present in various comorbid conditions, aggravates ischemia‐induced cell death (McColl et al., 2007) and behavioral impairment (Doll et al., 2015) and is associated with increased IL‐1‐mediated neutrophil infiltration, increased blood–brain barrier (BBB) permeability, and mitochondrial dysfunction (Doll et al., 2015; McColl et al., 2007). However, the impact of aging to resulting motor deficits, or the molecular mechanisms underlying the increased ischemic vulnerability in this context have not been elucidated.

MiRNAs are single‐stranded, non‐coding molecules consisting of 18–25 nucleotides, which control gene expression by either promoting the degradation of messenger‐RNAs (mRNAs) or inhibiting their translation, although they also may have binding sites at the promoter regions of genes (Place et al., 2008). Growing evidence suggests miRNAs as important regulators of cellular survival in ischemic stroke, yet it is completely unknown how miRNA regulation is altered when comorbidities are present.

Here we integrate nascent transcriptomics data (GRO‐seq) with small RNA‐sequencing to identify mature miRNA deregulation among the neuron‐specific miRNome and to evaluate the impact of aging‐associated perturbation on mature miRNA expression. We pinpoint miR‐127‐5p downregulation and association with decreased neuronal survival in a mouse model of stroke comprising aging and peripheral inflammation. Target prediction tools for human and mouse indicate that both miR‐127‐3p and miR‐127‐5p have several putative targets related to apoptosis. We verify 26S proteasome non‐ATPase regulatory subunit 3 (*Psmd3*) to be a target for mouse miR‐127‐5p. The increased neuronal vulnerability in aged mice subjected to peripheral inflammation led to impaired motor functions and importantly, we show that the levels of miR‐127 were downregulated also in the ischemic human brain, underlying the relevance of improved stroke modeling when conducting preclinical studies.

## 2 RESULTS

### 2.1 Aged mice subjected to peripheral inflammation showed significantly increased lesion size and aggravated ischemia‐induced deficits in sensory and motor functions

**Figure 1:**
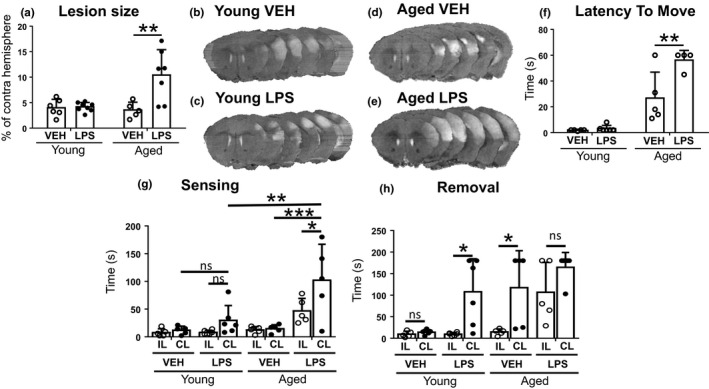
Aged LPS‐treated mice showed increased lesion size and aggravated ischemia‐induced motor and sensory deficits. The lesion size was significantly increased in the aged mice with peripheral inflammation induced by LPS compared to young mice with or without inflammation, or to aged mice without inflammation 24 h post‐injury (a). Representative MRI images of the young vehicle (b), young LPS (c), aged vehicle (d), and aged LPS‐treated (e) mice. Aged mice suffering from inflammation also showed aggravated deficits in motor coordination measured with Latency to move test at 1 day after the insult; they were significantly slower to move a distance equal to their own body length when compared to aged mice without inflammation (f). Adhesive removal test revealed that aged, LPS‐treated mice were slower in sensing the adhesive batch on the contralateral side, in comparison to contralateral sensing time of vehicle‐treated mice of similar age (g). Clear difference was observed as well when contralateral sensing time of aged LPS group was compared to ipsilateral sensing time of the same group, and to contralateral sensing time of young mice treated with LPS (g). Within the young mice, there was a trend of slower contralateral sensing in LPS‐treated group, but it did not reach statistical significance. When measured time to remove the batches (h), there was no difference between ipsi‐ and contralateral removal among the LPS‐treated aged mice, but instead vehicle‐treated aged mice were faster in removing the ipsi‐ than the contralateral batch. An opposite result to this was seen among the young mice, as LPS treatment seemed to slow down the contralateral removal. The data are shown as mean ± SD. VEH = Vehicle treatment, LPS = LPS treatment, IL = Ipsilateral, CL = Contralateral *p < 0.05 **p < 0.01 ***p < 0.001 (a) *F*(1, 22) = 8.76 *p* = 0.007 interaction effect; *F*(1, 22) = 6.59 *p* = 0.0176 age effect; *F*(1, 22) = 9.68 *p* = 0.005 treatment effect of two‐way ANOVA followed by Tukey's multiple comparisons test, adjusted *p* = 0.0023 *n* = 5–8 (f) *F*(1, 16) = 8.54 *p* = 0.010 interaction effect; *F*(1, 16) = 66.03 *p* < 0.0001 age effect; *F*(1, 16) = 10.28 *p* = 0.0055 treatment effect of two‐way ANOVA followed by Tukey's multiple comparisons test, adjusted *p* = 0.004 *n* = 4–5 (g) *F*(3, 34) = 13.63 *p* < 0.0001 age effect; *F*(1, 34) = 6.66 *p* = 0.014 treatment effect of two‐way ANOVA followed by Tukey's multiple comparisons test, adjusted *p* = 0.0388* *p* = 0.0015** *p* = 0.0002*** *n* = 5–6 (h) *F*(3, 34) = 10.68 *p* < 0.0001 age effect; *F*(1, 34) = 18.46 *p* = 0.0001 treatment effect of two‐way ANOVA followed by Tukey's multiple comparisons test, adjusted *p* = 0.030 in young LPS ipsi vs. contra, *p* = 0.0044 in aged vehicle ipsi vs. contra *n* = 5–6

To mimic comorbidity‐related peripheral inflammation, young and aged mice were subjected to intraperitoneal injection of LPS 30 min prior to permanent middle cerebral artery occlusion (pMCAO). Quantification of the MRI images at 24 h post‐stroke revealed significantly increased cell death specifically in aged mice subjected to LPS compared to any other treatment group (Figure 1a–e). Physiological parameters did not differ between the groups (data not shown).

Whereas LPS in young animals failed to aggravate the motor deficits, aged animals were significantly more vulnerable to stroke‐induced deficits in the sensory and motor coordination as measured by Latency to move (Figure 1f) and Adhesive removal tests (Figure 1g,h). Adhesive removal test revealed that aged, LPS‐treated animals were significantly slower in sensing the adhesive batch compared to any other groups (Figure 1g). In addition, aged, LPS‐treated mice showed a clear impairment in their ability to sense the adhesive batch in the contralateral paw compared to the ipsilateral paw. This deficit in sensing was not evident in any other study groups (Figure 1g). However, LPS‐induced reduction in the speed of the removal of the adhesive from the contralateral paw in young mice and this deficit was significant also in aged vehicle‐treated animals (Figure 1h). There were no differences between any of the treatment groups in the time it took for the mice to remove the adhesive batches from the contralateral side. Instead, the LPS‐treated aged mice were impaired also in their ability to remove the batch from ipsilateral paw, suggesting an overall impairment in the motor function of the mice.

### 2.2 Proinflammatory cytokines were elevated in the peri‐ischemic area of aged mice, and in plasma of the aged, LPS‐treated mice

**Figure 2:**
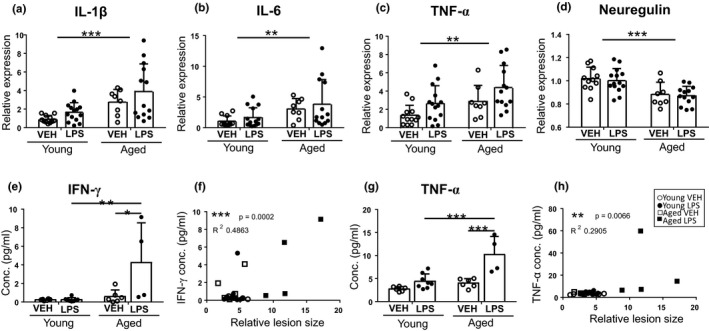
The mRNA levels of IL‐1β, IL‐6, TNF‐α, and Neuregulin were affected by age, and the protein levels of IFNγ and TNFα were increased by LPS treatment. The mRNA levels of proinflammatory cytokines IL‐1β (a), IL‐6 (b), and TNFα (c) were elevated in the PI cortical samples of the aged mice compared to young ones. The expression of Neuregulin, a neuronal growth factor showing neuroprotective properties, was significantly reduced in the aged mice compared to young mice (d). The peripheral inflammation did not affect significantly to the expression of these mediators (a‐d). The protein levels of cytokines were measured by CBA kit from plasma samples at 24 h post‐ischemia. Inflammatory cytokine IFNγ (e) was elevated in the group of aged mice with peripheral inflammation, and the concentration correlated significantly with the lesion size (the bigger the lesion, the higher the cytokine concentration) (f). Similar elevated concentration and correlation were also observed with TNFα (g, h). The data are shown as mean ± SD. VEH = Vehicle treatment, LPS = LPS treatment, *p < 0.05 **p < 0.01 ***p < 0.001 (a) *F*(1, 42) = 14.12 *p* = 0.0005 age effect of two‐way ANOVA, *n* = 8–14 (b) *F*(1, 42) = 7.88 *p* = 0.0075 age effect of two‐way ANOVA *n* = 8–14 (c) *F*(1, 43) = 8.23 *p* = 0.006 age effect; *F*(1, 43) = 6.19 *p* = 0.017 treatment effect of two‐way ANOVA *n* = 8–14 (d) *F*(1, 42) = 20.74 *p* < 0.0001 age effect of two‐way ANOVA *n* = 8–14 (e) *F*(1, 20) = 6.47 *p* = 0.019 interaction effect; *F*(1, 20) = 9.18 *p* = 0.0066 age effect; *F*(1, 20) = 6.51 *p* = 0.019 treatment effect of two‐way ANOVA, adjusted *p* = 0.0165* *p* = 0.0053** *n* = 4–8 (f) Pearson *r* = 0.697 *R* ^2^ = 0.486 two‐tailed *p* = 0.0002 *n*(*x*, *y*) = 24 (g) *F*(1, 20) = 8.19 *p* = 0.0096 interaction effect; *F*(1, 20) = 20.07 *p* = 0.0002 age effect; *F*(1, 20) = 24.70 *p* < 0.0001 treatment effect of two‐way ANOVA followed by Tukey's multiple comparisons test, adjusted *p* = 0.0003*** *n* = 4–8 (h) Pearson *r* = 0.539 *R* ^2^ = 0.291 two‐tailed *p* = 0.0066** *n*(*x*, *y*) = 24

qPCR revealed a significant age‐dependent increase in the mRNA levels of proinflammatory cytokines *IL*‐*1β*, *IL*‐*6*, and *TNFα* in the peri‐ischemic (PI) area of aged mice at 24 h post‐stroke compared to their young counterparts (Figure 2a‐c, respectively). In contrast, the mRNA levels of the neuronal growth factor neuregulin were significantly lower in the aged mice compared to the young (Figure 2d). LPS did not further alter the expression levels of these genes.

The levels of proinflammatory cytokine IFNγ were significantly elevated in the plasma samples of aged mice subjected to peripheral inflammation when compared to other treatment groups (Figure 2e) and significantly correlating with the lesion size (Figure 2f). A similar increase and correlation with the lesion size were also observed in the level of TNFα (Figure 2g‐h).

### 2.3 Peripheral inflammation increased microglial activation in the young mouse brains and induced neutrophil infiltration regardless of age, but failed to affect astrocytic activation, which was aggravated by age alone

As expected, LPS‐induced peripheral inflammation increased the expression of ionizing calcium‐binding adaptor molecule 1 (Iba1), a marker for activated microglia/macrophages, in the PI‐area of the ischemic young mice compared to their vehicle‐treated controls at 24 h after ischemia (Figure S1a). Surprisingly, in the aged mice, LPS‐induced peripheral inflammation failed to further increase Iba1 immunoreactivity (Figure S1a–e).

Staining of the brain sections against LY‐6B.2, a marker for neutrophils, revealed that whereas aging alone did not alter the number of neutrophils in the lesion area, peripheral LPS induced an increase in the number of neutrophil cell bodies in both young and aged LPS‐treated mice compared to their vehicle‐treated counterparts (Figure S2a–e) at 24 h post‐ischemia.

Aged mice irrespective of their predisposing inflammatory status showed significantly increased level of astrocytic GFAP immunoreactivity in the PI‐area compared to young vehicle or LPS‐treated mice at 24 h after stroke (Figure S3a–e).

### 2.4 The levels of miR‐127 were deregulated in the brains of aged, ischemic mice suffering from peripheral inflammation

**Figure 3:**
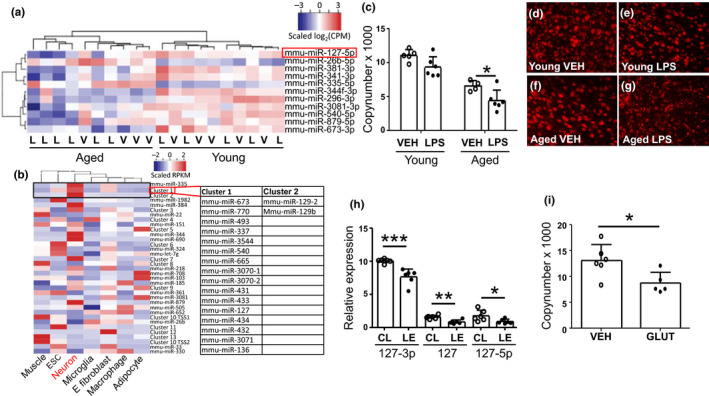
miR‐127 was dysregulated in the PI‐area of aged mice treated with LPS, in lesion samples from ischemic human brains, and in the cortical neuron culture treated with glutamate. Significant mature miRNA species in LPS‐treated aged mice (a). The mature miRNA species have been filtered to include only miRNAs derived from active loci based on neuron GRO‐seq data (b). Figure b depicts the tissue‐specific expression of miRNA loci in mouse. Significant miRNA loci in aged LPS‐treated mice where neuron‐specific transcription has been confirmed by GRO‐seq. For miRNA clusters, the cluster members are listed in Table S2. The three top loci represent top expressed, neuron‐specific miRNAs in neurons (ESC = embryonic stem cell, E fibroblast = embryonic fibroblast). MiR‐127 was significantly downregulated in brains of aged, ischemic mice with peripheral inflammation (c). Representative pictures of miR‐127 fluorescent in situ–hybridization from PI‐area of young vehicle (d), young LPS (e), aged vehicle (f), and aged LPS‐treated mice (g). Similar downregulation was observed as well in samples from human lesion area (h) and primary cortical neurons treated with glutamate 400 µM (i). The data are shown as mean±SD. V or VEH = Vehicle treatment, L or LPS = LPS treatment, CPM = counts per million, RPKM = reads per million kilobase, TSS = transcription start site, CL = contralateral, LE = lesion, GLUT = glutamate treatment 400 µM *p < 0.05 **p < 0.01 ***p < 0.001 (c) *F*(1, 17) = 69.64 *p* < 0.0001 age effect; *F*(1, 17) = 11.58 *p* = 0.003 treatment effect of two‐way ANOVA followed by Holm–Sidak's multiple comparisons test, adjusted *p* = 0.036 *n* = 4–6 (h) 127‐3p: *t*(10) = 4.84 *p* = 0.0007 *n* = 6 unpaired two‐tailed *t*‐test; 127: *t*(10) = 4.24 *p* = 0.002 *n* = 6 unpaired two‐tailed *t*‐test; 127‐5p: *t*(10) = 2.33 *p* = 0.042 *n* = 6 unpaired two‐tailed *t*‐test (i) *t*(9) = 2.69 *p* = 0.025 *n* = 5–6 unpaired two‐tailed *t*‐test

MiRNA sequencing was carried out to reveal the miRNA expression profile in the PI‐area, and to uncover possible mediators for the increased vulnerability to the ischemic brain damage in aged mice subjected to peripheral inflammation (Figure 3a). Stringent factorial statistical analysis fitting a four‐factor combination model resulted in 22 miRNAs being differentially regulated in the aged mice subjected to LPS compared to their young counterparts (with an adj. *p*‐value < 0.05) (Table S2). To exclude the age‐related effects we also compared LPS‐treated mice against vehicle treated in the young and aged group separately. Although not passing the significance threshold, multiple miRNAs were identified from these top tables indicating that more than natural changes in the miRnome were captured and possibly caused by a combinatorial effect of aging and inflammation. However, the expression levels of miRNAs from the PI area are resulting from several cell types harvested *in vivo*. The high variation among the expression levels between different cell types and different individual animals can drastically reduce the numbers of significant miRNAs detected, whereas they can be biologically significant. We therefore chose the 22 significant miRNAs for further filtering based on neuron‐specific miRNA expression.

To assess the tissue specificity of the differentially expressed miRNAs we looked at the primary miRNA transcription levels in seven mouse cell types to identify those specifically expressed in neurons. We generated GRO‐seq expression profiles for mouse primary neurons and microglia and assembled existing public GRO‐seq data for five other cell types including muscle cells, embryonic stem cells, macrophages, adipocyctes, and fibroblasts. Essentially, GRO‐seq identified 255 neuron‐expressed miRNA loci (Figure 3b), with miR‐335, miR‐127‐5p, and miR‐129‐2 being most abundantly expressed in neurons. Among them, miR‐127‐5p was embedded in a vast cluster of miRNAs on chromosome 12 with at least 15 confirmed miRNA species (Figure 3b). To exclude the expression from other cell types than neurons, 11 neuron‐specific miRNAs were chosen for further analysis based on GRO‐seq expression. Of the resulting 11 miRNAs, 5 were selected for validation by qPCR, based on abundance of their expression (data not shown).

From the set of validated genes, we were able to demonstrate that the level of miR‐127‐5p was significantly downregulated in the PI area of aged mice subjected to peripheral inflammation, when compared to their age‐matched, vehicle‐treated controls (Figure 3c). This was confirmed by *in situ* hybridization of miR‐127‐5p of the ischemic mouse brain sections (Figure 3d–g). The other investigated miRNAs or 3p‐arm of miR‐127 did not show significant differences in mouse samples. Since miR‐127 is conserved between rodents and humans, we next analyzed whether the expression of miR‐127 is altered in human stroke. Indeed, miRNA analysis of post‐mortem human brain showed a similar downregulation in the levels of miR‐127 (both 3p‐ and 5p‐arms) in the ischemic hemisphere compared to the unaffected contralateral hemisphere (Figure 3h). The expression level of miR‐127‐5p was also reduced upon glutamate exposure in primary cortical neurons, mimicking excitotoxic insult caused by stroke, compared to vehicle‐treated control cells (Figure 3i).

### 2.5 MiR‐127‐5p targets the expression level of Psmd3

**Figure 4:**
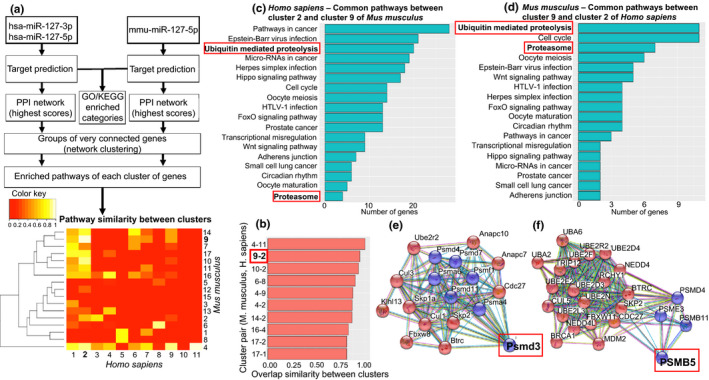
Pathway analysis of very connected targets of miR‐127 between human and mouse revealed proteasomes *Psmd3* and *PSMB5*. Workflow of the pathway analysis and heatmap showing pathway similarity between cluster couples of *H. sapiens* and *M. musculus* (a). Figure b illustrates the top ten couples, where mouse cluster 9 – human cluster 2 are among the three best pairs with over 90% similarity (b). The contents of these clusters are presented in (c) and (d), respectively. These clusters include proteasome genes *Psmd3* (e) and *PSMB5* (f)

In order to reveal targets for miR‐127, we took advantage of the TargetScan prediction tool and retrieved the predicted targets of mouse mir‐127 (5p‐arm) and human miR‐127 (both 3p and 5p arms) (Figure 4a). All the subsequent analysis were performed using R/Bioconductor (version 3.5.0). We used the package “STRINGdb” that provides an interface to the STRING protein–protein interaction database. Protein–protein interaction (PPI) networks of the predicted targets were built taking into account only the interactions with the highest interaction score. Starting from the human and mouse PPI networks of the predicted targets, we applied the community detection algorithm *walktrap* to discover very connected “clusters” of genes. For each cluster, we did KEGG functional enrichment to retrieve significantly enriched pathways with false discovery rate (FDR) of 5%. Next, we used *overlap coefficient* to compare the pairs of *Mus musculus* and *Homo sapiens* clusters and to find the best candidate pairs in terms of pathway similarity. For this analysis, only clusters with more than 15 genes were taken into account. Among the top three couples, we identified the cluster pair *M. musculus* cluster 9 – *H. sapiens* cluster 2 (Figure 4b), which include pathways”Ubiquitin mediated proteolysis” and”Proteasome” (Figure 4c,d). When exploring genes enriched within these pathways, proteasomes *PSMB5* and *Psmd3* (Figure 4e,f) were discovered. In addition, analyzing the modules of clusters 9 and 2 and focusing on the more connected ones, we discovered that both in human and mouse clusters, the genes *Psmd4*, *Btrc*, *Skp2*, *Cdc27*, and *Psmb11*, all involved in ubiquitin‐proteasome system in one way or another, are connected with proteasome genes *PSMB5* and *Psmd3* (Figure 4e,f). Since the proteasomes are known to be connected with inflammation, apoptotic processes, and caspase activity, they were chosen to further analysis. (See Appendix S1 for details.)

**Figure 5:**
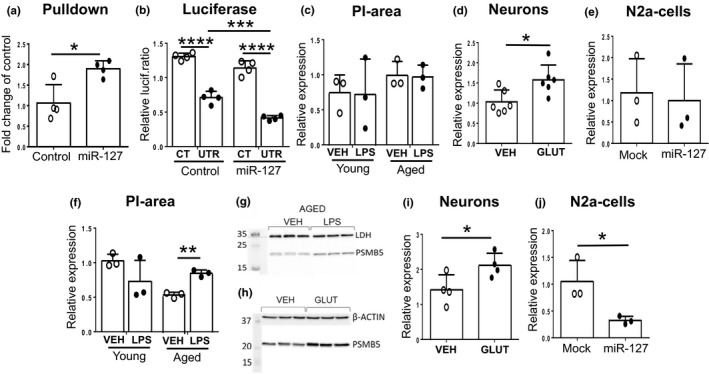
miR‐127‐5p targets proteasome *Psmd3*. Proteasome *Psmd3* was verified as a target for miR‐127‐5p by pulldown‐ (a) and luciferase assays (b). However, its protein levels were not changed in the peri‐ischemic area of young or aged mice with or without peripheral inflammation (c), but instead it was upregulated at mRNA level in primary cortical neurons treated with glutamate 400 µM (d). We failed to show downregulation of Psmd3 in N2a cells transfected with miR‐127 mimic (e). Although we were unable to show miR‐127‐5p binding to PSMB5, its protein levels were significantly upregulated in brains of aged, ischemic mice with peripheral inflammation (quantitative analysis f and western blot picture g) and in glutamate treated primary cortical neurons (western blot picture h and quantitative analysis i). PSMB5 was as well downregulated at mRNA level in N2a cells transfected with miR‐127 mimic (j). Cel‐miR‐39‐3p was used as a control‐miRNA (Control) in the pulldown assay (a). In the luciferase assay (b) the negative control mimic (Control) was Dharmacon miRIDIAN microRNA Mimic Negative Control #1, and negative control vector plasmid CmiT000001‐MT06 (GeneCopoeia) was used as the control plasmid (CT). Plasmid with 3′UTR Psmd3 (UTR) was miTarget™ miRNA 3′UTR Target Clone for mouse Psmd3 mRNA plasmid miT028449‐MT06 (GeneCopoeia). LDH and β‐actin were used as controls for western blots (f‐i). The data are shown as mean ± SD. VEH = Vehicle treatment, LPS = LPS treatment, PI = Peri‐ischemic, GLUT = Glutamate treatment 400 µM, Mock = Mock transfected, miR‐127 = miR‐127‐5p transfected, *p < 0.05 **p < 0.01 ***p < 0.001 ****p < 0.0001 (a) *t*(6) = 3.415 *p* = 0.0142 *n* = 4 unpaired two‐tailed *t*‐test (b) *F*(1, 12) = 36.50 *p* < 0.0001 miRNA effect; *F*(1, 12) = 298.0 *p* < 0.0001 plasmid effect of two‐way ANOVA followed by Tukey's multiple comparisons test, adjusted *p* < 0.0001**** *p* = 0.0007*** *n* = 4 (d) *t*(10) = 2.826 *p* = 0.0180 *n* = 6 unpaired two‐tailed *t*‐test (f) *t*(4) = 8.540 *p* = 0.001 *n* = 3 unpaired two‐tailed *t*‐test (i) *t*(6) = 2.545 *p* = 0.044 *n* = 4 unpaired two‐tailed *t*‐test (j) *t*(4) = 3.138 *p* = 0.035 *n* = 3 unpaired two‐tailed *t*‐test

The pulldown assay revealed that miR‐127‐5p was bound to *Psmd3* (Figure 5a), which was confirmed with luciferase assay (Figure 5b). Since the 3′UTR of PSMB5 lacks clear binding site for miR‐127‐5p, we were unable to verify the binding with these methods. To evaluate the expression levels of Psmd3 and PSMB5 in the peri‐ischemic samples, we carried out Western blotting to see whether their protein levels were altered in ischemic stroke in mice. Quantification of the Western blot showed that while Psmd3 remained unchanged (Figure 5c), the protein level of PSMB5 was significantly elevated in the peri‐ischemic area of the aged mice subjected to peripheral inflammation compared to the same area of their age‐matched, vehicle‐treated controls (Figure 5f,g). Next, the expression of these proteasomes was assessed in primary cortical neurons exposed to glutamate. In conditions where miR‐127‐5p was decreased, we showed that the levels of both Psmd3 (Figure 5d) and PSMB5 (Figure 5h,i) were increased. Finally, we evaluated the mRNA levels of *Psmd3* and *PSMB5* in N2a cells overexpressing miR‐127‐5p. To our surprise, *Psmd3* was again unchanged (Figure 5e), while *PSMB5* was significantly decreased in N2a cells transfected with mir‐127 mimic when compared to mock‐transfected cells (Figure 5j).

### 2.6 Significant alterations were observed in caspase‐3 immunoreactivity, along with apoptosis and proteasome activity

**Figure 6:**
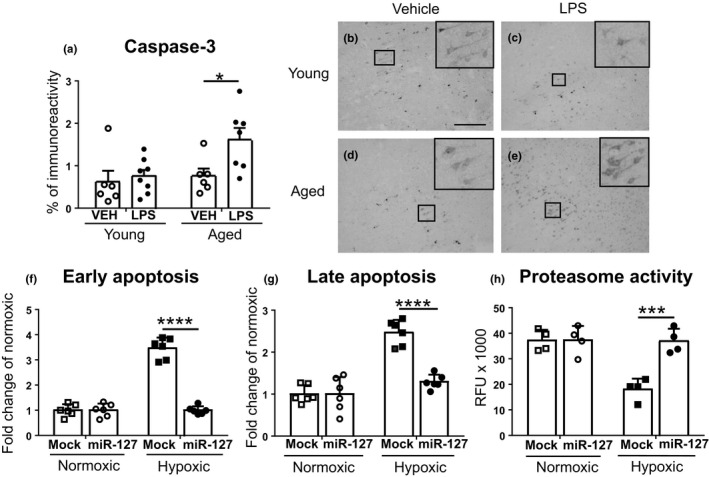
Caspase‐3 activity was highest among aged, ischemic mice with peripheral inflammation. Quantitative analysis (a) and representative images from the PI‐area of young vehicle (b), young LPS (c), aged vehicle (d), and aged LPS (e) mice (scale bar 100 µm). Apoptosis assay of transfected N2a cells exposed to hypoxia revealed that both early (f) and late (g) apoptosis activities were significantly higher among mock‐transfected cells, while in miR‐127 overexpressing cells they stayed on normoxic level. Chymotrypsin‐like proteasome activity of similarly transfected and exposed cells was drastically decreased in mock‐transfected hypoxic cells, and was increased back to normoxic level in miR‐127 transfected cells (h). The data are shown as mean ± SD. VEH = Vehicle treatment, LPS = LPS treatment, Mock = mock transfected, miR‐127 = miR‐127‐5p transfected *p < 0.05 ***p < 0.001 ****p < 0.0001 (a) *F*(1, 23) = 5.33 *p* = 0.030 age effect; *F*(1, 23) = 5.18 *p* = 0.032 treatment effect of two‐way ANOVA followed by Holm–Sidak's multiple comparisons test, adjusted *p* = 0.046 *n* = 6–8 (f) *F*(1, 20) = 111.8 *p* < 0.0001 interaction effect; *F*(1, 20) = 111.5 *p* < 0.0001 hypoxia and treatment effect of two‐way ANOVA followed by Tukey's multiple comparisons test, adjusted *p* < 0.0001 *n* = 6 (g) *F*(1, 20) = 25.11 *p* < 0.0001 interaction effect; *F*(1, 20) = 56.45 *p* < 0.0001 hypoxia effect; *F*(1, 20) = 24.9 *p* < 0.0001 treatment effect of two‐way ANOVA followed by Tukey's multiple comparisons test, adjusted *p* < 0.0001 *n* = 6 (h) *F*(1, 12) = 15.79 *p* = 0.0018 interaction effect; *F*(1, 12) = 16.87 *p* = 0.0015 hypoxia effect; *F*(1, 12) = 16.07 *p* = 0.0017 treatment effect of two‐way ANOVA followed by Tukey's multiple comparisons test, adjusted *p* = 0.0005 *n* = 4

To further evaluate the importance of apoptosis in the increased vulnerability of the aged mice predisposed to peripheral LPS‐induced inflammation, we carried out immunostaining against caspase‐3. Specifically, aged mice subjected to peripheral LPS‐induced inflammation exhibited significantly increased level of caspase‐3 immunoreactivity at the PI‐area (Figure 6a–e). Next, we conducted apoptosis assay on N2a cells transfected with miR‐127 mimic and exposed to hypoxia, to examine the effect of miR‐127 on apoptosis in vitro. The results showed that both early (Figure 6f) and late (Figure 6g) apoptosis activity was significantly higher among mock‐transfected cells, while in miR‐127 overexpressing cells it stayed on normoxic level. After this, we used similarly transfected and exposed cells to assess proteasome activity after hypoxia, expecting it to be lower in cells overexpressing miR‐127, according to our finding showing decreased PSMB5 level in miR‐127 transfected cells (Figure 5h). PSMB5 protein is also known as 20S proteasome subunit β5, containing chymotrypsin‐like activity (Coux, 1996). Contrary to our expectations, chymotrypsin‐like proteasome activity was markedly increased along with miR‐127 overexpression in hypoxic cells (Figure 6h).

## 3 DISCUSSION

The heterogeneity of stroke‐induced effects in patients causes a main hurdle in efficient drug discovery for ischemic stroke. Not only are most of the patients elderly, but also affected by multiple conditions influencing the peripheral immune system, causing the immune cells to prime themselves toward an inflammatory state. In clinical trials these priming conditions cause unexpected responses, thereby influencing the pathways targeted by the novel therapeutics. This possibly explains the failure to translate the preclinical findings to efficient therapeutics. It is thus of high importance to conduct studies assessing the contribution of immune system priming in combination with aging in preclinical stroke research.

Aging induces alterations in the vasculature, energy metabolism, inflammation status, and regeneration capability in the brain in both humans and animal models (Pizza et al., 2011). Our results show that aged mice exhibit lower levels of neuregulin in the PI‐area, suggesting decreased neuroregeneration and impaired brain repairment via reduced myelination, since neuregulin is shown to have a significant effect on myelinating oligodendrocytes (Lundgaard et al., 2013; Wang et al., 2007). Higher levels of proinflammatory gene products implying increased inflammation were as well observed in the PI‐area of the aged mice. This is in line with a previous study showing increased brain levels of TNFα after injury in aged animals (Le et al., 2014), although also contradictory findings have been reported (Sieber et al., 2011). Importantly, aging has been shown to prime cells of the peripheral immune system both in humans and in animals, demonstrated as increased levels of plasma TNFα along with activated macrophages (Pizza et al., 2011; Salminen et al., 2011). Moreover, hypercholesterolemic mice show elevated ischemia‐induced plasma levels of TNFα and worsened outcome after stroke (Herz et al., 2014). Our data of the increased levels of IFNγ and TNFα in the plasma specifically in the aged, LPS‐treated mice with correlation to the lesion size may thus have clinical relevance as stroke patients have increased plasma levels of TNFα, which are correlated with the infarct volume (Martínez‐Sánchez, 2014). Indeed, inflammatory markers have diagnostic value as predictors of the severity of the outcome of stroke (Bokhari et al., 2014).

Respiratory tract infections, among others, are not only associated with increased risk of ischemic events, but infections shortly preceding the ischemic stroke also worsen the outcome (Grabska et al., 2011). However, the number of preclinical studies assessing the effect of pre‐ischemic inflammatory conditions on the outcome of stroke is limited. Low dose of peripheral LPS aggravates ischemia‐induced neuronal death in a transient mouse model of cerebral stroke (McColl et al., 2007), impairs motor functions, and increases BBB permeability (Doll et al., 2015). In line with these findings, we have previously reported that aged mice subjected to low‐grade peripheral infection induced by *Trichuris Muris* parasite administration show aggravated neuronal death upon ischemic stroke (Dhungana et al., 2013). Here we extent these findings using LPS as a model of peripheral inflammation to report also impairments in motor functions specifically in the aged animals. Increased neuronal damage in aged, infected mice was associated with increased neutrophil infiltration. Considering these published findings, we were surprised to see that LPS‐induced a massive neutrophil infiltration into the ischemic core regardless of age with no correlation with extent of neuronal death. The increased lesion size was not associated with altered brain inflammatory response which is surprising since astrocytes and microglia possess significant roles in the post‐ischemic cascade of events—either detrimental or beneficial. These differences are likely to be explained by the different model for inducing the peripheral inflammation. In addition, the stroke pathology is significantly different between the transient and the permanent models, highlighting the importance of investigating pathological mechanisms across different models and conditions.

To discover the molecular mediators underlying the increased neuronal vulnerability in aged, LPS‐treated mice, we integrated nascent RNA and mature miRNA expression sets to characterize the murine neuronal miRnome under conditions of aging, ischemic stroke, and peripheral inflammation. Nascent transcriptome data obtained by the GRO‐seq assay identified 255 active genomic regions producing primary miRNA transcripts in primary neurons. These data were then used to extract top candidates from miRNA‐seq, which was obtained from the PI site of the brain post‐stroke and thus contained every brain cell type. From the top 3 miRNA loci highly expressed in neurons, we further focused on miR‐127 from Cluster 1. The other two loci were mmu‐miR‐335, and Cluster 2 harboring miR‐129 family precursors. MiR‐129 has been identified in mouse brain tissue (Lagos‐Quintana, 2002), yet it was not found to be significantly altered in the miRNA‐seq analysis. Although there is no GRO‐seq data available for mouse astrocytes or oligodendrocytes, existing reports show that mmu‐miR‐335 is expressed in astrocytes and implicated in astrocytoma development (Shu et al., 2011). This suggested that astrocytes could also have contributed to the miR‐335 expression profile in the miRNA‐seq dataset. We carried out qPCR measurements for miR‐127 in cultured mouse primary astrocytes and microglia, and failed to detect miR‐127 in these cell types (data not shown). Our data showing downregulation of miR‐127‐5p in the brains of aged mice suffering from stroke and peripheral inflammation, and our GRO‐seq complemented with qPCR data on primary cell cultures suggest it to be very neuron specific in mice. The reduction in the levels of miR‐127 is also clinically relevant, as both miR‐127‐3p and miR‐127‐5p were significantly downregulated in human stroke brain samples. However, in humans the neuron specificity cannot be confirmed, since miR‐127‐3p expression is reported in context of gliomas (Jiang et al., 2014), and available human datasets show miR‐127 expression in astrocytes, while data from neurons and microglia are lacking. In *in vivo* situation, all brain cell types are affected. Especially astrocytes can either aid neurons to survive by releasing neurotrophic factors (Nicole et al., 2001), or aggravate the lesion formation by turning into glial scar in the acute phase (McKeon et al., 1991). Oligodendrocytes are more sensitive to deprivation of oxygen and nutrients than other glia (Giacci et al., 2018), and death of oligodendrocytes leads to demyelination and axonal degeneration, making all matters worse. Because of this, effects of miR‐127 in especially human oligodendrocytes and glial cells need further clarification. Until now, miR‐127 has been shown to be beneficial in human keratocytes upon LPS exposure (Li et al., 2018) and miR‐127‐3p has been suggested as a biomarker for frontotemporal dementia (Piscopo et al., 2018), yet nothing is known about the function of miR‐127 in the brain during hypoxic and inflammatory conditions.

To evaluate the pathways targeted by miR‐127, we carried out pathway analysis of the predicted targets for both arms for human and 5p‐arm for mouse, and pinpointed proteasome *Psmd3* as a target for miR‐127‐5p. Proteasome dysfunction is evidently connected with apoptosis (Low, 2006), which is a central feature in stroke‐induced pathology. Activity of proteasomes tends to decrease not only with normal aging, but also in neurodegeneration (Vilchez et al., 2014), during inflammation (Stanimirovic et al., 2001) and with prolonged hypoxia (Schmidt‐Kastner et al., 2002). Impaired proteasomal activity is known to markedly alter expression of genes having an important role in aging and neurodegeneration (Ding et al., 2004), for example increasing neuronal pentraxin 1 which is mediating brain inflammation in aging and AD (McGeer, 2001) as well as inducing neuronal apoptosis (DeGregorio‐Rocasolano et al., 2001).

Ubiquitin‐proteasome system (UPS) is known to regulate a vast variety of apoptosis‐related proteins (Gupta et al., 2018), and inhibition of proteasome activity has been stated to promote apoptosis (Qiu et al., 2000). On the other hand, proteasome activity inhibition has as well reported to have anti‐inflammatory effects (Meng et al., 1999) and it is estimated to be beneficial in ischemic stroke (Di Napoli, 2003). Proteasomal activation may also aid cell survival under conditions of oxidative stress; one example is a study from Kwak and Kensler (2006) examining upregulation of PSMB5 in N2a‐cells (Kwak & Kensler, 2006). These seemingly contradictory results may arise from different models used in the experiments, or alternatively, some of the studies focusing on the 26S proteasome. 26S is the central factor in the UPS, and it consists of two 19S regulatory parts and one 20S core (Tanaka, 2009). Psmd3 is part of the 19S unit, whereas 20S core accommodates PSMB5. Proteolytic activity of 26S proteasome is impaired during ischemia due to ATP depletion, which causes inactivation of the 19S unit, leaving only 20S core functional under oxidative stress (Reinheckel et al., 1998). This may explain our findings showing miR‐127 targeting Psmd3, but the levels of which being inconsistent with the expression of miR‐127. Despite of PSMB5 lacking the known binding site for miR‐127‐5p, we detected downregulation of PSMB5 in miR‐127 overexpressing cells but upregulation in 20S activity in the same cells, which may as well be due to disassembly of the 26S proteasome, and/or possible compensatory mechanisms due to miR‐127 overexpression. Overexpression may cause unspecific binding to PSMB5, but there may as well be currently unknown mechanisms of miRNA actions behind our results. Within this study, we were unable to clarify this, but this issue indeed deserves further attention.

It is plausible that neuroprotective effect of miR‐127 is mediated through nuclear factor‐kappa B (Nf‐κB): 20S proteasome targets phosphorylated I‐κB and degrades it, which activates Nf‐κB (Carroll et al., 2000). Although Nf‐κB activation in glial cells is known to have detrimental consequences (Saggu et al., 2016), in neurons it is involved in synaptic plasticity and neuronal survival, and it is triggering an endogenous caspase inhibitory system (Mattson, 2005). This is in line with our in vivo findings and as well with in vitro results showing decreased apoptosis and increased 20S activity.

In conclusion, here we integrate GRO‐seq data with mature miRNA sequencing to identify the neuron‐specific miRNome and pinpoint a previously uninvestigated miRNA in the brain, miR‐127, that was highly expressed in neurons and was associated with increased cell death in the aged, LPS‐injected ischemic mice. We demonstrated that miR‐127‐5p interacts with *Psmd3*, and has an effect on cellular apoptosis and proteasomal activity in in vitro conditions mimicking ischemic neuronal damage. To provide clinical relevance for our findings, we also reported that the levels of miR‐127 are similarly decreased in human stroke brains. Although it is likely that targeting miR‐127 is not effective in preventing stroke‐induced neuronal death in young animals, our results underline the impact of peripheral inflammation on the outcome stroke in aging and pinpoint molecular targets for restoring endogenous neuronal capacity to combat ischemic stroke specifically in aged subjects.

## 4 EXPERIMENTAL PROCEDURES

See Appendix S1 for detailed description of all procedures described below.

### 4.1 Animals

Altogether 60 young (3–6 month‐old) and 60 aged (18–24 month‐old) C57Bl/6 J mice were used for the study. 25% of the mice were females, and they were divided equally to treatment groups (young vehicle, young LPS, aged vehicle, and aged LPS treated). The results of the females did not differ from the males, so they are not separated in the analysis. The mice were housed individually in controlled temperature, humidity, and light conditions (12 h light/dark cycles). Water and food were provided ad libitum. The randomization into treatment groups was done according to the RIGOR criteria by using GraphPad QuickCalcs software (www.graphpad.com/quickcalcs/, GraphPad Software, San Diego, CA, USA) and all analyses were performed blinded to the study groups. The exclusion criteria were pre‐set: hemorrhages visible in MRI, bleeding during the surgery, unsuccessful induction of ischemia, and statistically significant outlier in any of the analysis (calculated with GraphPad QuickCalcs Outlier calculator, GraphPad Software, La Jolla, CA, USA) led to the exclusion of the animal. Total of 22 mice (7 of young and 15 of aged) were either excluded according to these criteria or died during the study. All applicable National Animal Experiment Board of Finland and the Council of Europe Legislation and Regulation for Animal Protection guidelines for the care and use of animals were followed.

### 4.2 Induction of acute peripheral inflammation

Acute peripheral infection was induced by intraperitoneal (i.p.) injection of LPS (100 µg/kg, serotype 0127:B8 Sigma, St. Louis, MO, USA) or vehicle (0.9% sterile saline, Baxter, Deerfield, IL, USA) 30 min before pMCAO.

### 4.3 Ischemia surgery, sample collection, and analysis

The left MCA was permanently occluded as described in Appendix S1 and in Dhungana et al., 2013. At 1 day post‐infarction, the mice went under Latency to move and Adhesive removal tests to evaluate stroke outcome, and MRI imaging to measure the size of the lesion. After these procedures, the mice were transcardially perfused and the brains were collected either for immunohistochemistry to stain for astrocytic, microglial, and caspase‐3 activation and neutrophil infiltration, or in situ hybridization for miR‐127. Alternatively, the PI‐area of the brain was dissected for qPCR for inflammatory markers, miRNAs, and RNAs of interest and for WB to evaluate protein levels of PSMB5 and Psmd3. RNA from these samples was as well used to generate microRNA NGS libraries for next‐generation sequencing. Plasma samples were collected for cytokine measurements by Cytokine Bead Array.

### 4.4 In vitro experiments

Primary cortical neuron cultures were used for glutamate exposures to mimic the exitotoxic insult caused by stroke. The cells were collected for protein and RNA to use in qPCR and WB analysis. These samples were also used along with samples from BV2 cells to perform GRO‐seq assay. Mouse neuroblastoma (N2a) cells were transfected with miR‐127 mimics and exposed to hypoxia, and the samples were used to evaluate apoptotic activity, proteasomal activity, and levels of Psmd3 and PSMB5. MiRNA pulldown assay and luciferase assay to verify targets of miR‐127 was as well conducted with these cells.

### 4.5 Human brain samples

Brain samples from 6 patients who died from an ischemic stroke were included in this study (Table 1). Briefly, post‐mortem tissue samples from the infarct core (IC) and the contralateral (CL) areas were collected, preserved immediately by snap freezing in liquid nitrogen, and stored at −80°C until further use. All procedures performed in this study were in accordance with the ethical standards of the Ethics Committee of the Vall d’Hebron Hospital (PR[HG]85/04) and informed consent was obtained from relatives in accordance with the 1964 Helsinki declaration and its later amendments.

**Table 1:** Demographic and clinical data from ischemic stroke patients

Patient	Sex	Age (year)	T.O.D. (h)	PMI (h)	r‐tPA	Hemisphere
N16	F	79	88	5	No	Left
N22	M	67	62	7	Yes	Left
N32	M	73	360	6	No	Left
N33	M	80	100	4.5	No	Right
N35	M	84	40	8	No	Left
N36	F	73	44	4	Yes	Left

Abbreviations: F, female; M, male; PMI, post‐mortem interval, from death to sample collection; r‐tPA, recombinant tissue‐plasminogen activator; T.O.D, time from onset of stroke symptoms to death.

### 4.6 Statistics

Statistical analyses were done with GraphPad Prism software 5.03 (GraphPad Software, La Jolla, CA, USA) using two‐way ANOVA without repeated measures (followed by Tukey's or Holm–Sidak's multiple comparisons test, where appropriate) or by unpaired two‐tailed *t*‐test. The statistical tests used are indicated in each figure legend. *p*‐values < 0.05 were considered as significant. The data are presented as mean ± standard deviation, and equality of mean and median values in representing the data has been confirmed for each dataset.

## CONFLICT OF INTEREST

The authors do not have any conflicts of interests to declare.

## AUTHOR CONTRIBUTIONS

Sanna Loppi and Paula Korhonen contributed equally to all experiments and preparation of the manuscript.

## Supporting information

Fig S2

Fig S1

Fig S3

Table S1

Table S2

Supplementary Material

## Data Availability

The data that support the findings of this study are available from the corresponding author upon reasonable request.
