# Supplementary figures and images for: Peripheral inflammation preceeding ischemia impairs neuronal survival through mechanisms involving miR‐127 in aged animals
Source: Aging Cell. 2020 Dec 28;20(1):e13287. doi: 10.1111/acel.13287 (PMC7811844; doi:10.1111/acel.13287)

## Slide 1
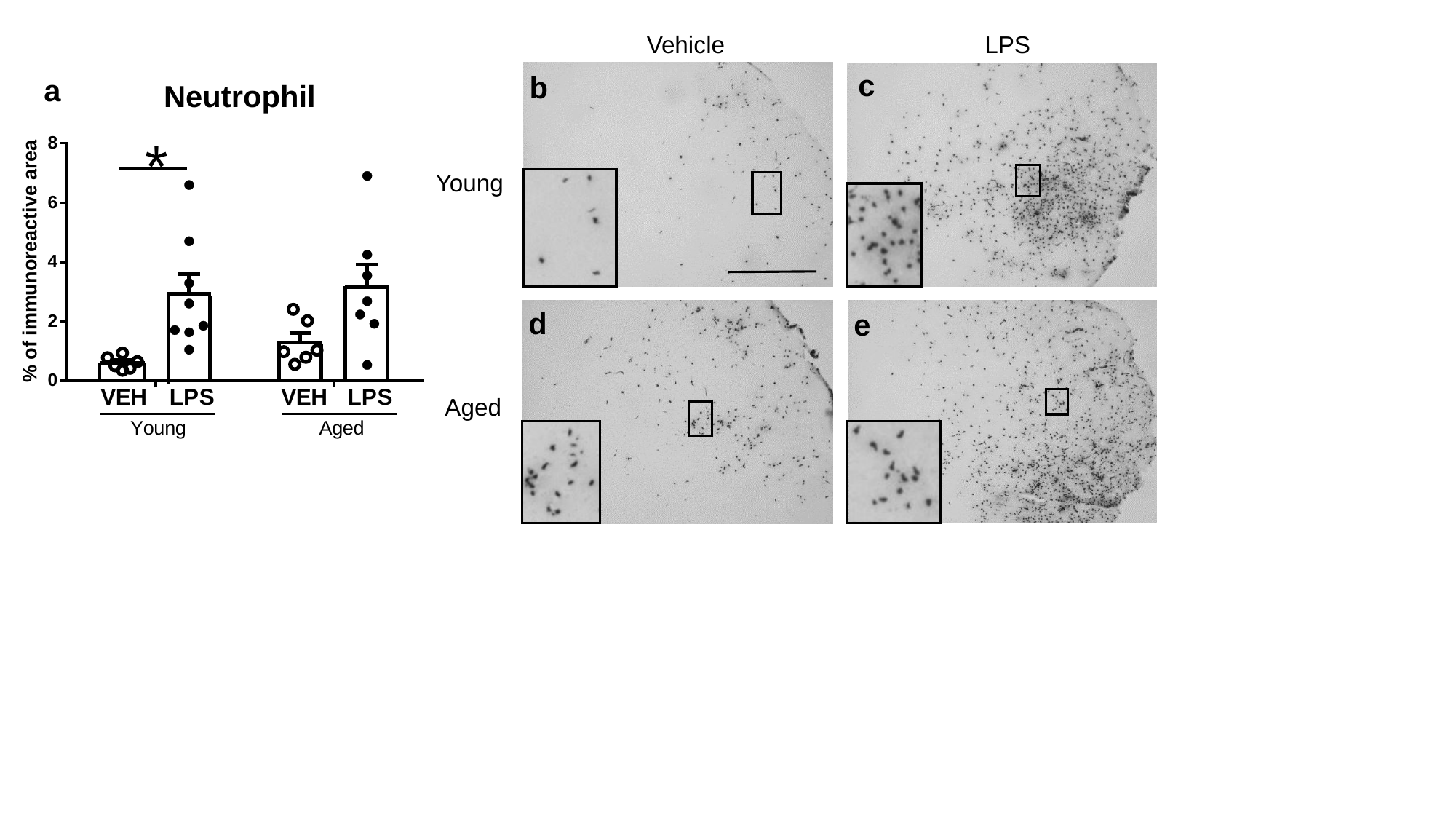

Vehicle
LPS
c
b
Young
d
e
Aged
a
Neutrophil

## Slide 2
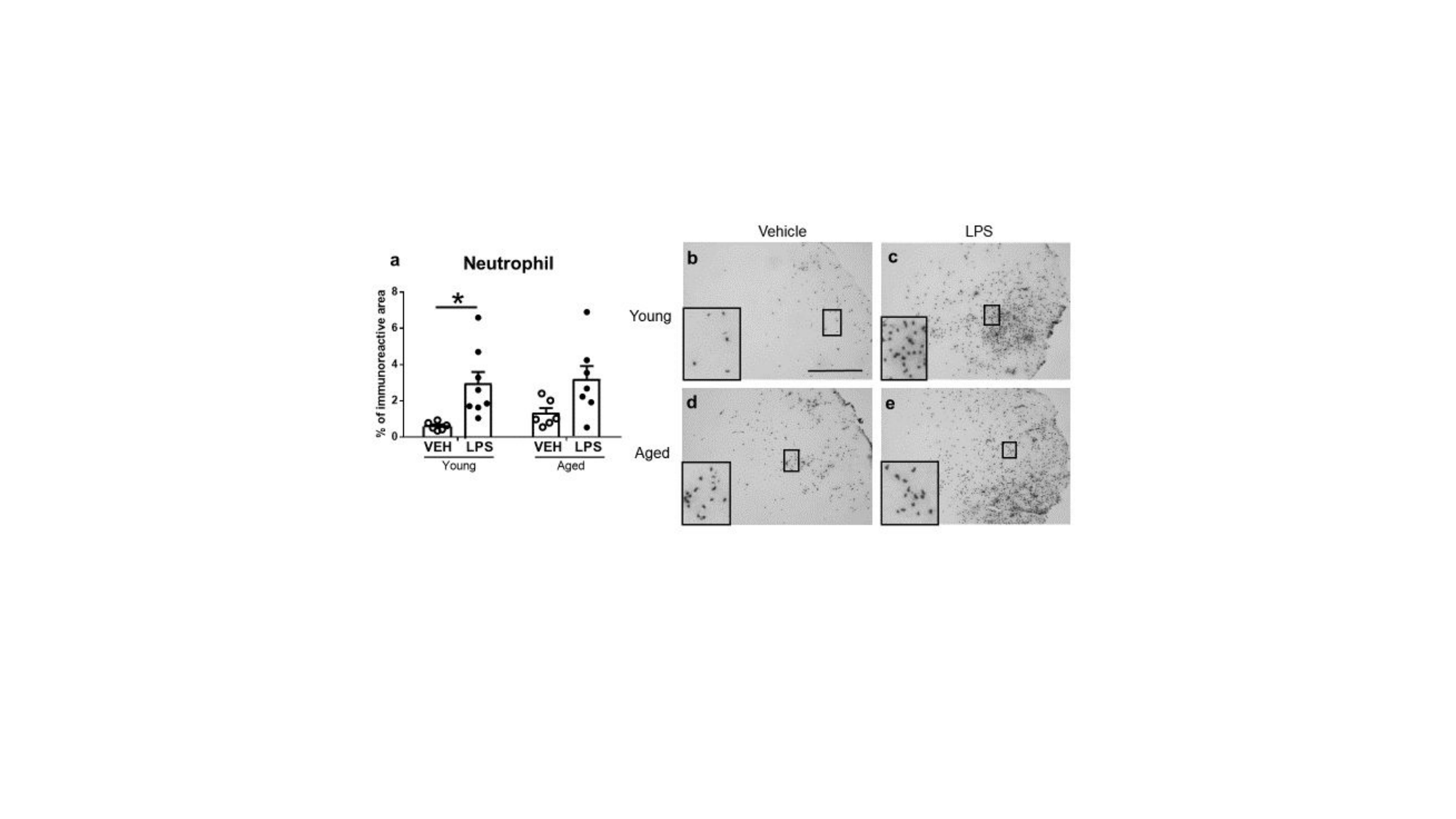

Supplement: Supplementary file 1 — Fig S2 [file ACEL-20-e13287-s001.pptx]

## Slide 1
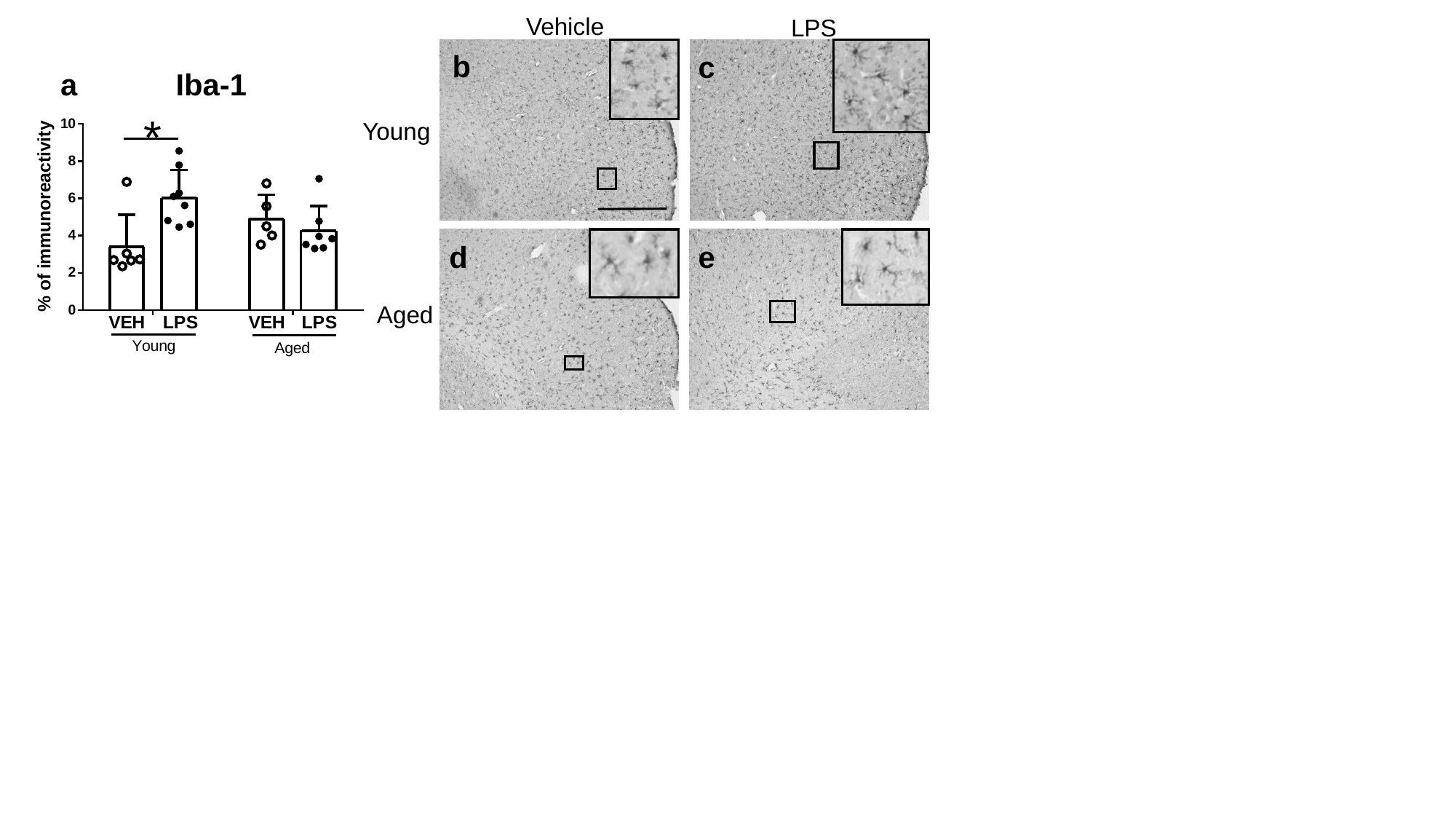

Vehicle
LPS
b
c
Young
d
e
Aged
a
Iba-1

## Slide 2
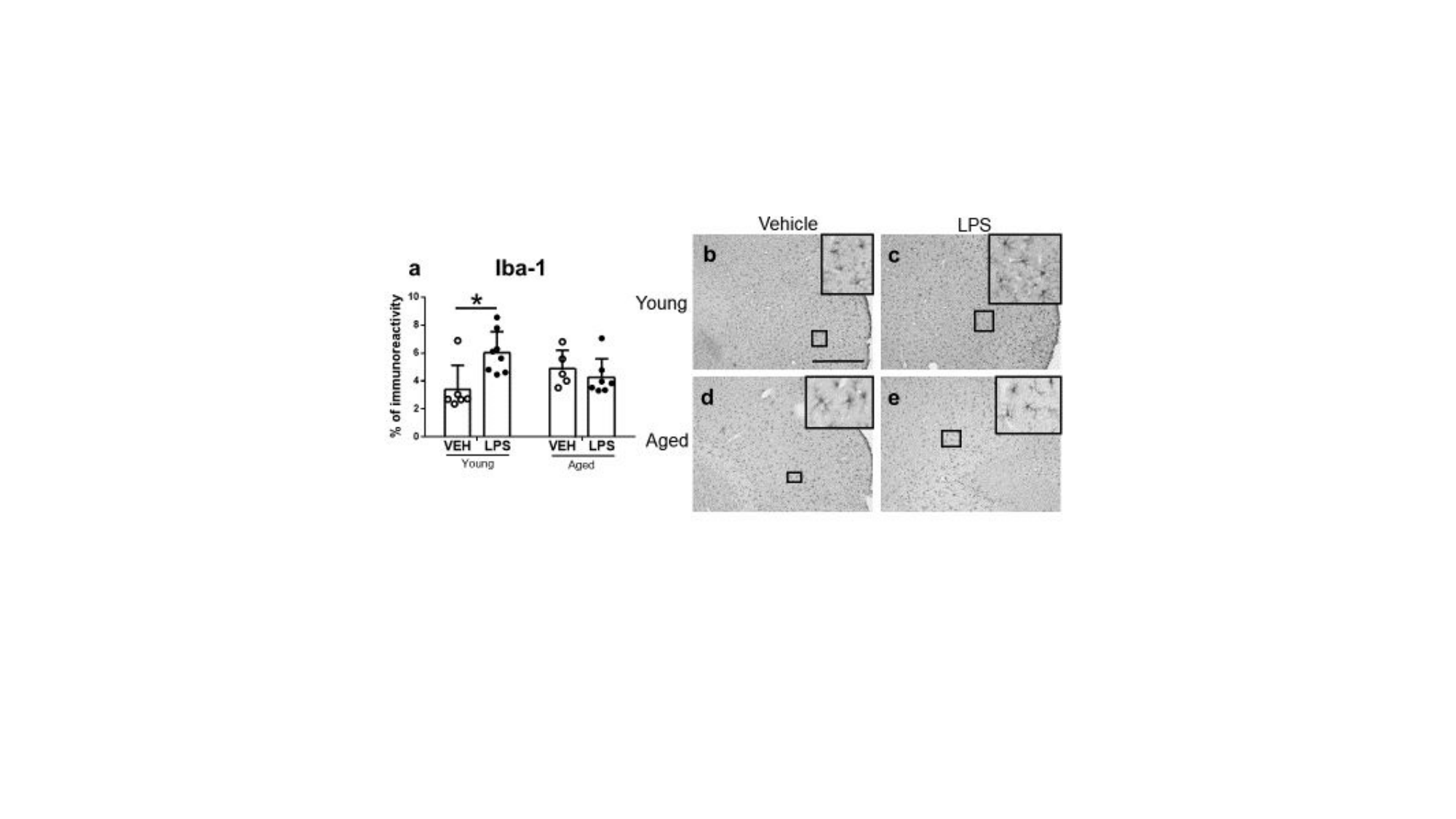

Supplement: Supplementary file 2 — Fig S1 [file ACEL-20-e13287-s002.pptx]

## Slide 1
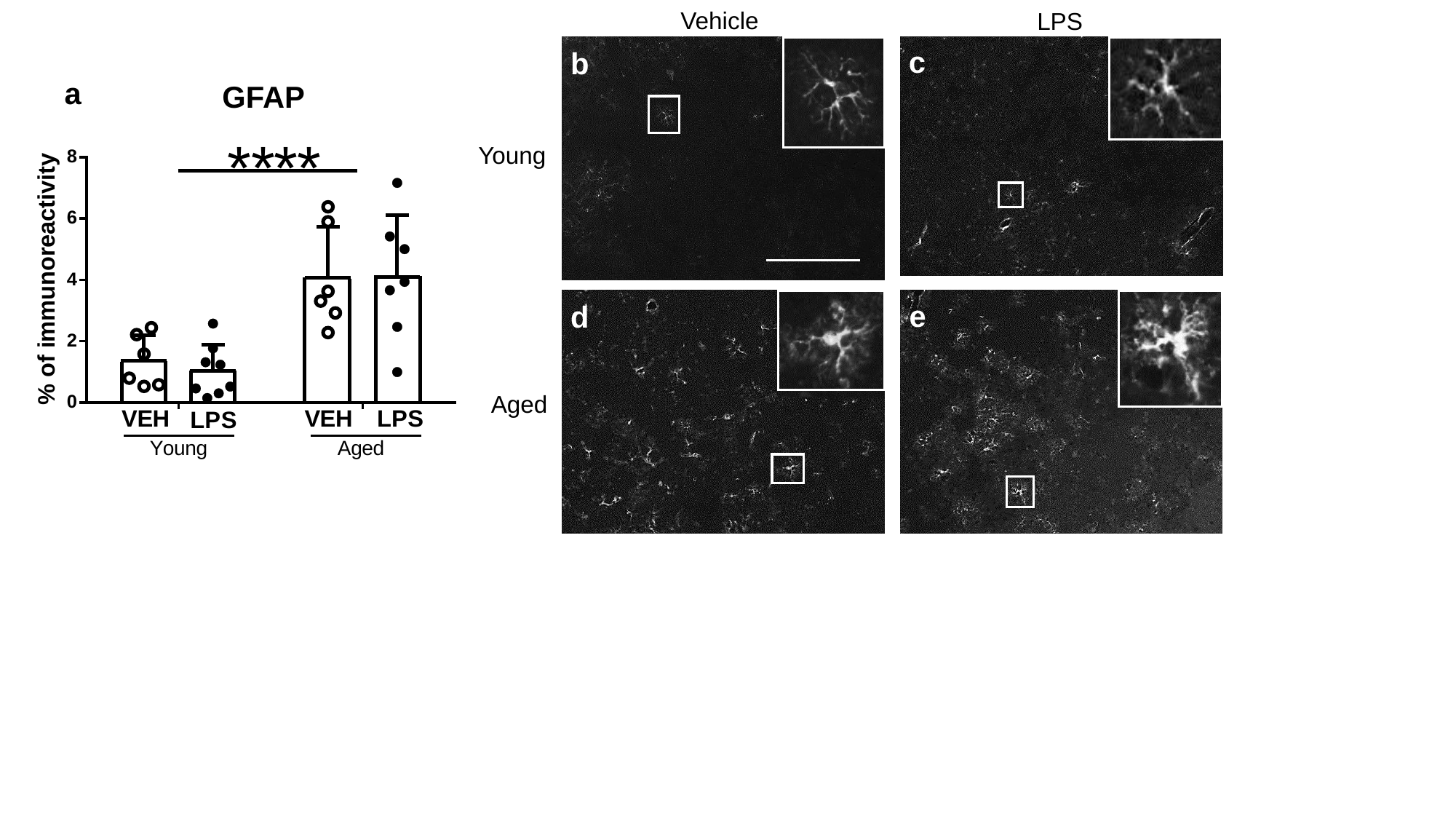

Vehicle
LPS
c
b
Young
e
d
Aged
a
GFAP

## Slide 2
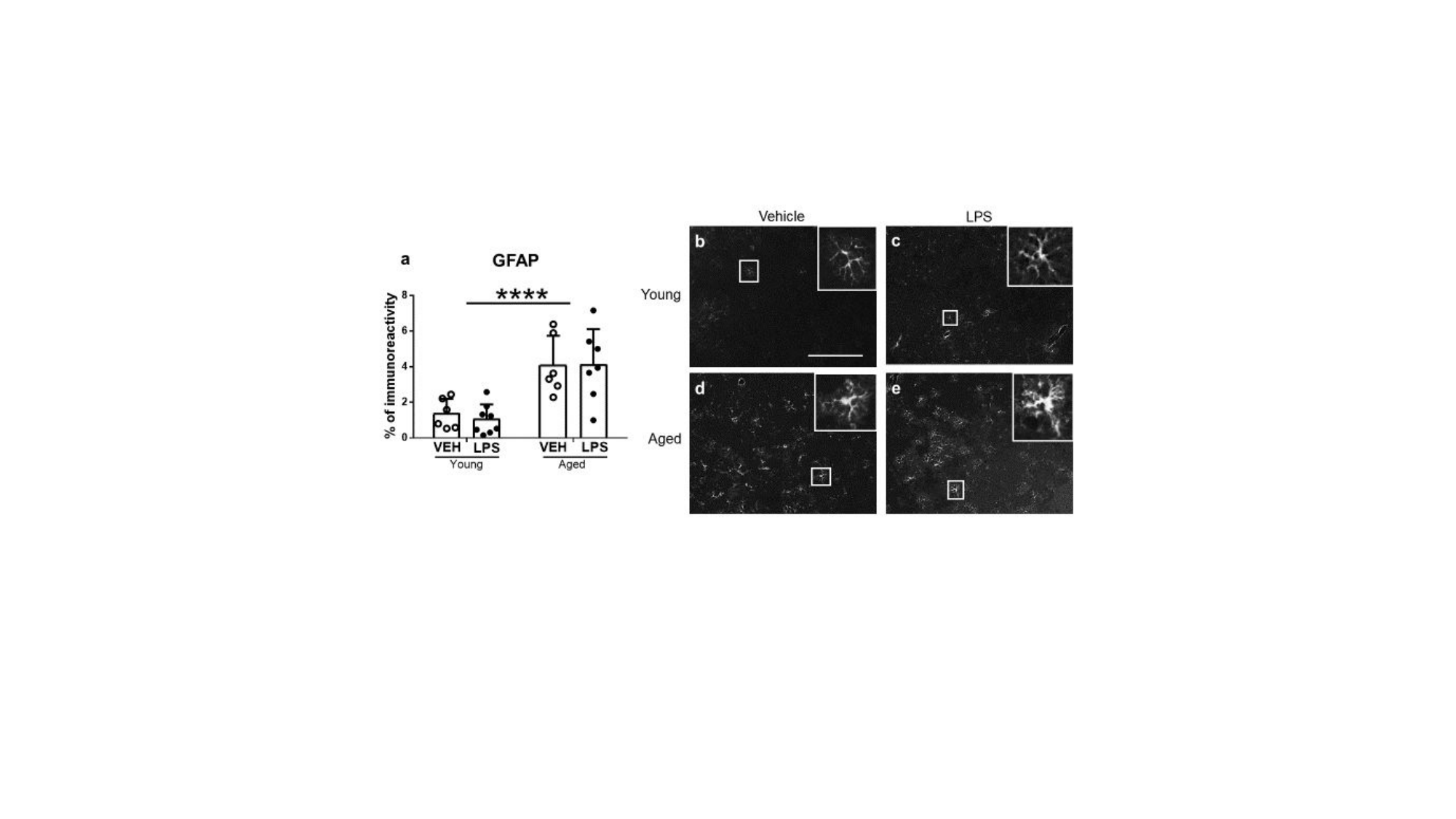

Supplement: Supplementary file 3 — Fig S3 [file ACEL-20-e13287-s003.pptx]
