# Supplementary material for: Peripheral inflammation preceeding ischemia impairs neuronal survival through mechanisms involving miR‐127 in aged animals
Source: Aging Cell. 2020 Dec 28;20(1):e13287. doi: 10.1111/acel.13287 (PMC7811844; doi:10.1111/acel.13287)

**Supplementary material**

**Detailed description of experimental procedures**

*Ischemia surgery*

The left middle cerebral artery (MCA) was permanently occluded as previously described (Dhungana et al., 2013). Anaesthesia was induced by 5% isoflurane in 30% oxygen and 70% nitrogen as a carrier gas and maintained with 2% isoflurane during the surgical operation. The temperature was kept constant (+37 ± 1 °C) using the heating blanket connected to the rectal probe (Harvard apparatus, PanLab, Barcelona, Spain). The temporal muscle was detached from the scull and 1-mm hole was drilled to temporal bone. The dura was carefully removed and the exposed MCA was lifted and occluded using a thermocoagulator (Aaron Medical Industries Inc., Clearwater, FL, USA). The success of the occlusion was confirmed by cutting the artery after which the temporal muscle was placed back on top of the hole and the skin was sutured. The mice were returned to their home cages to recover from the surgery.

*Physiological parameters*

The physiological parameters were measured from the blood samples taken from the saphenous vein immediately after the surgery. Freestyle blood glucose monitoring system (Abbott, Alameda, CA, USA) was used to assess the glucose levels and iSTAT analyzer (Abbott, Abbott Park, IL, USA) to measure the partial pressure of carbon dioxide and oxygen and the pH.

*Post-surgery evaluation of outcome*

Evaluation of motor and sensory deficits at one day after stroke was performed by using the Latency to move – and Adhesive removal -tests. The tests were carried out blinded to the study groups. Latency to move -test (Bargiotas, Krenz, Monyer, & Schwaninger, 2012) started with baseline measurements carried out one day prior to the induction of ischemia and the actual measurements were performed at day 1 post injury prior the MRI imaging. Mice (8-12 per treatment group) were placed on a flat surface and the time to move one body length (7cm) was recorded. For Adhesive removal test (Bouet et al., 2009) each mouse was given four training sessions before induction of ischemia. In the first training the mice were habituated to the testing environment without adhesive tapes, and in second and third trainings the mice were placed into the test box with adhesives attached to both of their front paws. The mice were allowed to learn how to take them off, and by the fourth training session (one day before induction of ischemia) all mice were able to remove the adhesives from both paws within seconds. This last training session was used to record the baseline time for sensing and removing the batches. The actual test was carried out at day 1 post stroke prior the MRI imaging.

*Magnetic resonance imaging*

The lesion volume was measured *in vivo* at 24 h post ischemia by magnetic resonance imaging using a vertical 9.4 T Oxford NMR 400 magnet (Oxford Instrument PLC, Abington, UK) as previously described (Dhungana et al., 2013). Briefly, the mice were anesthetized with 5 % isoflurane and the anesthesia was maintained with 1 % isoflurane during the imaging procedure. Multislice T2-weighted images (repetition time 3000 ms, echo time 40 ms, matrix size 128 × 256, field of view 19.2 × 19.2 mm^2^, slice thickness 0.8 mm and number of slices 12) were taken and the obtained images were analyzed with in-house made Aedes software under the Matlab environment (Math-works, Natick, MA, USA). The infarct volume was quantified as using a previously described formula: *infarct volume = (volume of contralateral hemisphere − (volume of ipsilateral hemisphere − measured infarct volume))/volume of contralateral hemisphere* (Shuaib, Xu Wang, Yang, & Noor, 2002).

*Tissue dissection*

All mice were sacrificed at 1dpi by transcardial perfusion using heparinized saline (2500 IU/ml). The brains were removed and post-fixed in 4% paraformaldehyde for 20–22 h followed by cryoprotection in 30% sucrose for 48 h and frozen on liquid nitrogen. The frozen brains were cut to 20-μm-thick sections using a cryostat (Leica Microsystems, Wetzlar, Germany). Alternatively, after transcardial perfusion the brains were removed and the peri-ischemic area corresponding to approximately 1 mm area surrounding the lesion were dissected. The samples were snap-frozen in liquid nitrogen for further analysis.

*Immunohistochemistry*

Astrocytic activation was assessed by a glial fibrillary acid protein (GFAP, 1:500 dilution, DAKO, Agilent, Santa Clara, CA, USA) and microgliosis using ionized calcium-binding adapter molecule-1 (Iba-1, 1:250 dilution, Wako Chemicals, Tokyo, Japan) as a marker. The neutrophil infiltration was visualized by Ly-6B.2 antibody (1:250, Serotec, Oxford, UK) and caspase-3 using cleaved Caspase-3 antibody (Cell Signaling Technology, Leiden, Netherlands). The brain sections were incubated with primary antibodies overnight at room temperature. Next day, after 3 washes with 0.05% Tween in PBS, the sections were incubated for two hours with an appropriate secondary antibody. Fluorescent Alexa 568-conjugated antibody was used for GFAP (1:200, Life Technologies, Carlsbad, CA, USA). The detection of Iba-, neutrophil and caspase-3 stainings were done by using biotin-conjugated secondary antibody (1:200, Vector laboratories, Peterborough, UK) and nickel enhanced diaminobenzidine to visualize the stained cells.

GFAP, Iba-1 and caspase-3 immunoreactivities were quantified from the peri-ischemic cortical area immediately adjacent to the lesion border or the healthy contralateral hemisphere. The area was imaged using 10x magnification on an AX70 microscope (Olympus Corporation, Tokyo, Japan) attached with a digital camera (Color View 12 or F-View; Soft Imaging System, Münster, Germany) and running AnalySis Software (Soft Imaging System, Münster, Germany). Neutrophils were imaged at the lesion core, the site of infiltration, with 10 x magnification. The immunoreactivities were quantified blinded to the study groups using ImagePro Plus Software (Media Cybernetics, Rockville, MD, USA) and presented as relative immunoreactive area.

*In situ hybridization*

The localization of miR-127 in the ischemic brain was evaluated by in situ -hybridization. The sections were incubated with antigen retrieval in 0,01M sodium citrate buffer (pH 6,4) at 90C for 40 minutes followed by incubation of the slides in methylimidazole solution (0,13 M 1–methylimidazole, 300 mM NaCl in DEPC-treated water, pH 8,0) for 10 minutes and N-(3-Dimethylaminopropyl)-N′-ethylcarbodiimide hydrochloride (EDC) supplemented methylimidazole buffer for 1h. Slides were washed once with 0,2% (w/v) glycine in TBS (Tris-buffered saline, pH 7,4), twice with TBS, and incubated in the hybridization buffer (50% deionized formamide, 10mM Tris, 0,25% SDS, 200ug/ml yeast tRNA, 1 x Denhardt’s solution, 600mM NaCl, 1mM EDTA, 10% Dextran Sulphate in DEPC treated water) for 1h at 37 °C. 5’ digoxigenin labeled LNA probes were added to hybridization buffer and denatured at +65 °C, after which the probes were incubated on sections at 37 °C overnight.

The next day the stringency washes were performed at 44 °C, three times with 2 x SSC solution and twice with 0,2 x SSC solution, 20 minutes each. The sections were blocked with blocking buffer (1% bovine serum albumin (BSA), 3% normal goat serum (NGS) in PBS) for 1h at RT, after which they were incubated with anti-digoxigenin-POD (diluted 1:100 in blocking buffer) at 4°C overnight. On the following morning the slides were washed twice with TBS and FISH signal amplified with Cy5 Tsa-amplification kit (Perkin Elmer, Waltham, MA, USA) according to manufacturer’s instructions. After final washes the sections were mounted with Prolong Gold anti-fade reagent with DAPI (Invitrogen, Carlsbad, CA, USA), and they were allowed to dry at RT overnight, protected from light.

*Cytometric Bead Array*

Plasma cytokine concentrations were measured from the terminal plasma samples taken at the time of transcardial perfusion. 3.8 % tri-sodium citrate was used as an anticoagulant. The plasma was separated by centrifugation at 1500 x g for 6 min after which the plasma layer was centrifuged again at 12 000 x g to remove the platelets. Plasma was stored at - 70 °C until the analysis. The protein levels of mouse IL-2, IL-4 IL-6, IFN-γ, TNF, IL-17A and IL-10 were measured using mouse Th1/Th2/Th17 Cytokine Bead Array kit (BD Biosciences, San Jose, CA, USA) according to manufacturer’s instructions. The samples were run using FACS Calibur flow cytometer (BD Biosciences, San Jose, CA, USA) and the results were analyzed with FCAP Array 2.0.0 software (Soft Flow Hungary Ltd, Pecs, Hungary).

*Quantitative real-time reverse transcription-PCR (RT-PCR)*

The RNA was isolated from the dissected brain samples and miR-127 mimic- and mock-transfected N2a cells (see below) using miRVana-kit (Life Technologies, Carlsbad, CA, USA) according to the manufacturer’s instructions. The purity and the concentration of total RNA were measured using a Nanodrop 1000 spectrophotometer (Thermo Fisher Scientific, Waltham, MA, USA) and 500 ng of RNA was used for the cDNA synthesis done using random hexamer primers (Promega, Madison, WI, USA) and Maxima reverse transcriptase (Thermo Fisher Scientific, Waltham, MA, USA). The gene expression reactions were performed with StepOnePlus RT-PCR System (Life Technologies, Carlsbad, CA, USA) in accordance to the manufacturer’s instructions using Taqman reagents. The relative mRNA expression levels were measured by using specific assays-on-demand (Life Technologies, Carlsbad, CA, USA) target mixes for the genes of interest. The obtained results were normalized to GAPDH and β-actin (both validated to be stable in these experimental conditions, Thermo Fisher Scientific, Waltham, MA, USA) and are presented as fold change in the expression. For miRNA-qPCR the RT-reactions were carried out with 10ng of RNA with primers specific to microRNAs of interest. StepOnePlus -equipment was used for gene expression reactions. The amount of miRNA of interest in each sample was determined against a standard curve.

*Western blot*

Protein samples from the peri-ischemic area were collected in SDS sample buffer (0.0625 M TRIS-HCl, 2.3 % SDS, 5 % β-mercaptoethanol, 10 % glycerol, bromophenol blue). Ten micrograms of protein were loaded and run on 10 % SDS-PAGE gels. The proteins were transferred onto PVDF-hybond membranes with Trans-Blot Turbo® transfer system (BioRad, Hercules, CA, USA) for 20 minutes using 25V constant voltage. The membranes were blocked in 5 % milk in 0.2 % Tween20 in PBS (PBST) for 30 minutes. Primary antibody (PSMB5, Invitrogen, Carlsbad, CA, USA; PSMD3, Sigma-Aldrich, St. Louis, MO, USA; LDH (as a loading control), Santa Cruz Biotechnology Inc., Dallas, TX, USA) diluted 1:1000 in 5 % BSA in PBS and 0.02 % NaN_3_ was incubated on the membranes overnight at 4 °C. After washing with PBST, secondary antibody was incubated for 2 hours at RT diluted at 1:2000 in a blocking solution (anti-rabbit IgG HRP Conjugate, Bio-Rad, Hercules, CA, USA). After washing the membranes, the protein bands were visualized using a Clarity^TM^ Western ECL Substrate kit (Bio-Rad, Hercules, CA, USA). Images were taken with ChemiDoc^TM^ MP Imaging System (Bio-Rad, Hercules, CA, USA) using Image Lab software (Version 5.2.1, Bio-Rad, Hercules, CA, USA). Chicken β-actin Loading Control (1:1000 dilution, Abcam, Cambridge, UK) was used as another loading control. Fluorescent SpectraDye Goat-anti-chicken dye-650 (Advansta, Menlo Park, CA, USA) was a secondary antibody for this at 1:2500 dilution. Densitometric analysis were performed using ImageJ software (National Institutes of Health).

*Cortical neuron culture*

Primary cortical neuron cultures were prepared from mouse embryos of embryonic day 15. Cortices were dissected and tissue was dissociated with 0.0125% trypsin (for 15 minutes at +37 °C, Sigma-Aldrich, St. Louis, MO, USA). After trypsin inactivation and washing the cells were counted and plated on 48-well-plates (coated with poly-d-lysine, Sigma-Aldrich, St. Louis, MO, USA) at a density of 125 000 cells/well, in Neurobasal media supplemented with 2% B27, 500µM L-glutamine and 1% penicillin-streptomycin (all ThermoFisher Scientific, Waltham, MA, USA). On day 3 after plating the neurons were fed by changing 50% of media, and on day 5 the cultures were treated with glutamate for 24 hours (Sigma-Aldrich, St. Louis, MO, USA) at final concentration of 400 µM. Viability of neurons was assessed using MTT-measurement as described previously (van Meerloo, Kaspers, & Cloos, 2011). Alternatively, the cells were lysed and collected for extraction of proteins and RNA.

*BV2 cell culture*

Murine microglial BV2 cells were maintained in DMEM (Corning Cellgro, ThermoFisher Scientific, Waltham, MA, USA) supplemented with 10% FBS (low endotoxin, Hyclone, GE Life Sciences, Marlborough, MA, USA) and 1% penicillin/streptomycin (Invitrogen, Carlsbad, CA, USA).

*Neuro-2a cells and miRNA pulldown with biotinylated miRNA-mimics*

Mouse neuroblastoma (N2a) cell line was seeded at a density of 3.5 x 10^6^ cells/dish on 10 cm dishes in DMEM (1X) (Corning Cellgro, ThermoFisher Scientific, Waltham, MA, USA) supplemented with 10% FBS (low endotoxin, Hyclone, GE Life Sciences, Marlborough, MA, USA) and 1% penicillin/streptomycin (Invitrogen, Carlsbad, CA, USA). RNA pulldown was performed with modifications as previously described (Wani & Cloonan, 2014). Biotinylated mmu-miR-127-5p (miRCURY LNA microRNA mimics, Premium, Biotin, Exiqon A/S, Vedbaek, Denmark) and biotinylated control cel-miR-39-3p (miRCURY LNA microRNA mimics, Premium, Biotin, Exiqon A/S, Vedbaek, Denmark) in 50 nM concentration were used for the transfection. Transfections were performed with Viromer Blue (Lipocalyx GmbH, Halle, Germany) in Opti-MEM media (ThermoFisher Scientific, Waltham, MA, USA) for 4 h, after which the transfection medium was exchanged for complete DMEM (1X) for 20 h. Transfected N2a cells were exposed to 24 h of hypoxia (1 % O_2_, 5 % CO_2_ in pre-set Russkinn InvivO_2_ -chamber, Baker, Sanford, ME, USA), after which the cells were collected. The bound RNA was extracted from the magnetic Dynabeads MyOne Streptavidin C1 (ThermoFisher Scientific, Waltham, MA, USA) using mirVana miRNA Isolation Kit. RNA was reverse transcribed and cDNA templates were used for qPCR reactions with TaqMan gene expression assays. The results were normalized to control lysate values and then to fold changes calculated against control miRNA.

*Luciferase assay*

N2a cells were seeded at a density of 120 000 cells/well on 12-well-plate. The following day, cells were co-transfected with Dharmacon miRIDIAN mmu-miR-127-5p mimic (C-310923-01-0005, Horizon) or Dharmacon miRIDIAN microRNA Mimic Negative Control #1 (CN-001000-01-05, Horizon), and miTarget™ miRNA 3' UTR Target Clone for mouse Psmd3 mRNA plasmid (miT028449-MT06, GeneCopoeia, Rockville, MD, USA) or negative control vector plasmid (CmiT000001-MT06, GeneCopoeia) using Viromer Blue transfection reagent (Lipocalyx GmbH, Halle, Germany) with the following modifications: miRNA mimic:Viromer-complexes were formed using 1.12 ul of 50uM miRNA mimic stock (resulting in final concentration of 53.5 nM in the well) in 10ul buffer with 1ul of Viromer-reagent in 90 ul buffer. Plasmid:Viromer-complexes were formed using 2ug plasmid in 10ul buffer with 1 ul of Viromer-reagent in 90 ul buffer. Both complexes were formed by incubation of 15 min at RT, after which the Plasmid:Viromer-complexes were added to the miRNA mimic:Viromer-complexes and incubated for additional 5 min at RT. The resulting transfection complexes (200ul) were added to the cells. After 24h, cells were collected, washed once with PBS and used for luciferase assay. Luciferase assay was performed using Luc-Pair™ Duo-Luciferase Assay Kit 2.0 (GeneCopoeia) according to manufacturer’s instructions. Luminescence measurements were performed using CLARIOstar plate reader (BMG Labtech) with emission wavelength of 580 nm for Firefly Luciferase and 480 nm Renilla Luciferase. Results are presented as the ratio of RLU(Firefly):RLU(Renilla).

*Apoptosis- and proteasome activity assays*

N2a cells were seeded at a density of 125 000 cells/well on 6-well-plates, transfected with miR-127-5p mimic (mirVana miRNA mimic for mmu-miR-127-5p, ThermoFisher Scientific, Waltham, MA, USA) or mock and exposed to hypoxia as described above. The cells were collected immediately after hypoxia.

For apoptosis assay the apoptotic cells were labeled with APC Annexin Ready Flow -dye (1:12.5 dilution, Invitrogen, Carlsbad, CA, USA) in Annexin V Binding Buffer (10 mM HEPES, 150 mM NaCl, 2.5 mM CaCl_2_ in PBS, pH 7.4). 4',6-Diamidino-2-Phenylindole, Dilactate (DAPI, Invitrogen, Carlsbad, CA, USA) staining was added at 1:3300 dilution to label late apoptotic and necrotic cells. The samples were run with CytoFLEX S instrument (Beckman Coulter Life Sciences, Indianapolis, IN, USA), and the results were analysed with CytExpert software (version 2.3.0.84, Beckman Coulter Life Sciences, Indianapolis, IN, USA). The results of hypoxic miR-127 mimic transfected and mock transfected samples were normalized to normoxic mimic transfected and mock transfected samples, respectively.

For proteasome activity assay the cells were lysed without protease and phosphatase inhibitors, and protein content was measured by using Pierce^TM^ BCA Protein Assay Kit (ThermoFisher Scientific, Waltham, MA, USA). Equal amount of protein from each sample was loaded on 96-well-plate in duplicates, and measurement of chymotrypsin-like (Suc-LLVY-AMC) proteasome activity was performed with UBPBio Proteasome Activity Fluorometric Assay Kit II (Ubiquitin-Proteasome Biotechnologies LLC, Aurora, CO, USA) according to manufacturer’s instructions. Proteasomes were inhibited from the other duplicate of each sample with epoxomicin (MG132), and this iRFU-value was subtracted from the corresponding RFU (Relative Fluorescence Unit) value of another duplicate, obtained with Tecan Infinite M200 plate reader (Tecan Group Ltd., Männedorf, Switzerland) with exciting and emission filters at 360 and 460 nm, respectively.

*MicroRNA analysis of human samples*

Total RNA was isolated from around 20 mg brain tissue pieces using the Norgen Fatty Tissue RNA purification kit (Norgen Biotek Corp., Thorold, ON, Canada). Purified RNA was treated with DNase (DNA-free^TM^ DNA removal kit, Invitrogen, Carlsbad, CA, USA). Quantification and integrity of RNA samples were determined using Nanochips from the Bioanalyzer 2100 platform (Agilent Technologies Inc., Santa Clara, CA, USA) (RIN# 6.9 ± 0.8). MiRNAs contained in the RNA samples were labeled using the Flash Tag Biotin HSR RNA labeling kit (ThermoFisher Scientific, Waltham, MA, USA) before hybridization of the samples to GeneChip® miRNA 4.0 arrays (ThermoFisher Scientific, Waltham, MA, USA), according to manufacturer’s instructions. The images from the arrays were scanned to obtain intensity raw expression values.

All statistical analyses were performed using R language and common packages available at the Bioconductor Project. Quality control metrics supported a good quality in all arrays and subsequent analyses were initiated by preprocessing using the Robust Multi-Array Average (RMA) algorithm (Irizarry et al., 2003). After non-specific filtering, a linear model analysis with empirical Bayes modification for the variance estimates was applied (Smyth, 2004). Differentially expressed miRNAs were considered statistically significant when p-value <0.05. Logarithmic fold-change (logFC) was calculated by applying a base 2 logarithm to miRNA expression in each sample and then subtracting the average of all CL samples to the average of all IC samples (*logFC = log IC – log CL*).

*Library preparation and Next Generation sequencing*

MiRNA-seq was carried out by the MicroRNA and Small RNA Sequencing Service at Exiqon (Exiqon A/S, Vedbaek, Denmark). A total of 500 ng of total RNA from the peri-ischemic area was converted into microRNA NGS libraries using NEBNEXT library generation kit (New England Biolabs Inc., Ipswich, MA, USA) according to the manufacturer’s instructions. Each individual RNA sample had adaptors ligated to its 3’ and 5’ ends and converted into cDNA. The cDNA was pre-amplified with specific primers containing sample specific indexes. After 15 cycle pre-PCR the libraries were purified on QiaQuick columns and the insert efficiency evaluated by Bioanalyzer 2100 instrument on high sensitivity DNA chip (Agilent, Santa Clara, CA, USA). The microRNA cDNA libraries were size fractionated on a LabChip XT (Caliper, Perkin Elmer, Waltham, MA, USA) and a band representing adaptors and 15-40 bp insert excised using the manufacturer’s instructions. Samples were quantified using qPCR and concentration standards. Based on quality of the inserts and the concentration measurements the libraries were pooled in equimolar concentrations (all concentrations of libraries to be pooled are of the same concentration). The library pool(s) were finally quantified again with qPCR and optimal concentration of the library pool used to generate the clusters on the surface of a flowcell before sequencing using v3 sequencing methodology according to the manufacturer instructions (Illumina, San Diego, CA, USA). Samples were sequenced on the Illumina NextSeq 500 system. The system uses quality score binning enabling a more compact storage of raw sequences.

*GRO-seq assay*

The GRO-seq assay was performed as previously described (Kaikkonen et al., 2013). Cells were cultured as detailed above. BV2 cell samples modelling microglia included Kdo2-lipid A (KLA) treatment and control (DMSO) conditions with 1-hour treatment, one replicate each. Neuron samples included Glutamate treatment (400 µM) and control (H_2_O) conditions with 6 h treatment, in duplicates. Three million cells were collected from murine microglial BV2-cells and primary cultures of mouse neurons per sample. For nuclear extraction, PBS-washed cells were incubated in 10 ml of swelling buffer (10 mM Tris-HCl, 2 mM MgCl_2_, 3 mM CaCl_2_ and 2 U/ml SUPERase Inhibitor RNAse inhibitor (ThermoFisher Scientific, Waltham, MA, USA)) for 5 min on ice. Cells were pelleted for 10 min at 400 x g and resuspended in 500 µl of swelling buffer supplemented with 10% glycerol. Subsequently, 500 µl of swelling buffer supplemented with 10% glycerol and 1% Igepal (Sigma-Aldrich, St. Louis, MO, USA) was added under gentle vortexing. Nuclei were washed twice with lysis buffer (10 ml of swelling buffer supplemented with 0.5% Igepal and 10% glycerol), and once with 1 ml of freezing buffer (50 mM Tris-HCl pH 8.3, 40% glycerol, 5 mM MgCl_2_ and 0.1 mM EDTA), centrifuged at 900 x g for 6 min and suspended to a concentration of 1.5-5 million nuclei per 100 µl of freezing buffer, and stored -80°C until run-on reactions. For the run-on assay, the nuclear run-on reaction buffer (NRO-RB; 496 mM KCl, 16.5 mM Tris-HCl, 8.25 mM MgCl_2_ and 1.65% Sarkosyl (Sigma-Aldrich, St. Louis, MO, USA) was preheated to 30 °C, supplemented with 1.5 mM DTT, 750 mM ATP, 750 mM GTP, 4.5 mM CTP, 750 mM Br-UTP (Santa Cruz Biotechnology, Inc., Dallas, Tx, USA) and 33 µl of SUPERase Inhibitor. 50 µl of the supplemented NRO-RB was added to 100 µl of nuclei and incubated for 5 min at 30°C. GRO-seq libraries were subsequently prepared as initially described in Kaikkonen et al 2013 (Kaikkonen et al., 2013). Briefly, the NRO products were treated with DNAse I according to the manufacturer’s instructions (TURBO DNA-free Kit, ThermoFisher Scientific, Waltham, MA, USA), base-hydrolysed (RNA fragmentation reagent, ThermoFisher Scientific, Waltham, MA, USA), end-repaired and then immuno-purified using anti-Br-UTP beads (Santa Cruz Biotechnology, Inc., Dallas, Tx, USA). Subsequently, a poly-A tailing reaction (PolyA polymerase, New England Biolabs, Ipswich, MA, USA) was performed according to manufacturer’s instructions, followed by circularization and re-linearization. The cDNA templates were PCR amplified (Illumina barcoding) for 11–14 cycles and size selected to 220–350 bp length. The final libraries were quantified (Qubit dsDNA HS Assay Kit on a Qubit fluorometer, ThermoFisher Scientific, Waltham, MA, USA) and pooled for 50 bp single-end sequencing on Illumina Hi-Seq2000 (GeneCore, EMBL Heidelberg, Germany).

*GRO-seq data analysis*

Raw reads for available public GRO-seq data were downloaded from the Sequence Read Archive (SRA) database for mouse adipocytes (3T3-L1 cell line), primary embryonic stem cells (ESC), embryonic fibroblasts (E fibroblast), muscle (C2C12 cell line) and primary macrophages; the datasets are listed in Supplementary Table 1. The reads were trimmed using the HOMER v4.3 (http://homer.salk.edu/homer) (Heinz et al., 2010) software (homerTools trim) to remove A-stretches originating from the library preparation. From the resulting sequences, those shorter than 25 bp were discarded. The quality of raw sequencing reads was controlled using the FastQC tool (http://www.bioinformatics.babraham.ac.uk/projects/fastqc) (Krueger, Andrews, & Osborne, 2011) and bases with poor quality scores were trimmed (typically for read length of 50 requiring a minimum 97% of all bases in one read to have a minimum phred quality score of 10, otherwise adjusted based on read length) using the FastX toolkit (http://hannonlab.cshl.edu/fastx_toolkit/) available in the Galaxy platform (Blankenberg et al., 2010). Alignment to the murine mm9 reference genome version was preceded by removing reads mapping to rRNA regions (AbundantSequences as annotated by iGenomes) and blacklisted regions (unusual low or high mappability as defined by ENCODE, ribosomal and snoRNA loci from ENCODE and further manually curated for the mouse genome (coordinates file available upon request)), all processed with the Bowtie version bowtie-0.12.7 (Langmead, Trapnell, Pop, & Salzberg, 2009). Up to two mismatches and up to three locations were accepted per read and the best alignment was reported.

MiRNA loci from all cell types were detected using existing methodology for transcript identification from GRO-seq data (Bouvy-Liivrand et al., 2017). Briefly, initial *de novo* transcript detection was performed on pooled datasets for each cell type using HOMER v4.3. The identified primary transcripts were filtered with histone mark H3K4me3/H3K4me1 ratio and the transcription start site (TSS) coordinates were fixed following CAGE-seq (from the FANTOM5 consortium) nucleotide-specific adjustment. A count data matrix was generated using analyzeRepeats.pl (HOMER v4.3). For all miRNA loci with multiple detected TSSs, specific expression profiles were calculated using formulae *RPKMi = RPKMi – RPKM(i+1),* in which RPKM = reads per million kilobase, which allows for accurate quantitation of nascent RNA-seq reads.

*MiRNA-seq data and analysis*

MiRNA-seq data pre-processing and alignment was performed by the MicroRNA and Small RNA Sequencing Service at Exiqon (Exiqon A/S, Vedbaek, Denmark). Briefly, raw sequence reads quality controlled (Q-score cutoff > 30), adapters were trimmed using cutadapt v1.9.1 (Martin, 2011) and aligned end-to-end to the mm10 mouse genome using Bowtie2 v2.2.6 (Langmead & Salzberg, 2012) in the --sensitive mode, allowing no mismatches. The miRNA annotations were derived from miRBase v20. Samples with a minimum of 20% total reads mapping to the murine miRNome were kept. For differential expression analysis across the whole miRNA-seq dataset the R package limma (v3.34) (Law, Chen, Shi, & Smyth, 2014) was used. The original counts per million (CPM) matrix was filtered to contain at least 2 entries above CPM 1 per miRNA by each condition and a total of CPM 20 per miRNA across all conditions, leaving a total of 575 out of 1908 known miRNA species for differential expression analysis. The voom function was applied to transform the discrete raw count data into continuous values and estimate the mean-variance relationship across the dataset. All data points were divided into individual classes and replicates were considered in the analysis. The linear model framework in limma was used for statistical analysis. All miRNAs that passed the F-test adjusted P-value (Bonferroni-Hochberg) cutoff 0.05 were considered significantly differentially expressed.

Mature miRNA species that were detected as significant in the cohort Aged LPS (compared to Young LPS) were further filtered to only include miRNA species that were defined as neuron-expressed based on GRO-seq expression profile. This helps exclude miRNA species that can be taken up by neurons through exosomal transfer from other cell types in the mouse brain. Heat maps were generated using the R package gplots v3.0.1. Heatmaps are centered and normalized using the Z-score for visualization purposes. RPKM for GRO-seq data or CPM for miRNA-seq data were used as units for data normalization and plotting. Seq data were used as units for data normalization and plotting.

In order to reveal targets for miR-127, we took advantage of the TargetScan (Lewis, Shih, Jones-Rhoades, Bartel, & Burge, 2003) prediction tool and retrieved the predicted targets of mouse mir-127 (5p-arm) and human miR-127 (both 3p and 5p arms) (Fig. 4a). All the subsequent analysis were performed using R/Bioconductor (version 3.5.0). We used the package “STRINGdb” that provides an interface to the STRING protein-protein interaction database (Franceschini et al., 2013). Protein-protein networks (PPI) of the predicted targets were built taking into account only the interactions with the highest interaction score. Starting from the human and mouse PPI networks of the predicted targets, we applied the community detection algorithm *walktrap* (Pons & Latapy, 2006) to discover very connected “clusters” of genes. For each cluster, we did KEGG functional enrichment to retrieve significantly enriched pathways with false discovery rate (FDR) of 5%. Next, we used *overlap coefficient* (Vijaymeena & Kavitha, 2016) to compare the pairs of *Mus musculus* and *Homo sapiens* clusters and to find the best candidate pairs in terms of pathway similarity. For this analysis only clusters with more than 15 genes were taken into account. Among the top three couples we identified the cluster pair *Mus musculus* cluster 9 – *Homo sapiens* cluster 2 (Fig. 4b), which include pathways ”Ubiquitin mediated proteolysis” and ”Proteasome” (Fig. 4c,d). When exploring genes enriched within these pathways, proteasomes *PSMB5* and *Psmd3* (Fig. 4e,f) were discovered. In addition, analyzing the modules of clusters 9 and 2 and focusing on the more connected ones, we discovered that both in human and mouse clusters, the genes *Psmd4, Btrc, Skp2, Cdc27* and *Psmb11,* all involved in ubiquitin-proteasome system in one way or another, are connected with proteasome genes *PSMB5* and *Psmd3* (Fig. 4e,f). Since proteasomes are known to be connected with inflammation, apoptotic processes and caspase activity (Chestnut et al., 2011; Patel et al., 2013; Shi et al., 2015; Shohat, Ben-Meir, & Lavi, 2012), they were chosen to further analysis.

**References**

Bargiotas, P., Krenz, A., Monyer, H., & Schwaninger, M. (2012). Functional outcome of pannexin-deficient mice after cerebral ischemia. *Channels (Austin, Tex.)*, *6*(6), 453–456. https://doi.org/10.4161/chan.22315

Blankenberg, D., Gordon, A., Von Kuster, G., Coraor, N., Taylor, J., Nekrutenko, A., & Galaxy Team, the G. (2010). Manipulation of FASTQ data with Galaxy. *Bioinformatics (Oxford, England)*, *26*(14), 1783–1785. https://doi.org/10.1093/bioinformatics/btq281

Bouet, V., Boulouard, M., Toutain, J., Divoux, D., Bernaudin, M., Schumann-Bard, P., & Freret, T. (2009). The adhesive removal test: a sensitive method to assess sensorimotor deficits in mice. *Nature Protocols*, *4*(10), 1560–1564. https://doi.org/10.1038/nprot.2009.125

Bouvy-Liivrand, M., Hernández de Sande, A., Pölönen, P., Mehtonen, J., Vuorenmaa, T., Niskanen, H., … Heinäniemi, M. (2017). Analysis of primary microRNA loci from nascent transcriptomes reveals regulatory domains governed by chromatin architecture. *Nucleic Acids Research*, *45*(17), 9837–9849. https://doi.org/10.1093/nar/gkx680

Chestnut, B. A., Chang, Q., Price, A., Lesuisse, C., Wong, M., & Martin, L. J. (2011). Epigenetic Regulation of Motor Neuron Cell Death through DNA Methylation. *The Journal of Neuroscience*, *31*(46), 16619–16636. https://doi.org/10.1523/JNEUROSCI.1639-11.2011

Dhungana, H., Rolova, T., Savchenko, E., Wojciechowski, S., Savolainen, K., Ruotsalainen, A.-K., … Malm, T. (2013). Western-type diet modulates inflammatory responses and impairs functional outcome following permanent middle cerebral artery occlusion in aged mice expressing the human apolipoprotein E4 allele. *Journal of Neuroinflammation*, *10*, 102. https://doi.org/10.1186/1742-2094-10-102

Franceschini, A., Szklarczyk, D., Frankild, S., Kuhn, M., Simonovic, M., Roth, A., … Jensen, L. J. (2013). STRING v9.1: protein-protein interaction networks, with increased coverage and integration. *Nucleic Acids Research*, *41*(Database issue), D808-15. https://doi.org/10.1093/nar/gks1094

Heinz, S., Benner, C., Spann, N., Bertolino, E., Lin, Y. C., Laslo, P., … Glass, C. K. (2010). Simple combinations of lineage-determining transcription factors prime cis-regulatory elements required for macrophage and B cell identities. *Molecular Cell*, *38*(4), 576–589. https://doi.org/10.1016/j.molcel.2010.05.004

Irizarry, R. A., Hobbs, B., Collin, F., Beazer-Barclay, Y. D., Antonellis, K. J., Scherf, U., & Speed, T. P. (2003). Exploration, normalization, and summaries of high density oligonucleotide array probe level data. *Biostatistics*, *4*(2), 249–264. https://doi.org/10.1093/biostatistics/4.2.249

Kaikkonen, M. U., Spann, N. J., Heinz, S., Romanoski, C. E., Allison, K. A., Stender, J. D., … Glass, C. K. (2013). Remodeling of the enhancer landscape during macrophage activation is coupled to enhancer transcription. *Molecular Cell*, *51*(3), 310–325. https://doi.org/10.1016/j.molcel.2013.07.010

Krueger, F., Andrews, S. R., & Osborne, C. S. (2011). Large scale loss of data in low-diversity illumina sequencing libraries can be recovered by deferred cluster calling. *PloS One*, *6*(1), e16607. https://doi.org/10.1371/journal.pone.0016607

Langmead, B., & Salzberg, S. L. (2012). Fast gapped-read alignment with Bowtie 2. *Nature Methods*, *9*(4), 357–359. https://doi.org/10.1038/nmeth.1923

Langmead, B., Trapnell, C., Pop, M., & Salzberg, S. L. (2009). Ultrafast and memory-efficient alignment of short DNA sequences to the human genome. *Genome Biology*, *10*(3), R25. https://doi.org/10.1186/gb-2009-10-3-r25

Law, C. W., Chen, Y., Shi, W., & Smyth, G. K. (2014). voom: Precision weights unlock linear model analysis tools for RNA-seq read counts. *Genome Biology*, *15*(2), R29. https://doi.org/10.1186/gb-2014-15-2-r29

Lewis, B. P., Shih, I., Jones-Rhoades, M. W., Bartel, D. P., & Burge, C. B. (2003). Prediction of mammalian microRNA targets. *Cell*, *115*(7), 787–798. https://doi.org/10.1016/S0092-8674(03)01018-3

Martin, M. (2011). Cutadapt removes adapter sequences from high-throughput sequencing reads. *EMBnet.Journal*, *17*(1), 10. https://doi.org/10.14806/ej.17.1.200

Patel, V. N., Gokulrangan, G., Chowdhury, S. A., Chen, Y., Sloan, A. E., Koyutürk, M., … Chance, M. R. (2013). Network signatures of survival in glioblastoma multiforme. *PLoS Computational Biology*, *9*(9), e1003237. https://doi.org/10.1371/journal.pcbi.1003237

Pons, P., & Latapy, M. (2006). *Journal of Graph Algorithms and Applications Computing Communities in Large Networks Using Random Walks* (Vol. 10). Retrieved from http://jgaa.info/volhttp://www.liafa.jussieu.fr/

Shi, J., Liu, X., Xu, C., Ge, J., Ren, J., Wang, J., … Lu, H. (2015). Up-regulation of PSMB4 is associated with neuronal apoptosis after neuroinflammation induced by lipopolysaccharide. *Journal of Molecular Histology*, *46*(6), 457–466. https://doi.org/10.1007/s10735-015-9637-0

Shohat, M., Ben-Meir, D., & Lavi, S. (2012). Protein Phosphatase Magnesium Dependent 1A (PPM1A) Plays a Role in the Differentiation and Survival Processes of Nerve Cells. *PLoS ONE*, *7*(2), e32438. https://doi.org/10.1371/journal.pone.0032438

Shuaib, A., Xu Wang, C., Yang, T., & Noor, R. (2002). Effects of nonpeptide V(1) vasopressin receptor antagonist SR-49059 on infarction volume and recovery of function in a focal embolic stroke model. *Stroke*, *33*(12), 3033–3037. Retrieved from http://www.ncbi.nlm.nih.gov/pubmed/12468809

Smyth, G. K. (2004). Linear Models and Empirical Bayes Methods for Assessing Differential Expression in Microarray Experiments. *Statistical Applications in Genetics and Molecular Biology*, *3*(1), 1–25. https://doi.org/10.2202/1544-6115.1027

van Meerloo, J., Kaspers, G. J. L., & Cloos, J. (2011). Cell Sensitivity Assays: The MTT Assay. In *Methods in molecular biology (Clifton, N.J.)* (Vol. 731, pp. 237–245). https://doi.org/10.1007/978-1-61779-080-5_20

Vijaymeena, M. K., & Kavitha, K. (2016). A SURVEY ON SIMILARITY MEASURES IN TEXT MINING. *Machine Learning and Applications: An International Journal (MLAIJ)*, *3*(1). https://doi.org/10.5121/mlaij.2016.3103

Wani, S., & Cloonan, N. (2014). Profiling direct mRNA-microRNA interactions using synthetic biotinylated microRNA-duplexes. *BioRxiv*, 005439. https://doi.org/10.1101/005439

**Supplementary Table 1.**

| **GSE id** | **GSM id** | **Cell Type** | **Treatment** | **Collection Time** | **Replicate** | **Genome Version** | **Tissue source** | **Specific type** |
| --- | --- | --- | --- | --- | --- | --- | --- | --- |
| GSE45517 | GSM1106091 | MEF | notx | NA | 1 | mm9 | embryo | fibroblast |
| GSE45517 | GSM1106092 | MEF | Srsf1KO | NA | 1 | mm9 | embryo | fibroblast |
| GSE45517 | GSM1106093 | MEF | notx | NA | 2 | mm9 | embryo | fibroblast |
| GSE45517 | GSM1106094 | MEF | Srsf2KO | NA | 1 | mm9 | embryo | fibroblast |
| GSE23622 | GSM579129 | macrophage | notx | 1h | 1 | mm9 | peritoneum | peritoneal macrophage |
| GSE23622 | GSM579130 | macrophage | KDO | 1h | 1 | mm9 | peritoneum | peritoneal macrophage |
| GSE48759 | GSM1183906 | macrophage | notx | 1h | 1 | mm9 | peritoneum | peritoneal macrophage |
| GSE48759 | GSM1183909 | macrophage | notx | 24h | 2 | mm9 | peritoneum | peritoneal macrophage |
| GSE48759 | GSM1183910 | macrophage | KLA | 1h | 1 | mm9 | peritoneum | peritoneal macrophage |
| GSE48759 | GSM1183922 | macrophage | notx | 1h | 3 | mm9 | peritoneum | peritoneal macrophage |
| GSE48759 | GSM1183922 | macrophage | notx | 1h | 4 | mm9 | peritoneum | peritoneal macrophage |
| GSE48759 | GSM1183923 | macrophage | KLA | 1h | 2 | mm9 | peritoneum | peritoneal macrophage |
| GSE48759 | GSM1183925 | macrophage | KLA | 6h | 1 | mm9 | peritoneum | peritoneal macrophage |
| GSE48759 | GSM1183926 | macrophage | KLA | 1h | 3 | mm9 | peritoneum | peritoneal macrophage |
| GSE48759 | GSM1183926 | macrophage | KLA | 1h | 4 | mm9 | peritoneum | peritoneal macrophage |
| GSE48759 | GSM1183927 | macrophage | InhDMSO | 2h | 1 | mm9 | peritoneum | peritoneal macrophage |
| GSE48759 | GSM1183928 | macrophage | InhFlavo | 2h | 1 | mm9 | peritoneum | peritoneal macrophage |
| GSE48759 | GSM1183929 | macrophage | InhIBET | 2h | 1 | mm9 | peritoneum | peritoneal macrophage |
| GSE48759 | GSM1183930 | macrophage | InhDMSOKLA | 2h1h | 1 | mm9 | peritoneum | peritoneal macrophage |
| GSE48759 | GSM1183931 | macrophage | InhFlavoKLA | 2h1h | 1 | mm9 | peritoneum | peritoneal macrophage |
| GSE48759 | GSM1183932 | macrophage | InhIBETKLA | 2h1h | 1 | mm9 | peritoneum | peritoneal macrophage |
| GSE48759 | GSM1183933 | macrophage | InhFlavoKLA | 2h1h | 1 | mm9 | peritoneum | peritoneal macrophage |
| GSE48759 | GSM1183934 | macrophage | InhDMSo | 2h | 2 | mm9 | peritoneum | peritoneal macrophage |
| GSE48759 | GSM1183935 | macrophage | InhDMSOKLA | 2h1h | 1 | mm9 | peritoneum | peritoneal macrophage |
| GSE48759 | GSM1183936 | macrophage | InhActDKLA | 2h1h | 1 | mm9 | peritoneum | peritoneal macrophage |
| GSE48759 | GSM1183937 | macrophage | InhTripKLA | 2h1h | 1 | mm9 | peritoneum | peritoneal macrophage |
| GSE48759 | GSM1183938 | macrophage | InhAmantKLA | 5h1h | 1 | mm9 | peritoneum | peritoneal macrophage |
| GSE48895 | GSM1186440 | ESC | notx | NA | 1 | mm9 | blastocyst | embryonic stem cell |
| GSE48895 | GSM1186441 | ESC | notx | NA | 2 | mm9 | blastocyst | embryonic stem cell |
| GSE48895 | GSM1186442 | ESC | FP | 2min | 1 | mm9 | blastocyst | embryonic stem cell |
| GSE48895 | GSM1186443 | ESC | FP | 2min | 2 | mm9 | blastocyst | embryonic stem cell |
| GSE48895 | GSM1186444 | ESC | FP | 5min | 1 | mm9 | blastocyst | embryonic stem cell |
| GSE48895 | GSM1186445 | ESC | FP | 5min | 2 | mm9 | blastocyst | embryonic stem cell |
| GSE48895 | GSM1186448 | ESC | FP | 25min | 1 | mm9 | blastocyst | embryonic stem cell |
| GSE48895 | GSM1186449 | ESC | FP | 25min | 2 | mm9 | blastocyst | embryonic stem cell |
| GSE48895 | GSM1186450 | ESC | FP | 50min | 1 | mm9 | blastocyst | embryonic stem cell |
| GSE48895 | GSM1186451 | ESC | FP | 50min | 2 | mm9 | blastocyst | embryonic stem cell |
| GSE48895 | GSM1186452 | ESC | dmso | 50min | 1 | mm9 | blastocyst | embryonic stem cell |
| GSE48895 | GSM1186453 | ESC | dmso | 50min | 2 | mm9 | blastocyst | embryonic stem cell |
| GSE48895 | GSM1186454 | ESC | TRP | 12.5min | 1 | mm9 | blastocyst | embryonic stem cell |
| GSE48895 | GSM1186455 | ESC | TRP | 12.5min | 2 | mm9 | blastocyst | embryonic stem cell |
| GSE48895 | GSM1186456 | ESC | TRP | 25min | 2 | mm9 | blastocyst | embryonic stem cell |
| GSE48895 | GSM1186457 | ESC | TRP | 50min | 1 | mm9 | blastocyst | embryonic stem cell |
| GSE48895 | GSM1186458 | ESC | TRP | 50min | 2 | mm9 | blastocyst | embryonic stem cell |
| GSE56747 | GSM1367990 | 3T3L1 | notx | NA | 1 | mm9 | embryo | preadipocyte |
| GSE56747 | GSM1367991 | 3T3L1 | Rosi | 10min | 1 | mm9 | embryo | preadipocyte |
| GSE56747 | GSM1367992 | 3T3L1 | Rosi | 30min | 1 | mm9 | embryo | preadipocyte |
| GSE56747 | GSM1367993 | 3T3L1 | Rosi | 1h | 1 | mm9 | embryo | preadipocyte |
| GSE56747 | GSM1367994 | 3T3L1 | Rosi | 3h | 1 | mm9 | embryo | preadipocyte |
| GSE56747 | GSM1367995 | 3T3L1 | notx | NA | 2 | mm9 | embryo | preadipocyte |
| GSE56747 | GSM1367996 | 3T3L1 | Rosi | 10min | 2 | mm9 | embryo | preadipocyte |
| GSE56747 | GSM1367997 | 3T3L1 | Rosi | 30min | 2 | mm9 | embryo | preadipocyte |
| GSE56747 | GSM1367998 | 3T3L1 | Rosi | 1h | 2 | mm9 | embryo | preadipocyte |
| GSE56747 | GSM1367999 | 3T3L1 | Rosi | 3h | 2 | mm9 | embryo | preadipocyte |
| GSE62296 | GSM1524922 | MEF | notx | NA | 1 | mm9 | embryo | fibroblast |
| GSE66703 | GSM1629980 | neuron | notx | 1h | 1 | mm9 | brain | primary neuron |
| GSE66703 | GSM1629981 | neuron | Reelin | 1h | 1 | mm9 | brain | primary neuron |
| GSE23622 | GSM579129 | macrophage | notx | NA | 1 | mm9 | peritoneum | peritoneal macrophage |
| GSE23622 | GSM579130 | macrophage | KDO | 1h | 1 | mm9 | peritoneum | peritoneal macrophage |
| GSE26512 | GSM651870 | C2C12 | notx | NA | 1 | mm9 | muscle | muscle cell |
| GSE27037 | GSM665994 | ESC | notx | NA | 1 | mm9 | blastocyst | embryonic stem cell |
| GSE27037 | GSM665995 | ESC | notx | NA | 2 | mm9 | blastocyst | embryonic stem cell |
| GSE27037 | GSM665996 | ESC | notx | NA | 3 | mm9 | blastocyst | embryonic stem cell |
| GSE27037 | GSM665997 | MEF | notx | NA | 1 | mm9 | embryo | fibroblast |
| GSE27037 | GSM665998 | MEF | notx | NA | 2 | mm9 | embryo | fibroblast |
| GSE60857 | GSM1490114 | CD14 | notx | 0 | 1 | UCSC | mm9 | bone marrow macrophage |
| GSE60857 | GSM1490115 | CD14 | notx | 0 | 2 | UCSC | mm9 | bone marrow macrophage |
| GSE60857 | GSM1490116 | CD14 | LPS | 20min | 1 | UCSC | mm9 | bone marrow macrophage |
| GSE60857 | GSM1490117 | CD14 | LPS | 20min | 2 | UCSC | mm9 | bone marrow macrophage |
| GSE60857 | GSM1490118 | CD14 | LPS | 60min | 1 | UCSC | mm9 | bone marrow macrophage |
| GSE60857 | GSM1490119 | CD14 | LPS | 60min | 2 | UCSC | mm9 | bone marrow macrophage |
| GSE60857 | GSM1490120 | CD14 | LPS | 180min | 1 | UCSC | mm9 | bone marrow macrophage |
| GSE60857 | GSM1490121 | CD14 | LPS | 180min | 2 | UCSC | mm9 | bone marrow macrophage |
| GSEXXXXX (this study) | ADD | microglia | notx | 1h | 1 | mm9 | brain | primary microglia |
| GSEXXXXX (this study) | ADD | microglia | KLA | 1h | 1 | mm9 | brain | primary microglia |
| GSEXXXXX (this study) | ADD | neuron | notx | 1h | 1 | mm9 | brain | primary neuron |
| GSEXXXXX (this study) | ADD | neuron | notx | 1h | 2 | mm9 | brain | primary neuron |
| GSEXXXXX (this study) | ADD | neuron | Glutamate | 1h | 1 | mm9 | brain | primary neuron |
| GSEXXXXX (this study) | ADD | neuron | Glutamate | 1h | 2 | mm9 | brain | primary neuron |

**Supplementary Table 2.**

| **microRNA** | **logFC** | **AveExpr** | **t** | **P.Value** | **adj.P.Val** | **B** |
| --- | --- | --- | --- | --- | --- | --- |
| mmu-miR-3059-5p | -0,7001 | 5,6385 | -5,0764 | 0,0000 | 0,0125 | 2,1072 |
| mmu-miR-146a-5p | 0,8755 | 8,3926 | 4,9052 | 0,0001 | 0,0125 | 1,7808 |
| mmu-miR-203-3p | 0,6859 | 8,4230 | 4,8858 | 0,0001 | 0,0125 | 1,7381 |
| mmu-miR-152-3p | 0,4512 | 8,7205 | 4,8029 | 0,0001 | 0,0125 | 1,5550 |
| mmu-miR-341-3p | -0,6664 | 8,7974 | -4,6606 | 0,0001 | 0,0141 | 1,2381 |
| mmu-miR-127-5p | -0,5451 | 10,4534 | -4,5300 | 0,0002 | 0,0148 | 0,9421 |
| mmu-miR-381-3p | -1,0014 | 12,1302 | -4,4600 | 0,0002 | 0,0148 | 0,7755 |
| mmu-miR-540-5p | -1,1463 | 2,8944 | -4,4458 | 0,0002 | 0,0148 | 0,5669 |
| mmu-miR-344f-3p | -0,9230 | 3,8719 | -4,2929 | 0,0003 | 0,0191 | 0,3819 |
| mmu-miR-1983 | -0,4359 | 6,1828 | -4,2239 | 0,0004 | 0,0192 | 0,2713 |
| mmu-miR-132-5p | -0,5992 | 11,1871 | -4,2092 | 0,0004 | 0,0192 | 0,2139 |
| mmu-miR-212-5p | -0,8957 | 10,4049 | -4,1428 | 0,0004 | 0,0207 | 0,0699 |
| mmu-miR-335-5p | 0,9954 | 8,2363 | 4,0841 | 0,0005 | 0,0220 | -0,0463 |
| mmu-miR-879-5p | -0,5195 | 5,0520 | -4,0312 | 0,0006 | 0,0233 | -0,1507 |
| mmu-miR-296-3p | -0,6847 | 3,5510 | -3,9538 | 0,0007 | 0,0252 | -0,3411 |
| mmu-miR-351-5p | -0,5062 | 5,5377 | -3,9425 | 0,0007 | 0,0252 | -0,3411 |
| mmu-miR-3081-3p | -0,7150 | 4,0830 | -3,8107 | 0,0010 | 0,0327 | -0,6281 |
| mmu-miR-26b-5p | 0,4925 | 10,2538 | 3,6333 | 0,0015 | 0,0474 | -1,0673 |
| mmu-miR-155-5p | 1,0508 | 3,2258 | 3,6070 | 0,0016 | 0,0478 | -1,0616 |
| mmu-miR-31-5p | 1,0132 | 6,3972 | 3,5756 | 0,0017 | 0,0490 | -1,1414 |
| mmu-miR-673-3p | -0,5830 | 5,0006 | -3,5344 | 0,0019 | 0,0492 | -1,2126 |
| mmu-miR-488-5p | 1,0109 | 2,0468 | 3,5335 | 0,0019 | 0,0492 | -1,2985 |
| mmu-miR-25-3p | 0,3458 | 7,9562 | 3,5037 | 0,0020 | 0,0506 | -1,3235 |
| mmu-miR-299a-3p | -0,4461 | 5,9145 | -3,4720 | 0,0022 | 0,0523 | -1,3572 |
| mmu-miR-300-3p | -0,3299 | 11,7800 | -3,3823 | 0,0027 | 0,0604 | -1,6391 |
| mmu-miR-296-5p | -1,1778 | 3,2719 | -3,3695 | 0,0028 | 0,0604 | -1,5442 |
| mmu-miR-412-5p | -0,5125 | 5,6468 | -3,3618 | 0,0028 | 0,0604 | -1,5880 |
| mmu-miR-376b-5p | -0,4387 | 7,8858 | -3,3242 | 0,0031 | 0,0637 | -1,7098 |
| mmu-miR-384-5p | 0,5163 | 8,3626 | 3,2954 | 0,0033 | 0,0659 | -1,7801 |
| mmu-let-7i-3p | -0,6402 | 5,6795 | -3,2757 | 0,0035 | 0,0667 | -1,7655 |
| mmu-miR-344-3p | 0,3579 | 11,2725 | 3,2466 | 0,0037 | 0,0691 | -1,9273 |
| mmu-let-7a-5p | 0,3532 | 13,2747 | 3,2111 | 0,0041 | 0,0709 | -2,0339 |
| mmu-let-7f-1-3p | -0,8700 | 3,9670 | -3,2027 | 0,0041 | 0,0709 | -1,8810 |
| mmu-miR-376b-3p | 0,6961 | 8,1100 | 3,1957 | 0,0042 | 0,0709 | -1,9866 |
| mmu-miR-103-3p | 0,7114 | 11,8712 | 3,1841 | 0,0043 | 0,0709 | -2,0698 |
| mmu-let-7e-5p | 0,3155 | 10,9764 | 3,1516 | 0,0047 | 0,0719 | -2,1259 |
| mmu-miR-1291 | 1,3649 | 3,9796 | 3,1453 | 0,0047 | 0,0719 | -1,9951 |
| mmu-miR-218-5p | -0,2958 | 13,5594 | -3,1432 | 0,0048 | 0,0719 | -2,1847 |
| mmu-miR-185-5p | 0,7046 | 10,8782 | 3,0921 | 0,0054 | 0,0790 | -2,2494 |
| mmu-miR-132-3p | -0,3795 | 11,9130 | -3,0070 | 0,0065 | 0,0938 | -2,4477 |
| mmu-miR-181c-3p | -0,3177 | 6,5897 | -2,9762 | 0,0070 | 0,0954 | -2,4127 |
| mmu-miR-144-5p | 2,4718 | 0,0582 | 2,9704 | 0,0071 | 0,0954 | -2,5156 |
| mmu-miR-199b-3p | 0,5039 | 5,4800 | 2,9684 | 0,0071 | 0,0954 | -2,3950 |
| mmu-miR-135a-2-3p | 0,6005 | 3,9253 | 2,9301 | 0,0078 | 0,1018 | -2,4267 |
| mmu-miR-181c-5p | -0,3235 | 7,6841 | -2,9138 | 0,0081 | 0,1024 | -2,5678 |
| mmu-miR-22-3p | -0,3739 | 13,0065 | -2,9082 | 0,0082 | 0,1024 | -2,6725 |
| mmu-let-7c-5p | 0,3448 | 13,9547 | 2,8953 | 0,0084 | 0,1032 | -2,7157 |
| mmu-miR-22-5p | -0,3736 | 7,0390 | -2,8695 | 0,0090 | 0,1072 | -2,6420 |
| mmu-miR-184-3p | -0,9789 | 5,4433 | -2,8565 | 0,0092 | 0,1082 | -2,6217 |
| mmu-miR-370-3p | -0,7505 | 11,8231 | -2,8361 | 0,0097 | 0,1111 | -2,8023 |
| mmu-miR-409-3p | -0,6388 | 9,4415 | -2,8238 | 0,0099 | 0,1114 | -2,7867 |
| mmu-miR-669p-5p | -0,6751 | 1,6619 | -2,8177 | 0,0101 | 0,1114 | -2,6121 |
| mmu-let-7d-5p | 0,4310 | 11,9500 | 2,8026 | 0,0104 | 0,1114 | -2,8719 |
| mmu-miR-376c-5p | -0,6632 | 1,4215 | -2,7967 | 0,0106 | 0,1114 | -2,6603 |
| mmu-miR-467a-5p | -0,6008 | 6,0869 | -2,7930 | 0,0107 | 0,1114 | -2,7704 |
| mmu-miR-344d-3-5p | 0,3721 | 4,3415 | 2,7422 | 0,0119 | 0,1219 | -2,8060 |
| mmu-miR-7689-3p | -0,3787 | 5,1636 | -2,7371 | 0,0121 | 0,1219 | -2,8458 |
| mmu-miR-6516-5p | 0,6149 | 3,2741 | 2,7060 | 0,0130 | 0,1283 | -2,8266 |
| mmu-miR-455-3p | 0,6497 | 5,2691 | 2,6941 | 0,0133 | 0,1283 | -2,9316 |
| mmu-miR-674-3p | -0,7675 | 9,3750 | -2,6904 | 0,0134 | 0,1283 | -3,0548 |
| mmu-let-7b-3p | -0,8184 | 5,4333 | -2,6792 | 0,0138 | 0,1283 | -2,9682 |
| mmu-miR-210-3p | 0,4979 | 6,2044 | 2,6644 | 0,0142 | 0,1283 | -3,0230 |
| mmu-miR-182-5p | 1,3708 | 7,9183 | 2,6620 | 0,0143 | 0,1283 | -3,0707 |
| mmu-miR-181a-1-3p | -0,3038 | 8,4765 | -2,6502 | 0,0147 | 0,1283 | -3,1162 |
| mmu-miR-486-5p | 0,4686 | 5,8881 | 2,6399 | 0,0150 | 0,1283 | -3,0644 |
| mmu-miR-33-3p | 1,1254 | 0,9622 | 2,6302 | 0,0153 | 0,1283 | -2,9711 |
| mmu-miR-129-5p | -0,6321 | 12,2531 | -2,6272 | 0,0154 | 0,1283 | -3,2333 |
| mmu-miR-1264-3p | 0,9141 | 4,5839 | 2,6210 | 0,0157 | 0,1283 | -3,0410 |
| mmu-miR-129-1-3p | -0,3617 | 8,4549 | -2,6182 | 0,0158 | 0,1283 | -3,1787 |
| mmu-let-7j | 0,2582 | 10,0447 | 2,6160 | 0,0158 | 0,1283 | -3,2132 |
| mmu-miR-1306-5p | 0,7226 | 2,5100 | 2,6115 | 0,0160 | 0,1283 | -2,9711 |
| mmu-miR-873a-5p | -0,3129 | 8,2219 | -2,6096 | 0,0161 | 0,1283 | -3,1921 |
| mmu-miR-34b-3p | -0,5391 | 3,6897 | -2,6012 | 0,0164 | 0,1289 | -3,0399 |
| mmu-miR-192-5p | -0,5319 | 9,0640 | -2,5901 | 0,0168 | 0,1303 | -3,2469 |
| mmu-miR-361-3p | -0,3386 | 9,9104 | -2,5759 | 0,0173 | 0,1314 | -3,2888 |
| mmu-let-7g-5p | 0,3105 | 15,0210 | 2,5710 | 0,0175 | 0,1314 | -3,3948 |
| mmu-miR-320-3p | 0,3195 | 8,4272 | 2,5684 | 0,0176 | 0,1314 | -3,2757 |
| mmu-miR-191-5p | 0,4013 | 11,9577 | 2,5316 | 0,0191 | 0,1396 | -3,4129 |
| mmu-miR-3107-5p | 0,3630 | 5,9885 | 2,5245 | 0,0194 | 0,1396 | -3,2893 |
| mmu-miR-450a-5p | -0,4405 | 4,5270 | -2,5194 | 0,0196 | 0,1396 | -3,2308 |
| mmu-miR-503-5p | -0,5672 | 2,2641 | -2,5176 | 0,0197 | 0,1396 | -3,1315 |
| mmu-let-7a-1-3p | -0,5591 | 7,2662 | -2,5087 | 0,0201 | 0,1401 | -3,3539 |
| mmu-miR-451a | 1,7869 | 3,6184 | 2,5009 | 0,0204 | 0,1401 | -3,2069 |
| mmu-miR-3473b | 1,3148 | 2,1891 | 2,4994 | 0,0205 | 0,1401 | -3,1562 |
| mmu-miR-200c-3p | 1,2375 | 5,2979 | 2,4812 | 0,0213 | 0,1423 | -3,3204 |
| mmu-miR-223-3p | 1,1833 | 2,3970 | 2,4769 | 0,0215 | 0,1423 | -3,1935 |
| mmu-miR-467b-5p | -0,7269 | 1,7068 | -2,4611 | 0,0222 | 0,1423 | -3,2145 |
| mmu-miR-135b-5p | -0,4451 | 7,4373 | -2,4578 | 0,0224 | 0,1423 | -3,4588 |
| mmu-miR-335-3p | -0,2985 | 9,0341 | -2,4562 | 0,0225 | 0,1423 | -3,5024 |
| mmu-miR-23b-3p | 0,5440 | 9,1983 | 2,4555 | 0,0225 | 0,1423 | -3,5071 |
| mmu-miR-99b-3p | -0,4045 | 6,9985 | -2,4392 | 0,0233 | 0,1423 | -3,4846 |
| mmu-let-7c-2-3p | -0,5538 | 7,2607 | -2,4383 | 0,0234 | 0,1423 | -3,4874 |
| mmu-miR-690 | 0,8090 | 4,3158 | 2,4340 | 0,0236 | 0,1423 | -3,3859 |
| mmu-miR-485-3p | -0,3955 | 6,7566 | -2,4301 | 0,0238 | 0,1423 | -3,4913 |
| mmu-miR-700-3p | 0,4420 | 4,9189 | 2,4291 | 0,0238 | 0,1423 | -3,4177 |
| mmu-miR-376c-3p | -0,3629 | 5,7494 | -2,4284 | 0,0239 | 0,1423 | -3,4574 |
| mmu-miR-99b-5p | -0,5092 | 12,1647 | -2,4259 | 0,0240 | 0,1423 | -3,6224 |
| mmu-miR-199a-3p | 0,5072 | 5,8798 | 2,4137 | 0,0246 | 0,1444 | -3,4854 |
| mmu-miR-539-5p | -0,3709 | 3,5185 | -2,4090 | 0,0249 | 0,1444 | -3,3886 |
| mmu-miR-582-3p | -0,4158 | 7,7966 | -2,3996 | 0,0254 | 0,1444 | -3,5790 |
| mmu-miR-708-3p | -0,6091 | 10,3103 | -2,3982 | 0,0255 | 0,1444 | -3,6383 |
| mmu-miR-127-3p | -0,4302 | 13,8646 | -2,3957 | 0,0256 | 0,1444 | -3,7128 |
| mmu-miR-185-3p | -0,4046 | 4,2468 | -2,3745 | 0,0268 | 0,1496 | -3,4837 |
| mmu-miR-421-3p | 0,3563 | 5,7973 | 2,3461 | 0,0285 | 0,1564 | -3,6116 |
| mmu-miR-24-2-5p | -0,3166 | 8,2476 | -2,3445 | 0,0286 | 0,1564 | -3,6941 |
| mmu-miR-107-3p | 0,6254 | 8,6016 | 2,3393 | 0,0289 | 0,1567 | -3,7125 |
| mmu-miR-191-3p | -0,3048 | 3,2851 | -2,3261 | 0,0297 | 0,1596 | -3,5153 |
| mmu-miR-221-3p | 0,4898 | 11,0091 | 2,3114 | 0,0306 | 0,1618 | -3,8106 |
| mmu-miR-181d-5p | -0,2164 | 10,2932 | -2,3109 | 0,0307 | 0,1618 | -3,7994 |
| mmu-miR-16-5p | 0,4630 | 9,7723 | 2,2950 | 0,0317 | 0,1658 | -3,8175 |
| mmu-miR-16-1-3p | -0,3216 | 5,8469 | -2,2813 | 0,0327 | 0,1692 | -3,7259 |
| mmu-miR-144-3p | 1,9482 | 1,7821 | 2,2765 | 0,0330 | 0,1693 | -3,4980 |
| mmu-miR-6239 | -1,0830 | 2,5903 | -2,2539 | 0,0346 | 0,1755 | -3,6028 |
| mmu-miR-135b-3p | -0,6633 | 1,2539 | -2,2510 | 0,0348 | 0,1755 | -3,5383 |
| mmu-miR-1982-3p | 0,4746 | 1,8157 | 2,2411 | 0,0355 | 0,1776 | -3,5719 |
| mmu-miR-378c | -0,3355 | 6,7138 | -2,2344 | 0,0360 | 0,1786 | -3,8488 |
| mmu-miR-151-5p | -0,2579 | 7,2709 | -2,2224 | 0,0369 | 0,1815 | -3,8888 |
| mmu-miR-148a-3p | -0,5984 | 12,1616 | -2,2069 | 0,0381 | 0,1845 | -4,0288 |
| mmu-miR-466c-5p | -0,4654 | 2,0243 | -2,2060 | 0,0382 | 0,1845 | -3,6447 |
| mmu-let-7b-5p | 0,3347 | 12,3291 | 2,2024 | 0,0385 | 0,1845 | -4,0376 |
| mmu-miR-29b-3p | 0,2549 | 9,5660 | 2,1983 | 0,0388 | 0,1845 | -3,9886 |
| mmu-miR-151-3p | -0,4802 | 10,6263 | -2,1936 | 0,0392 | 0,1848 | -4,0203 |
| mmu-miR-873a-3p | 0,3003 | 6,7766 | 2,1897 | 0,0395 | 0,1848 | -3,9284 |
| mmu-miR-210-5p | 0,5105 | 1,3922 | 2,1753 | 0,0407 | 0,1863 | -3,6583 |
| mmu-miR-195a-5p | 0,5446 | 7,4327 | 2,1714 | 0,0411 | 0,1863 | -3,9801 |
| mmu-miR-652-3p | 0,3467 | 8,2876 | 2,1702 | 0,0412 | 0,1863 | -4,0113 |
| mmu-miR-505-3p | 0,5874 | 1,8940 | 2,1702 | 0,0412 | 0,1863 | -3,6842 |
| mmu-miR-324-3p | 0,3830 | 4,2651 | 2,1648 | 0,0416 | 0,1869 | -3,8500 |
| mmu-miR-2137 | 1,9769 | 2,9477 | 2,1341 | 0,0443 | 0,1958 | -3,7961 |
| mmu-miR-669c-5p | -0,4948 | 4,9711 | -2,1310 | 0,0446 | 0,1958 | -3,9557 |
| mmu-miR-106b-5p | 0,4466 | 5,0693 | 2,1308 | 0,0446 | 0,1958 | -3,9546 |
| mmu-miR-221-5p | 0,1973 | 8,2505 | 2,1192 | 0,0457 | 0,1990 | -4,0971 |
| mmu-miR-676-5p | -0,4802 | 5,8182 | -2,1126 | 0,0463 | 0,2002 | -4,0293 |
| mmu-let-7d-3p | -0,2917 | 9,0173 | -2,1011 | 0,0474 | 0,2034 | -4,1502 |
| mmu-miR-330-5p | -0,2128 | 9,7848 | -2,0883 | 0,0487 | 0,2072 | -4,1858 |
| mmu-miR-29b-2-5p | 0,3769 | 4,9953 | 2,0816 | 0,0493 | 0,2074 | -4,0318 |
| mmu-miR-140-3p | 0,2183 | 10,2951 | 2,0805 | 0,0494 | 0,2074 | -4,2106 |
| mmu-miR-27b-5p | 0,3749 | 4,0515 | 2,0757 | 0,0499 | 0,2080 | -3,9880 |
| mmu-miR-671-3p | -0,6382 | 6,7595 | -2,0665 | 0,0509 | 0,2082 | -4,1418 |
| mmu-miR-485-5p | -0,4238 | 8,6829 | -2,0655 | 0,0510 | 0,2082 | -4,2054 |
| mmu-miR-23a-3p | 0,5373 | 8,9468 | 2,0622 | 0,0513 | 0,2082 | -4,2121 |
| mmu-miR-3473a | 1,7128 | 2,6178 | 2,0610 | 0,0514 | 0,2082 | -3,8957 |
| mmu-miR-3473e | 1,3793 | 1,2313 | 2,0513 | 0,0524 | 0,2108 | -3,8285 |
| mmu-miR-34c-5p | -0,4519 | 9,2719 | -2,0453 | 0,0531 | 0,2119 | -4,2494 |
| mmu-miR-330-3p | 0,2861 | 8,9063 | 2,0298 | 0,0547 | 0,2158 | -4,2686 |
| mmu-miR-7a-1-3p | 0,5982 | 4,7829 | 2,0292 | 0,0548 | 0,2158 | -4,1050 |
| mmu-miR-200a-3p | 0,6709 | 9,6182 | 2,0220 | 0,0556 | 0,2168 | -4,2923 |
| mmu-miR-1964-3p | -0,8531 | 5,0242 | -2,0202 | 0,0558 | 0,2168 | -4,1416 |
| mmu-miR-93-5p | 0,4720 | 7,8898 | 2,0047 | 0,0576 | 0,2221 | -4,2812 |
| mmu-miR-29a-5p | 0,4282 | 3,6780 | 1,9901 | 0,0592 | 0,2271 | -4,0986 |
| mmu-miR-669o-5p | -0,7246 | 1,1874 | -1,9751 | 0,0610 | 0,2308 | -3,9352 |
| mmu-miR-20a-5p | 0,6534 | 6,9253 | 1,9722 | 0,0614 | 0,2308 | -4,3008 |
| mmu-miR-96-5p | 1,0561 | 4,8198 | 1,9690 | 0,0618 | 0,2308 | -4,1811 |
| mmu-miR-145a-5p | 0,2971 | 8,5764 | 1,9686 | 0,0618 | 0,2308 | -4,3615 |
| mmu-miR-679-5p | -0,5030 | 4,0224 | -1,9596 | 0,0629 | 0,2335 | -4,1763 |
| mmu-miR-532-5p | -0,4320 | 10,5500 | -1,9494 | 0,0642 | 0,2367 | -4,4382 |
| mmu-miR-490-5p | 0,4444 | 1,4913 | 1,9432 | 0,0650 | 0,2368 | -4,0042 |
| mmu-miR-148a-5p | -0,5038 | 3,5791 | -1,9426 | 0,0651 | 0,2368 | -4,1744 |
| mmu-miR-6540-5p | 0,3758 | 5,4774 | 1,9387 | 0,0656 | 0,2372 | -4,2959 |
| mmu-miR-8112 | -0,4999 | 1,5600 | -1,9317 | 0,0665 | 0,2389 | -4,0359 |
| mmu-miR-195a-3p | -0,5483 | 5,7870 | -1,9262 | 0,0672 | 0,2400 | -4,3349 |
| mmu-let-7f-2-3p | -0,3061 | 5,7925 | -1,9200 | 0,0680 | 0,2414 | -4,3353 |
| mmu-miR-495-3p | -0,2517 | 9,8269 | -1,9094 | 0,0694 | 0,2450 | -4,4882 |
| mmu-miR-1940 | 0,5064 | 4,3738 | 1,8978 | 0,0710 | 0,2490 | -4,3024 |
| mmu-miR-6240 | 1,6886 | -0,1102 | 1,8935 | 0,0716 | 0,2496 | -4,0122 |
| mmu-miR-128-3p | -0,4006 | 17,4672 | -1,8897 | 0,0722 | 0,2499 | -4,6873 |
| mmu-miR-34b-5p | -0,7192 | 5,5542 | -1,8811 | 0,0734 | 0,2505 | -4,3869 |
| mmu-miR-770-5p | 0,2459 | 7,0290 | 1,8795 | 0,0736 | 0,2505 | -4,4577 |
| mmu-miR-30c-5p | 0,2928 | 13,2949 | 1,8793 | 0,0736 | 0,2505 | -4,6101 |
| mmu-miR-137-5p | 0,2983 | 3,0875 | 1,8721 | 0,0747 | 0,2525 | -4,2425 |
| mmu-miR-6979-3p | -0,6784 | 0,9607 | -1,8637 | 0,0759 | 0,2551 | -4,0842 |
| mmu-miR-1839-5p | 0,1946 | 10,0144 | 1,8608 | 0,0763 | 0,2551 | -4,5707 |
| mmu-miR-540-3p | -0,2093 | 5,7477 | -1,8501 | 0,0779 | 0,2589 | -4,4504 |
| mmu-miR-99a-5p | -0,2697 | 14,8414 | -1,8418 | 0,0791 | 0,2615 | -4,7075 |
| mmu-miR-3099-3p | 0,4278 | 5,7530 | 1,8340 | 0,0803 | 0,2635 | -4,4760 |
| mmu-miR-298-5p | -0,3993 | 5,8939 | -1,8319 | 0,0807 | 0,2635 | -4,4844 |
| mmu-miR-141-3p | 1,0200 | 3,0837 | 1,8252 | 0,0817 | 0,2653 | -4,2585 |
| mmu-miR-142-5p | 0,2712 | 5,9027 | 1,8195 | 0,0826 | 0,2654 | -4,4981 |
| mmu-miR-17-3p | -0,5389 | 1,7034 | -1,8193 | 0,0826 | 0,2654 | -4,1786 |
| mmu-miR-9-5p | -0,1429 | 17,1898 | -1,8096 | 0,0842 | 0,2688 | -4,8113 |
| mmu-miR-342-5p | -0,2797 | 4,1449 | -1,8046 | 0,0849 | 0,2699 | -4,4256 |
| mmu-miR-125a-5p | 0,2873 | 12,8454 | 1,7910 | 0,0872 | 0,2748 | -4,7410 |
| mmu-miR-7047-3p | 0,4336 | 2,6299 | 1,7882 | 0,0876 | 0,2748 | -4,3096 |
| mmu-miR-410-3p | 0,3936 | 9,2665 | 1,7863 | 0,0879 | 0,2748 | -4,6695 |
| mmu-miR-325-5p | 0,1811 | 6,3736 | 1,7774 | 0,0894 | 0,2768 | -4,5910 |
| mmu-miR-1843a-5p | -0,2839 | 10,1574 | -1,7768 | 0,0895 | 0,2768 | -4,7039 |
| mmu-miR-92a-3p | 0,2137 | 9,2016 | 1,7694 | 0,0908 | 0,2780 | -4,6952 |
| mmu-miR-361-5p | 0,4135 | 7,1098 | 1,7688 | 0,0909 | 0,2780 | -4,6311 |
| mmu-miR-193b-3p | -0,4041 | 4,6605 | -1,7640 | 0,0917 | 0,2781 | -4,5248 |
| mmu-miR-136-3p | -0,2379 | 11,6658 | -1,7630 | 0,0919 | 0,2781 | -4,7588 |
| mmu-miR-362-5p | 0,4948 | 4,5928 | 1,7522 | 0,0938 | 0,2813 | -4,5206 |
| mmu-miR-98-5p | 0,2709 | 8,9441 | 1,7513 | 0,0939 | 0,2813 | -4,7176 |
| mmu-miR-378d | -0,5640 | 6,0082 | -1,7461 | 0,0949 | 0,2821 | -4,6223 |
| mmu-miR-342-3p | 0,3950 | 9,0749 | 1,7442 | 0,0952 | 0,2821 | -4,7317 |
| mmu-miR-384-3p | 0,3022 | 7,6336 | 1,7342 | 0,0970 | 0,2841 | -4,7006 |
| mmu-miR-10b-5p | -0,4058 | 5,3597 | -1,7317 | 0,0974 | 0,2841 | -4,6118 |
| mmu-miR-15b-5p | 0,4267 | 3,4235 | 1,7304 | 0,0977 | 0,2841 | -4,4710 |
| mmu-miR-374b-5p | 0,3883 | 6,4156 | 1,7284 | 0,0980 | 0,2841 | -4,6616 |
| mmu-miR-29c-5p | -0,2336 | 5,6781 | -1,7268 | 0,0983 | 0,2841 | -4,6308 |
| mmu-miR-188-5p | -0,5705 | 1,7485 | -1,7061 | 0,1022 | 0,2938 | -4,3241 |
| mmu-miR-29a-3p | 0,2064 | 13,9201 | 1,6862 | 0,1060 | 0,3032 | -4,9264 |
| mmu-miR-378a-3p | -0,2563 | 10,4669 | -1,6645 | 0,1103 | 0,3126 | -4,8824 |
| mmu-miR-122-5p | -0,4085 | 4,0695 | -1,6642 | 0,1104 | 0,3126 | -4,6219 |
| mmu-miR-214-3p | 0,6812 | 0,5834 | 1,6548 | 0,1123 | 0,3164 | -4,3055 |
| mmu-miR-7015-3p | -0,4638 | 4,2828 | -1,6464 | 0,1140 | 0,3186 | -4,6653 |
| mmu-miR-21a-5p | 0,3149 | 13,9797 | 1,6458 | 0,1141 | 0,3186 | -4,9930 |
| mmu-miR-138-2-3p | 0,3080 | 4,0170 | 1,6406 | 0,1152 | 0,3191 | -4,6451 |
| mmu-miR-382-3p | 0,1938 | 9,2294 | 1,6396 | 0,1154 | 0,3191 | -4,8895 |
| mmu-miR-1943-5p | 0,5284 | 1,0988 | 1,6357 | 0,1162 | 0,3198 | -4,3923 |
| mmu-miR-744-5p | -0,2930 | 10,6067 | -1,6231 | 0,1189 | 0,3256 | -4,9461 |
| mmu-miR-3474 | -0,6521 | 0,3317 | -1,5663 | 0,1317 | 0,3588 | -4,4153 |
| mmu-miR-379-5p | -0,2611 | 11,8165 | -1,5532 | 0,1348 | 0,3655 | -5,0740 |
| mmu-miR-125b-5p | 0,2487 | 14,0820 | 1,5499 | 0,1355 | 0,3659 | -5,1277 |
| mmu-miR-1843b-5p | -0,2191 | 9,9943 | -1,5461 | 0,1365 | 0,3667 | -5,0393 |
| mmu-miR-26a-2-3p | -0,2542 | 3,5783 | -1,5434 | 0,1371 | 0,3667 | -4,7471 |
| mmu-miR-3084-3p | 0,5676 | 1,7107 | 1,5336 | 0,1395 | 0,3710 | -4,5361 |
| mmu-let-7g-3p | -0,4345 | 1,7152 | -1,5296 | 0,1405 | 0,3710 | -4,5478 |
| mmu-miR-598-3p | -0,2423 | 11,0918 | -1,5289 | 0,1407 | 0,3710 | -5,0882 |
| mmu-miR-7a-2-3p | 0,7289 | 2,0578 | 1,5253 | 0,1416 | 0,3713 | -4,6261 |
| mmu-miR-28a-3p | 0,2385 | 4,6005 | 1,5232 | 0,1421 | 0,3713 | -4,8480 |
| mmu-miR-7a-5p | 0,3118 | 13,0841 | 1,5198 | 0,1429 | 0,3718 | -5,1511 |
| mmu-miR-340-5p | 0,2070 | 11,9516 | 1,5056 | 0,1465 | 0,3767 | -5,1392 |
| mmu-miR-6395 | -0,4095 | 1,6485 | -1,5053 | 0,1466 | 0,3767 | -4,6058 |
| mmu-miR-193a-5p | 0,4647 | 0,8752 | 1,5007 | 0,1478 | 0,3767 | -4,5173 |
| mmu-miR-378a-5p | -0,2540 | 4,6550 | -1,4998 | 0,1480 | 0,3767 | -4,8805 |
| mmu-miR-92b-3p | 0,1984 | 9,2237 | 1,4995 | 0,1481 | 0,3767 | -5,0884 |
| mmu-miR-467d-3p | 0,5838 | 0,7269 | 1,4920 | 0,1500 | 0,3800 | -4,5213 |
| mmu-miR-29b-1-5p | 0,5672 | 1,1269 | 1,4882 | 0,1510 | 0,3808 | -4,5491 |
| mmu-miR-205-5p | 0,4129 | 1,7883 | 1,4707 | 0,1556 | 0,3883 | -4,6223 |
| mmu-miR-217-5p | -0,3632 | 2,1782 | -1,4679 | 0,1564 | 0,3883 | -4,6898 |
| mmu-miR-666-3p | -0,3592 | 1,5761 | -1,4658 | 0,1570 | 0,3883 | -4,6221 |
| mmu-miR-219a-5p | -0,2976 | 7,5028 | -1,4655 | 0,1570 | 0,3883 | -5,0735 |
| mmu-miR-551b-3p | -0,2952 | 4,4047 | -1,4644 | 0,1573 | 0,3883 | -4,9175 |
| mmu-miR-379-3p | 0,3294 | 6,4065 | 1,4553 | 0,1598 | 0,3911 | -5,0416 |
| mmu-miR-543-3p | -0,3558 | 10,1270 | -1,4534 | 0,1603 | 0,3911 | -5,1693 |
| mmu-miR-124-5p | 0,3123 | 7,5654 | 1,4527 | 0,1605 | 0,3911 | -5,0949 |
| mmu-miR-7b-5p | 0,2429 | 11,4874 | 1,4447 | 0,1627 | 0,3948 | -5,2141 |
| mmu-miR-149-5p | -0,1354 | 9,1787 | -1,4377 | 0,1647 | 0,3962 | -5,1689 |
| mmu-miR-128-1-5p | -0,1903 | 5,3178 | -1,4377 | 0,1647 | 0,3962 | -5,0107 |
| mmu-miR-3068-3p | 0,1699 | 6,5446 | 1,4310 | 0,1666 | 0,3991 | -5,0857 |
| mmu-miR-323-3p | -0,1637 | 8,4642 | -1,4244 | 0,1685 | 0,3995 | -5,1666 |
| mmu-miR-3066-5p | -0,4630 | 1,5060 | -1,4221 | 0,1691 | 0,3995 | -4,6751 |
| mmu-miR-429-3p | 0,4201 | 6,4758 | 1,4209 | 0,1695 | 0,3995 | -5,0867 |
| mmu-miR-541-5p | -0,1750 | 12,0420 | -1,4206 | 0,1695 | 0,3995 | -5,2578 |
| mmu-miR-412-3p | -0,3543 | 2,0303 | -1,4142 | 0,1714 | 0,4005 | -4,7400 |
| mmu-miR-667-3p | 0,2817 | 6,7617 | 1,4116 | 0,1722 | 0,4005 | -5,1181 |
| mmu-miR-3060-3p | 0,6256 | 0,7653 | 1,4109 | 0,1724 | 0,4005 | -4,5759 |
| mmu-miR-676-3p | -0,2322 | 7,4638 | -1,4096 | 0,1727 | 0,4005 | -5,1545 |
| mmu-miR-744-3p | -0,2555 | 2,5310 | -1,4069 | 0,1735 | 0,4008 | -4,8093 |
| mmu-miR-669a-5p | -0,2963 | 2,0995 | -1,4019 | 0,1750 | 0,4025 | -4,7712 |
| mmu-let-7c-1-3p | -0,2498 | 3,5305 | -1,3872 | 0,1794 | 0,4107 | -4,9393 |
| mmu-miR-204-5p | -0,3156 | 10,1016 | -1,3852 | 0,1800 | 0,4107 | -5,2566 |
| mmu-miR-346-5p | 0,1775 | 7,1615 | 1,3774 | 0,1824 | 0,4144 | -5,1786 |
| mmu-miR-1224-5p | 0,1886 | 6,4972 | 1,3686 | 0,1850 | 0,4189 | -5,1622 |
| mmu-miR-1981-5p | -0,4808 | 8,9760 | -1,3637 | 0,1866 | 0,4194 | -5,2608 |
| mmu-miR-7080-5p | 0,8648 | 0,1090 | 1,3632 | 0,1867 | 0,4194 | -4,6005 |
| mmu-miR-183-5p | 0,8135 | 8,4369 | 1,3541 | 0,1896 | 0,4241 | -5,2448 |
| mmu-miR-139-3p | -0,2287 | 7,4175 | -1,3513 | 0,1904 | 0,4244 | -5,2244 |
| mmu-miR-137-3p | 0,3238 | 8,9612 | 1,3339 | 0,1960 | 0,4351 | -5,2932 |
| mmu-miR-32-5p | -0,2052 | 5,2608 | -1,3284 | 0,1978 | 0,4374 | -5,1323 |
| mmu-miR-200b-3p | 0,4234 | 8,7806 | 1,3132 | 0,2028 | 0,4464 | -5,3113 |
| mmu-miR-27b-3p | 0,1132 | 13,4034 | 1,3105 | 0,2036 | 0,4464 | -5,4274 |
| mmu-miR-134-5p | -0,1656 | 7,7821 | -1,3058 | 0,2052 | 0,4464 | -5,2922 |
| mmu-miR-329-3p | -0,1455 | 7,4027 | -1,3037 | 0,2059 | 0,4464 | -5,2766 |
| mmu-miR-7093-3p | 0,3380 | 1,1873 | 1,3028 | 0,2062 | 0,4464 | -4,7663 |
| mmu-miR-1941-3p | 0,6055 | 0,5626 | 1,3020 | 0,2065 | 0,4464 | -4,6985 |
| mmu-miR-1249-3p | 0,2759 | 7,2503 | 1,2989 | 0,2075 | 0,4469 | -5,2774 |
| mmu-miR-93-3p | 0,4168 | 2,3275 | 1,2808 | 0,2137 | 0,4551 | -4,9149 |
| mmu-miR-490-3p | -0,1718 | 7,2563 | -1,2782 | 0,2146 | 0,4551 | -5,3036 |
| mmu-miR-326-3p | -0,2007 | 7,6375 | -1,2714 | 0,2170 | 0,4551 | -5,3246 |
| mmu-miR-494-3p | 0,4461 | 4,2188 | 1,2668 | 0,2186 | 0,4551 | -5,1206 |
| mmu-miR-324-5p | 0,2363 | 5,3795 | 1,2650 | 0,2192 | 0,4551 | -5,2252 |
| mmu-miR-488-3p | 0,1270 | 8,5884 | 1,2636 | 0,2197 | 0,4551 | -5,3678 |
| mmu-miR-30d-3p | -0,1773 | 4,8637 | -1,2636 | 0,2197 | 0,4551 | -5,1929 |
| mmu-miR-135a-1-3p | 0,2614 | 2,6488 | 1,2627 | 0,2200 | 0,4551 | -4,9870 |
| mmu-miR-499-5p | 0,2333 | 5,0299 | 1,2619 | 0,2203 | 0,4551 | -5,1996 |
| mmu-miR-382-5p | 0,1477 | 10,0843 | 1,2611 | 0,2206 | 0,4551 | -5,4082 |
| mmu-miR-3086-5p | 0,3261 | 1,9963 | 1,2610 | 0,2206 | 0,4551 | -4,8944 |
| mmu-miR-194-5p | 0,3815 | 7,1832 | 1,2604 | 0,2208 | 0,4551 | -5,3171 |
| mmu-miR-125b-1-3p | -0,2197 | 6,9141 | -1,2571 | 0,2220 | 0,4559 | -5,3170 |
| mmu-miR-6236 | 0,4601 | 4,6896 | 1,2531 | 0,2234 | 0,4572 | -5,2012 |
| mmu-miR-17-5p | 0,4044 | 5,7572 | 1,2448 | 0,2264 | 0,4616 | -5,2668 |
| mmu-miR-150-3p | -0,5418 | 0,6466 | -1,2426 | 0,2272 | 0,4616 | -4,7468 |
| mmu-miR-133a-3p | 0,3745 | 5,2068 | 1,2380 | 0,2289 | 0,4622 | -5,2321 |
| mmu-miR-493-3p | -0,3545 | 1,3944 | -1,2373 | 0,2291 | 0,4622 | -4,8406 |
| mmu-miR-879-3p | 0,3145 | 1,8243 | 1,2310 | 0,2314 | 0,4637 | -4,9080 |
| mmu-miR-5100 | 0,3889 | 1,5094 | 1,2309 | 0,2315 | 0,4637 | -4,8798 |
| mmu-miR-200b-5p | 0,5945 | 1,8228 | 1,2275 | 0,2327 | 0,4646 | -4,8911 |
| mmu-miR-7688-5p | -0,2710 | 2,7312 | -1,2243 | 0,2339 | 0,4653 | -5,0529 |
| mmu-miR-6769b-3p | -0,2886 | 2,7579 | -1,2219 | 0,2348 | 0,4655 | -5,0563 |
| mmu-miR-497-5p | 0,2351 | 5,6035 | 1,2185 | 0,2360 | 0,4664 | -5,2916 |
| mmu-miR-3072-3p | 0,2285 | 4,0207 | 1,2115 | 0,2386 | 0,4699 | -5,1823 |
| mmu-miR-3547-3p | 0,8387 | 0,2040 | 1,2059 | 0,2408 | 0,4725 | -4,7706 |
| mmu-miR-3093-5p | 0,2836 | 1,6759 | 1,1934 | 0,2455 | 0,4788 | -4,9226 |
| mmu-miR-9-3p | 0,1575 | 12,5696 | 1,1918 | 0,2462 | 0,4788 | -5,5463 |
| mmu-miR-24-3p | 0,1301 | 12,6791 | 1,1896 | 0,2470 | 0,4788 | -5,5527 |
| mmu-miR-30d-5p | -0,1640 | 14,4939 | -1,1887 | 0,2473 | 0,4788 | -5,5964 |
| mmu-miR-434-3p | 0,1641 | 12,3461 | 1,1788 | 0,2512 | 0,4830 | -5,5546 |
| mmu-miR-101b-3p | 0,1192 | 11,6851 | 1,1788 | 0,2512 | 0,4830 | -5,5413 |
| mmu-miR-381-5p | -0,6276 | 0,2942 | -1,1735 | 0,2533 | 0,4847 | -4,7537 |
| mmu-miR-24-1-5p | -0,1416 | 5,6248 | -1,1714 | 0,2540 | 0,4847 | -5,3418 |
| mmu-miR-411-5p | -0,1238 | 12,3997 | -1,1701 | 0,2546 | 0,4847 | -5,5678 |
| mmu-miR-6975-3p | -0,4007 | 1,1208 | -1,1664 | 0,2561 | 0,4859 | -4,8817 |
| mmu-miR-1191 | -0,2593 | 2,2442 | -1,1631 | 0,2573 | 0,4868 | -5,0148 |
| mmu-miR-1843a-3p | -0,2005 | 6,8654 | -1,1607 | 0,2583 | 0,4870 | -5,4196 |
| mmu-miR-3061-3p | -0,4398 | 1,0333 | -1,1533 | 0,2613 | 0,4901 | -4,8924 |
| mmu-miR-6989-3p | -0,2187 | 3,0456 | -1,1523 | 0,2616 | 0,4901 | -5,1579 |
| mmu-miR-301a-3p | 0,2230 | 3,0414 | 1,1482 | 0,2633 | 0,4916 | -5,1528 |
| mmu-miR-6906-3p | -0,4164 | 1,5634 | -1,1413 | 0,2661 | 0,4946 | -4,9667 |
| mmu-miR-30c-2-3p | -0,2354 | 7,7441 | -1,1399 | 0,2667 | 0,4946 | -5,4796 |
| mmu-miR-370-5p | -0,1632 | 5,3123 | -1,1213 | 0,2743 | 0,5064 | -5,3797 |
| mmu-miR-142-3p | 0,2755 | 4,4640 | 1,1203 | 0,2748 | 0,5064 | -5,3045 |
| mmu-miR-331-3p | -0,1694 | 6,2612 | -1,1169 | 0,2762 | 0,5064 | -5,4357 |
| mmu-miR-467c-5p | -0,2718 | 1,8396 | -1,1160 | 0,2766 | 0,5064 | -5,0448 |
| mmu-miR-301a-5p | 0,2130 | 4,3395 | 1,1108 | 0,2787 | 0,5077 | -5,3078 |
| mmu-miR-704 | -0,4353 | 1,7151 | -1,1060 | 0,2808 | 0,5077 | -5,0171 |
| mmu-miR-7075-3p | -0,2629 | 3,1196 | -1,1055 | 0,2810 | 0,5077 | -5,2145 |
| mmu-miR-1843b-3p | -0,2917 | 7,4636 | -1,1046 | 0,2814 | 0,5077 | -5,5053 |
| mmu-miR-3093-3p | 0,2390 | 2,3479 | 1,1039 | 0,2817 | 0,5077 | -5,1253 |
| mmu-miR-145a-3p | 0,1566 | 6,9124 | 1,1014 | 0,2827 | 0,5080 | -5,4810 |
| mmu-miR-3963 | 0,4590 | 1,4902 | 1,0858 | 0,2894 | 0,5184 | -4,9837 |
| mmu-miR-186-5p | -0,1509 | 9,3345 | -1,0818 | 0,2911 | 0,5188 | -5,5859 |
| mmu-let-7a-2-3p | -0,2435 | 1,5481 | -1,0778 | 0,2929 | 0,5188 | -5,0293 |
| mmu-miR-1197-3p | -0,3722 | 0,8282 | -1,0769 | 0,2933 | 0,5188 | -4,9304 |
| mmu-miR-299a-5p | -0,2251 | 4,0351 | -1,0765 | 0,2935 | 0,5188 | -5,3200 |
| mmu-miR-375-3p | -0,2513 | 5,2370 | -1,0749 | 0,2942 | 0,5188 | -5,4267 |
| mmu-miR-322-3p | -0,1684 | 5,5923 | -1,0727 | 0,2951 | 0,5189 | -5,4456 |
| mmu-miR-344d-3p | 0,1369 | 8,0181 | 1,0693 | 0,2966 | 0,5200 | -5,5596 |
| mmu-miR-532-3p | 0,3748 | 5,2193 | 1,0668 | 0,2977 | 0,5203 | -5,4209 |
| mmu-miR-340-3p | -0,1709 | 8,5763 | -1,0637 | 0,2991 | 0,5206 | -5,5861 |
| mmu-miR-199a-5p | 0,1907 | 5,2153 | 1,0623 | 0,2997 | 0,5206 | -5,4221 |
| mmu-miR-101c | -0,2631 | 4,9463 | -1,0574 | 0,3019 | 0,5229 | -5,4153 |
| mmu-miR-339-3p | 0,1458 | 3,8765 | 1,0470 | 0,3066 | 0,5285 | -5,3368 |
| mmu-miR-365-3p | 0,2588 | 6,0135 | 1,0461 | 0,3070 | 0,5285 | -5,4873 |
| mmu-miR-154-3p | 0,2481 | 3,9259 | 1,0431 | 0,3083 | 0,5292 | -5,3425 |
| mmu-miR-136-5p | -0,1395 | 9,1983 | -1,0390 | 0,3102 | 0,5300 | -5,6255 |
| mmu-miR-222-3p | 0,1294 | 10,0527 | 1,0380 | 0,3106 | 0,5300 | -5,6468 |
| mmu-miR-6988-3p | -0,2283 | 3,4628 | -1,0355 | 0,3118 | 0,5300 | -5,3240 |
| mmu-miR-106b-3p | 0,1067 | 6,5004 | 1,0340 | 0,3125 | 0,5300 | -5,5336 |
| mmu-miR-3087-3p | -0,2005 | 2,7304 | -1,0285 | 0,3150 | 0,5324 | -5,2053 |
| mmu-miR-491-5p | 0,4471 | 1,7117 | 1,0268 | 0,3157 | 0,5324 | -5,0767 |
| mmu-miR-423-3p | -0,1584 | 9,4839 | -1,0195 | 0,3191 | 0,5344 | -5,6539 |
| mmu-miR-450b-5p | -0,2911 | 1,3600 | -1,0188 | 0,3194 | 0,5344 | -5,0313 |
| mmu-miR-339-5p | 0,2368 | 5,6745 | 1,0182 | 0,3197 | 0,5344 | -5,4955 |
| mmu-miR-146b-3p | 0,2948 | 1,7353 | 1,0131 | 0,3221 | 0,5368 | -5,1088 |
| mmu-miR-410-5p | 0,3218 | 1,6365 | 1,0087 | 0,3242 | 0,5382 | -5,0942 |
| mmu-miR-125a-3p | 0,1541 | 4,1484 | 1,0058 | 0,3255 | 0,5382 | -5,4049 |
| mmu-miR-219a-1-3p | -0,3082 | 2,0911 | -1,0039 | 0,3264 | 0,5382 | -5,1610 |
| mmu-miR-6896-5p | 0,2728 | 2,0380 | 1,0033 | 0,3267 | 0,5382 | -5,1534 |
| mmu-miR-1968-5p | -0,2021 | 1,9753 | -0,9983 | 0,3291 | 0,5402 | -5,1502 |
| mmu-miR-5129-3p | -0,2524 | 4,2202 | -0,9954 | 0,3305 | 0,5402 | -5,4178 |
| mmu-miR-3061-5p | -0,3003 | 1,4018 | -0,9948 | 0,3307 | 0,5402 | -5,0827 |
| mmu-miR-6952-3p | -0,2914 | 1,7846 | -0,9921 | 0,3320 | 0,5408 | -5,1283 |
| mmu-miR-143-5p | -0,2140 | 4,0695 | -0,9818 | 0,3369 | 0,5473 | -5,4015 |
| mmu-miR-29c-3p | 0,1795 | 7,9078 | 0,9684 | 0,3435 | 0,5563 | -5,6518 |
| mmu-miR-434-5p | -0,1104 | 12,7682 | -0,9581 | 0,3485 | 0,5626 | -5,7922 |
| mmu-miR-1947-5p | -0,2846 | 2,5220 | -0,9565 | 0,3493 | 0,5626 | -5,2821 |
| mmu-miR-369-3p | 0,3283 | 8,1854 | 0,9479 | 0,3536 | 0,5679 | -5,6778 |
| mmu-miR-8114 | 0,2166 | 2,6919 | 0,9288 | 0,3631 | 0,5816 | -5,3138 |
| mmu-miR-211-5p | -0,2448 | 3,3655 | -0,9133 | 0,3711 | 0,5927 | -5,4017 |
| mmu-miR-592-5p | 0,1293 | 7,7870 | 0,9094 | 0,3731 | 0,5930 | -5,6988 |
| mmu-miR-3069-3p | 0,2142 | 1,4736 | 0,9067 | 0,3745 | 0,5930 | -5,1570 |
| mmu-miR-30e-5p | 0,1549 | 12,7892 | 0,9065 | 0,3746 | 0,5930 | -5,8367 |
| mmu-miR-6982-3p | -0,2318 | 1,6467 | -0,9050 | 0,3754 | 0,5930 | -5,1878 |
| mmu-miR-30b-5p | 0,2062 | 9,0345 | 0,9016 | 0,3771 | 0,5937 | -5,7491 |
| mmu-miR-467e-5p | -0,1694 | 3,2036 | -0,8974 | 0,3793 | 0,5937 | -5,3987 |
| mmu-miR-126a-3p | 0,0854 | 13,3867 | 0,8963 | 0,3799 | 0,5937 | -5,8624 |
| mmu-miR-154-5p | -0,1755 | 7,1243 | -0,8961 | 0,3800 | 0,5937 | -5,6857 |
| mmu-miR-409-5p | -0,1534 | 8,6237 | -0,8814 | 0,3877 | 0,6041 | -5,7552 |
| mmu-miR-3084-5p | -0,2482 | 2,0128 | -0,8738 | 0,3918 | 0,6083 | -5,2501 |
| mmu-miR-431-5p | 0,1497 | 4,5899 | 0,8712 | 0,3931 | 0,6083 | -5,5602 |
| mmu-let-7k | 0,1843 | 2,6821 | 0,8704 | 0,3936 | 0,6083 | -5,3629 |
| mmu-miR-1264-5p | 0,2742 | 3,4390 | 0,8627 | 0,3977 | 0,6120 | -5,4694 |
| mmu-miR-130b-5p | -0,1829 | 5,4018 | -0,8602 | 0,3990 | 0,6120 | -5,6234 |
| mmu-miR-6516-3p | 0,5113 | 1,9239 | 0,8600 | 0,3992 | 0,6120 | -5,2547 |
| mmu-miR-3068-5p | 0,0948 | 7,1386 | 0,8554 | 0,4016 | 0,6142 | -5,7216 |
| mmu-miR-100-5p | -0,1527 | 13,5082 | -0,8450 | 0,4073 | 0,6212 | -5,9106 |
| mmu-miR-505-5p | 0,2017 | 0,9996 | 0,8410 | 0,4095 | 0,6229 | -5,1269 |
| mmu-miR-3083-5p | 0,1134 | 3,9895 | 0,8368 | 0,4118 | 0,6232 | -5,5374 |
| mmu-miR-338-3p | 0,1698 | 8,2799 | 0,8367 | 0,4118 | 0,6232 | -5,7798 |
| mmu-miR-350-5p | 0,4006 | 1,3238 | 0,8310 | 0,4150 | 0,6255 | -5,1457 |
| mmu-miR-7019-3p | 0,2432 | 1,1225 | 0,8300 | 0,4155 | 0,6255 | -5,1620 |
| mmu-miR-300-5p | 0,1879 | 4,0060 | 0,8251 | 0,4183 | 0,6275 | -5,5423 |
| mmu-miR-125b-2-3p | -0,0734 | 8,5462 | -0,8225 | 0,4197 | 0,6275 | -5,8045 |
| mmu-miR-708-5p | 0,2146 | 8,8120 | 0,8216 | 0,4202 | 0,6275 | -5,8104 |
| mmu-miR-3082-3p | -0,1592 | 2,7186 | -0,8076 | 0,4281 | 0,6362 | -5,4262 |
| mmu-miR-323-5p | -0,1427 | 3,5617 | -0,8073 | 0,4282 | 0,6362 | -5,5180 |
| mmu-miR-6944-3p | -0,1298 | 4,0442 | -0,8024 | 0,4310 | 0,6384 | -5,5729 |
| mmu-miR-181b-5p | 0,0890 | 12,0080 | 0,8008 | 0,4319 | 0,6384 | -5,9069 |
| mmu-miR-331-5p | 0,2501 | 2,3328 | 0,7817 | 0,4428 | 0,6509 | -5,3548 |
| mmu-miR-467d-5p | -0,2677 | 1,3604 | -0,7817 | 0,4428 | 0,6509 | -5,2288 |
| mmu-miR-126a-5p | 0,0841 | 9,4400 | 0,7801 | 0,4437 | 0,6509 | -5,8578 |
| mmu-miR-15b-3p | -0,1709 | 1,7026 | -0,7688 | 0,4502 | 0,6588 | -5,2987 |
| mmu-miR-15a-5p | 0,1658 | 4,8322 | 0,7621 | 0,4541 | 0,6628 | -5,6530 |
| mmu-miR-362-3p | 0,3260 | 3,4077 | 0,7589 | 0,4560 | 0,6638 | -5,4956 |
| mmu-miR-34c-3p | -0,2218 | 2,7252 | -0,7527 | 0,4597 | 0,6675 | -5,4546 |
| mmu-miR-674-5p | 0,1040 | 6,9279 | 0,7457 | 0,4638 | 0,6717 | -5,7955 |
| mmu-miR-1949 | 0,2865 | 1,4050 | 0,7435 | 0,4651 | 0,6720 | -5,2525 |
| mmu-miR-3102-3p.2-3p | 0,1998 | 1,6791 | 0,7368 | 0,4691 | 0,6760 | -5,2834 |
| mmu-miR-27a-5p | 0,1498 | 4,1575 | 0,7278 | 0,4745 | 0,6816 | -5,6429 |
| mmu-miR-329-5p | 0,0643 | 8,3298 | 0,7264 | 0,4753 | 0,6816 | -5,8677 |
| mmu-miR-3078-5p | -0,1558 | 2,7178 | -0,7199 | 0,4792 | 0,6854 | -5,4644 |
| mmu-miR-3057-5p | 0,1691 | 2,9144 | 0,7172 | 0,4809 | 0,6861 | -5,4958 |
| mmu-miR-34a-5p | -0,1746 | 7,6683 | -0,7116 | 0,4842 | 0,6892 | -5,8492 |
| mmu-miR-877-5p | 0,2352 | 2,6211 | 0,7025 | 0,4898 | 0,6946 | -5,4556 |
| mmu-miR-669o-3p | -0,2107 | 1,3147 | -0,7015 | 0,4904 | 0,6946 | -5,2786 |
| mmu-miR-3535 | 0,1811 | 7,1841 | 0,6961 | 0,4937 | 0,6974 | -5,8464 |
| mmu-miR-26a-5p | 0,0876 | 15,4319 | 0,6942 | 0,4949 | 0,6974 | -6,0703 |
| mmu-miR-5121 | 0,2826 | 3,3221 | 0,6882 | 0,4986 | 0,7009 | -5,5758 |
| mmu-miR-3103-3p | 0,2053 | 0,7341 | 0,6855 | 0,5002 | 0,7015 | -5,1985 |
| mmu-miR-376a-3p | -0,0927 | 4,5465 | -0,6820 | 0,5024 | 0,7022 | -5,6910 |
| mmu-miR-503-3p | -0,2176 | 1,5424 | -0,6808 | 0,5032 | 0,7022 | -5,2834 |
| mmu-miR-204-3p | -0,1955 | 3,5110 | -0,6735 | 0,5077 | 0,7054 | -5,6097 |
| mmu-miR-449a-5p | 0,2742 | 1,3173 | 0,6715 | 0,5089 | 0,7054 | -5,2997 |
| mmu-miR-455-5p | -0,1024 | 6,7097 | -0,6712 | 0,5091 | 0,7054 | -5,8398 |
| mmu-miR-92b-5p | 0,1095 | 4,1582 | 0,6625 | 0,5146 | 0,7085 | -5,6781 |
| mmu-miR-574-3p | 0,1553 | 4,3750 | 0,6619 | 0,5150 | 0,7085 | -5,6925 |
| mmu-miR-6911-3p | -0,1551 | 2,7953 | -0,6617 | 0,5151 | 0,7085 | -5,5156 |
| mmu-miR-872-5p | 0,1014 | 9,0728 | 0,6452 | 0,5255 | 0,7212 | -5,9443 |
| mmu-miR-668-3p | 0,0861 | 8,1820 | 0,6350 | 0,5320 | 0,7263 | -5,9225 |
| mmu-miR-7226-3p | 0,1590 | 3,0043 | 0,6321 | 0,5339 | 0,7263 | -5,5739 |
| mmu-miR-187-5p | -0,1951 | 1,2997 | -0,6319 | 0,5340 | 0,7263 | -5,2967 |
| mmu-miR-380-3p | 0,1063 | 7,1844 | 0,6282 | 0,5364 | 0,7263 | -5,8830 |
| mmu-miR-139-5p | -0,0815 | 12,1649 | -0,6277 | 0,5367 | 0,7263 | -6,0356 |
| mmu-miR-677-5p | -0,1839 | 2,1370 | -0,6275 | 0,5368 | 0,7263 | -5,4604 |
| mmu-miR-218-2-3p | -0,1553 | 1,6926 | -0,6244 | 0,5388 | 0,7268 | -5,4033 |
| mmu-miR-664-5p | 0,0972 | 4,6059 | 0,6230 | 0,5398 | 0,7268 | -5,7352 |
| mmu-miR-433-3p | 0,0733 | 11,0153 | 0,6153 | 0,5447 | 0,7318 | -6,0112 |
| mmu-miR-33-5p | -0,1312 | 4,9699 | -0,6074 | 0,5499 | 0,7370 | -5,7576 |
| mmu-miR-135a-5p | 0,1099 | 8,3123 | 0,6022 | 0,5532 | 0,7384 | -5,9442 |
| mmu-miR-667-5p | -0,1179 | 4,8856 | -0,6018 | 0,5535 | 0,7384 | -5,7709 |
| mmu-miR-150-5p | -0,0838 | 9,1691 | -0,5896 | 0,5615 | 0,7474 | -5,9792 |
| mmu-miR-3962 | -0,1554 | 1,1266 | -0,5866 | 0,5635 | 0,7483 | -5,2872 |
| mmu-miR-760-3p | 0,0760 | 6,4040 | 0,5832 | 0,5658 | 0,7496 | -5,8803 |
| mmu-miR-5615-5p | -0,1418 | 1,5045 | -0,5800 | 0,5678 | 0,7506 | -5,3559 |
| mmu-miR-133b-3p | 0,2535 | 1,2159 | 0,5759 | 0,5705 | 0,7524 | -5,2946 |
| mmu-let-7e-3p | -0,0757 | 5,0391 | -0,5639 | 0,5786 | 0,7583 | -5,7995 |
| mmu-miR-665-3p | -0,1798 | 2,9728 | -0,5631 | 0,5791 | 0,7583 | -5,5539 |
| mmu-miR-369-5p | -0,0569 | 9,4611 | -0,5599 | 0,5813 | 0,7583 | -6,0041 |
| mmu-miR-31-3p | -0,1597 | 1,6020 | -0,5595 | 0,5815 | 0,7583 | -5,3693 |
| mmu-miR-504-5p | -0,1180 | 5,0101 | -0,5594 | 0,5816 | 0,7583 | -5,8075 |
| mmu-miR-574-5p | -0,0925 | 3,0662 | -0,5557 | 0,5841 | 0,7599 | -5,6185 |
| mmu-miR-19b-3p | 0,1260 | 6,0293 | 0,5494 | 0,5883 | 0,7636 | -5,8675 |
| mmu-miR-702-3p | 0,1589 | 2,7061 | 0,5464 | 0,5903 | 0,7645 | -5,5731 |
| mmu-miR-322-5p | 0,1244 | 3,9482 | 0,5438 | 0,5921 | 0,7650 | -5,7119 |
| mmu-miR-664-3p | 0,1106 | 6,6999 | 0,5394 | 0,5951 | 0,7672 | -5,9088 |
| mmu-miR-1306-3p | -0,1620 | 1,4885 | -0,5367 | 0,5969 | 0,7678 | -5,3829 |
| mmu-miR-1188-5p | -0,2123 | 0,8088 | -0,5305 | 0,6011 | 0,7715 | -5,2589 |
| mmu-miR-380-5p | 0,0621 | 6,9099 | 0,5258 | 0,6043 | 0,7739 | -5,9326 |
| mmu-miR-187-3p | 0,1055 | 5,7317 | 0,5238 | 0,6057 | 0,7740 | -5,8745 |
| mmu-miR-10a-5p | -0,1154 | 5,5224 | -0,5189 | 0,6090 | 0,7741 | -5,8630 |
| mmu-miR-700-5p | -0,0813 | 4,5379 | -0,5185 | 0,6093 | 0,7741 | -5,7900 |
| mmu-miR-673-5p | -0,0895 | 5,2657 | -0,5177 | 0,6099 | 0,7741 | -5,8444 |
| mmu-miR-28a-5p | 0,0788 | 4,9630 | 0,5141 | 0,6124 | 0,7756 | -5,8263 |
| mmu-miR-496a-3p | 0,1252 | 5,2548 | 0,5119 | 0,6139 | 0,7757 | -5,8393 |
| mmu-miR-770-3p | -0,1107 | 8,5325 | -0,5056 | 0,6182 | 0,7790 | -6,0120 |
| mmu-miR-484 | 0,0925 | 8,4800 | 0,5014 | 0,6211 | 0,7790 | -6,0060 |
| mmu-let-7f-5p | 0,0724 | 14,9869 | 0,4960 | 0,6249 | 0,7790 | -6,1775 |
| mmu-miR-7043-3p | -0,1545 | 1,3916 | -0,4955 | 0,6252 | 0,7790 | -5,3849 |
| mmu-miR-1298-5p | -0,2341 | 8,0802 | -0,4951 | 0,6254 | 0,7790 | -6,0024 |
| mmu-let-7i-5p | -0,0656 | 14,5635 | -0,4943 | 0,6260 | 0,7790 | -6,1682 |
| mmu-miR-99a-3p | -0,0899 | 2,9314 | -0,4939 | 0,6263 | 0,7790 | -5,6236 |
| mmu-miR-3085-3p | 0,1070 | 2,8478 | 0,4925 | 0,6273 | 0,7790 | -5,6038 |
| mmu-miR-212-3p | -0,0958 | 7,9036 | -0,4859 | 0,6319 | 0,7831 | -5,9941 |
| mmu-miR-1839-3p | 0,1092 | 4,3925 | 0,4789 | 0,6368 | 0,7850 | -5,7895 |
| mmu-miR-487b-3p | 0,0638 | 8,0081 | 0,4787 | 0,6369 | 0,7850 | -6,0008 |
| mmu-miR-32-3p | 0,2262 | 0,8963 | 0,4736 | 0,6405 | 0,7850 | -5,3197 |
| mmu-miR-143-3p | -0,0982 | 14,0407 | -0,4735 | 0,6405 | 0,7850 | -6,1643 |
| mmu-miR-140-5p | 0,0976 | 8,2459 | 0,4706 | 0,6426 | 0,7850 | -6,0099 |
| mmu-miR-872-3p | 0,0780 | 5,9932 | 0,4696 | 0,6433 | 0,7850 | -5,9076 |
| mmu-miR-30e-3p | -0,0670 | 9,5591 | -0,4687 | 0,6439 | 0,7850 | -6,0540 |
| mmu-miR-7080-3p | -0,1581 | 1,2839 | -0,4680 | 0,6444 | 0,7850 | -5,4031 |
| mmu-miR-7b-3p | 0,1765 | 1,6226 | 0,4608 | 0,6495 | 0,7896 | -5,4041 |
| mmu-miR-181a-5p | -0,0481 | 14,0778 | -0,4571 | 0,6521 | 0,7911 | -6,1720 |
| mmu-miR-672-3p | 0,1394 | 1,2223 | 0,4546 | 0,6539 | 0,7915 | -5,3664 |
| mmu-miR-26b-3p | 0,0995 | 2,1284 | 0,4455 | 0,6603 | 0,7977 | -5,5059 |
| mmu-miR-5099 | -0,1612 | 3,1704 | -0,4235 | 0,6761 | 0,8150 | -5,7060 |
| mmu-miR-6953-3p | -0,1650 | 0,4353 | -0,4198 | 0,6787 | 0,8158 | -5,2686 |
| mmu-miR-3475-3p | -0,0649 | 4,6443 | -0,4186 | 0,6796 | 0,8158 | -5,8422 |
| mmu-miR-337-5p | 0,0919 | 4,7362 | 0,4101 | 0,6857 | 0,8193 | -5,8440 |
| mmu-miR-23b-5p | 0,1033 | 1,1969 | 0,4080 | 0,6873 | 0,8193 | -5,3748 |
| mmu-miR-152-5p | 0,1076 | 2,7678 | 0,4078 | 0,6874 | 0,8193 | -5,6555 |
| mmu-miR-1193-3p | 0,1229 | 2,6224 | 0,4065 | 0,6884 | 0,8193 | -5,5916 |
| mmu-miR-215-5p | 0,1148 | 1,0871 | 0,4047 | 0,6896 | 0,8193 | -5,3676 |
| mmu-miR-345-5p | 0,0858 | 3,7804 | 0,3958 | 0,6961 | 0,8252 | -5,7640 |
| mmu-miR-130a-3p | 0,0821 | 3,6855 | 0,3914 | 0,6993 | 0,8253 | -5,7616 |
| mmu-miR-19a-3p | -0,0989 | 2,9878 | -0,3912 | 0,6995 | 0,8253 | -5,6694 |
| mmu-miR-377-3p | -0,0750 | 5,1879 | -0,3898 | 0,7004 | 0,8253 | -5,8854 |
| mmu-miR-378b | 0,0641 | 3,0306 | 0,3860 | 0,7032 | 0,8264 | -5,6875 |
| mmu-miR-8103 | -0,1337 | 1,6475 | -0,3846 | 0,7042 | 0,8264 | -5,4708 |
| mmu-miR-1224-3p | 0,0657 | 3,5137 | 0,3728 | 0,7129 | 0,8348 | -5,7396 |
| mmu-miR-30c-1-3p | -0,0465 | 4,0622 | -0,3611 | 0,7215 | 0,8425 | -5,8095 |
| mmu-miR-425-5p | 0,0367 | 6,9468 | 0,3581 | 0,7237 | 0,8425 | -6,0082 |
| mmu-miR-500-3p | 0,0933 | 2,7942 | 0,3569 | 0,7246 | 0,8425 | -5,6492 |
| mmu-miR-6899-3p | -0,1170 | 2,6184 | -0,3557 | 0,7255 | 0,8425 | -5,6514 |
| mmu-miR-148b-3p | 0,0372 | 10,6794 | 0,3540 | 0,7268 | 0,8425 | -6,1275 |
| mmu-miR-27a-3p | -0,0392 | 10,2943 | -0,3465 | 0,7323 | 0,8472 | -6,1183 |
| mmu-miR-30a-5p | -0,0522 | 14,6061 | -0,3419 | 0,7357 | 0,8479 | -6,2312 |
| mmu-miR-350-3p | 0,1026 | 4,6117 | 0,3417 | 0,7358 | 0,8479 | -5,8567 |
| mmu-miR-199b-5p | -0,1032 | 1,9345 | -0,3385 | 0,7382 | 0,8488 | -5,4822 |
| mmu-miR-543-5p | 0,0919 | 2,3228 | 0,3362 | 0,7400 | 0,8488 | -5,6043 |
| mmu-miR-124-3p | 0,0566 | 12,8379 | 0,3347 | 0,7411 | 0,8488 | -6,1892 |
| mmu-miR-219a-2-3p | -0,0642 | 11,3016 | -0,3229 | 0,7499 | 0,8557 | -6,1529 |
| mmu-miR-7070-3p | -0,1022 | 1,0294 | -0,3194 | 0,7525 | 0,8557 | -5,3591 |
| mmu-miR-30a-3p | -0,0404 | 9,8746 | -0,3189 | 0,7529 | 0,8557 | -6,1192 |
| mmu-miR-493-5p | -0,0570 | 3,5277 | -0,3186 | 0,7531 | 0,8557 | -5,7715 |
| mmu-miR-7044-3p | -0,1009 | 3,0074 | -0,3166 | 0,7545 | 0,8557 | -5,7216 |
| mmu-miR-98-3p | 0,1095 | 1,4208 | 0,3145 | 0,7561 | 0,8558 | -5,3986 |
| mmu-miR-344h-3p | 0,1116 | 2,1125 | 0,3066 | 0,7621 | 0,8594 | -5,5812 |
| mmu-miR-425-3p | 0,0448 | 4,5879 | 0,3063 | 0,7623 | 0,8594 | -5,8714 |
| mmu-miR-129-2-3p | 0,0482 | 9,9116 | 0,2973 | 0,7691 | 0,8654 | -6,1264 |
| mmu-miR-344c-3p | -0,0389 | 4,7900 | -0,2916 | 0,7734 | 0,8675 | -5,8908 |
| mmu-miR-672-5p | -0,0419 | 5,0206 | -0,2865 | 0,7772 | 0,8675 | -5,9198 |
| mmu-miR-3064-5p | -0,1045 | 0,9224 | -0,2852 | 0,7781 | 0,8675 | -5,4146 |
| mmu-miR-148b-5p | -0,0342 | 5,0290 | -0,2838 | 0,7792 | 0,8675 | -5,9182 |
| mmu-miR-5621-3p | 0,0927 | 0,8474 | 0,2838 | 0,7793 | 0,8675 | -5,3826 |
| mmu-miR-431-3p | -0,0362 | 6,4187 | -0,2828 | 0,7800 | 0,8675 | -6,0071 |
| mmu-miR-448-3p | 0,1145 | 3,2940 | 0,2654 | 0,7932 | 0,8800 | -5,7700 |
| mmu-miR-433-5p | 0,0356 | 4,7646 | 0,2623 | 0,7955 | 0,8800 | -5,9025 |
| mmu-miR-30b-3p | -0,0407 | 4,6822 | -0,2620 | 0,7958 | 0,8800 | -5,8853 |
| mmu-miR-138-1-3p | -0,0477 | 3,8290 | -0,2507 | 0,8044 | 0,8878 | -5,8185 |
| mmu-miR-7224-3p | -0,0467 | 3,1421 | -0,2234 | 0,8253 | 0,9077 | -5,7488 |
| mmu-miR-486-3p | 0,0538 | 2,0431 | 0,2229 | 0,8257 | 0,9077 | -5,5767 |
| mmu-miR-337-3p | -0,0825 | 1,6224 | -0,2154 | 0,8314 | 0,9123 | -5,4808 |
| mmu-miR-874-3p | 0,0275 | 7,4771 | 0,2080 | 0,8371 | 0,9158 | -6,0709 |
| mmu-miR-1a-3p | 0,0393 | 10,1522 | 0,2072 | 0,8377 | 0,9158 | -6,1540 |
| mmu-miR-877-3p | 0,0315 | 5,1526 | 0,2036 | 0,8406 | 0,9171 | -5,9476 |
| mmu-miR-1198-5p | -0,0289 | 7,6376 | -0,1983 | 0,8446 | 0,9188 | -6,0838 |
| mmu-miR-100-3p | 0,0579 | 1,2341 | 0,1975 | 0,8453 | 0,9188 | -5,4290 |
| mmu-miR-153-3p | -0,0429 | 7,9542 | -0,1952 | 0,8471 | 0,9190 | -6,0904 |
| mmu-miR-328-3p | 0,0259 | 12,2670 | 0,1930 | 0,8487 | 0,9190 | -6,2128 |
| mmu-miR-376a-5p | 0,0370 | 6,1223 | 0,1756 | 0,8622 | 0,9319 | -6,0086 |
| mmu-miR-190b-5p | -0,0574 | 1,1759 | -0,1709 | 0,8659 | 0,9337 | -5,4464 |
| mmu-miR-541-3p | -0,0326 | 2,7167 | -0,1693 | 0,8671 | 0,9337 | -5,6883 |
| mmu-miR-501-3p | 0,0176 | 6,8810 | 0,1660 | 0,8697 | 0,9347 | -6,0525 |
| mmu-miR-338-5p | -0,0262 | 9,1886 | -0,1624 | 0,8725 | 0,9360 | -6,1395 |
| mmu-miR-134-3p | -0,0314 | 2,2590 | -0,1599 | 0,8744 | 0,9363 | -5,6133 |
| mmu-miR-222-5p | 0,0281 | 5,1757 | 0,1576 | 0,8762 | 0,9365 | -5,9584 |
| mmu-miR-487b-5p | 0,0552 | 0,7470 | 0,1550 | 0,8782 | 0,9369 | -5,3622 |
| mmu-miR-483-3p | -0,0593 | 0,6226 | -0,1508 | 0,8816 | 0,9387 | -5,3559 |
| mmu-miR-666-5p | 0,0258 | 6,0961 | 0,1437 | 0,8871 | 0,9428 | -6,0198 |
| mmu-miR-344b-3p | -0,0210 | 7,8376 | -0,1390 | 0,8907 | 0,9445 | -6,0996 |
| mmu-miR-186-3p | 0,0529 | 0,7188 | 0,1360 | 0,8931 | 0,9445 | -5,3730 |
| mmu-miR-542-3p | -0,0282 | 3,6520 | -0,1349 | 0,8939 | 0,9445 | -5,8182 |
| mmu-miR-411-3p | -0,0169 | 8,9964 | -0,1332 | 0,8952 | 0,9445 | -6,1386 |
| mmu-miR-138-5p | 0,0212 | 10,1851 | 0,1301 | 0,8977 | 0,9454 | -6,1686 |
| mmu-miR-345-3p | -0,0182 | 5,0651 | -0,1133 | 0,9109 | 0,9563 | -5,9420 |
| mmu-miR-448-5p | -0,0600 | 0,6617 | -0,1125 | 0,9114 | 0,9563 | -5,3552 |
| mmu-miR-383-5p | -0,0121 | 10,4383 | -0,1031 | 0,9188 | 0,9623 | -6,1800 |
| mmu-miR-25-5p | 0,0231 | 1,8936 | 0,0992 | 0,9219 | 0,9638 | -5,5950 |
| mmu-miR-101a-3p | 0,0104 | 13,5098 | 0,0966 | 0,9239 | 0,9641 | -6,2583 |
| mmu-miR-1969 | 0,0172 | 2,2162 | 0,0795 | 0,9373 | 0,9742 | -5,6320 |
| mmu-miR-146b-5p | 0,0120 | 10,3507 | 0,0780 | 0,9385 | 0,9742 | -6,1796 |
| mmu-miR-206-3p | 0,0256 | 2,0678 | 0,0766 | 0,9396 | 0,9742 | -5,6031 |
| mmu-miR-1298-3p | -0,0218 | 5,8099 | -0,0758 | 0,9403 | 0,9742 | -6,0160 |
| mmu-miR-7079-3p | -0,0290 | 1,2377 | -0,0690 | 0,9456 | 0,9773 | -5,4484 |
| mmu-miR-6538 | 0,0777 | -0,8282 | 0,0677 | 0,9467 | 0,9773 | -5,2113 |
| mmu-miR-181b-1-3p | -0,0128 | 2,6374 | -0,0599 | 0,9528 | 0,9818 | -5,6673 |
| mmu-miR-582-5p | -0,0141 | 4,7020 | -0,0549 | 0,9567 | 0,9841 | -5,9125 |
| mmu-miR-200a-5p | -0,0184 | 3,9475 | -0,0390 | 0,9692 | 0,9923 | -5,8618 |
| mmu-miR-190a-5p | 0,0107 | 4,3149 | 0,0390 | 0,9693 | 0,9923 | -5,8802 |
| mmu-miR-3074-5p | 0,0139 | 1,6874 | 0,0380 | 0,9700 | 0,9923 | -5,5295 |
| mmu-miR-325-3p | -0,0065 | 3,1091 | -0,0348 | 0,9725 | 0,9923 | -5,7479 |
| mmu-miR-671-5p | -0,0075 | 4,3708 | -0,0312 | 0,9754 | 0,9923 | -5,8910 |
| mmu-miR-8111 | 0,0090 | 1,3540 | 0,0291 | 0,9770 | 0,9923 | -5,5265 |
| mmu-miR-3077-3p | -0,0075 | 1,8149 | -0,0291 | 0,9770 | 0,9923 | -5,5698 |
| mmu-miR-377-5p | -0,0037 | 3,7105 | -0,0225 | 0,9822 | 0,9923 | -5,8313 |
| mmu-miR-669a-3p | -0,0059 | 1,8292 | -0,0224 | 0,9823 | 0,9923 | -5,5615 |
| mmu-miR-652-5p | -0,0087 | 0,2675 | -0,0170 | 0,9866 | 0,9923 | -5,2765 |
| mmu-miR-6928-3p | 0,0042 | 2,7956 | 0,0170 | 0,9866 | 0,9923 | -5,7293 |
| mmu-miR-423-5p | -0,0019 | 6,8818 | -0,0159 | 0,9874 | 0,9923 | -6,0667 |
| mmu-miR-3102-3p | 0,0025 | 5,6644 | 0,0123 | 0,9903 | 0,9923 | -6,0015 |
| mmu-miR-6945-3p | 0,0033 | 1,8781 | 0,0117 | 0,9908 | 0,9923 | -5,5930 |
| mmu-miR-1981-3p | -0,0017 | 4,5430 | -0,0101 | 0,9920 | 0,9923 | -5,9165 |
| mmu-miR-3970 | -0,0021 | 2,9521 | -0,0098 | 0,9923 | 0,9923 | -5,7434 |

**Supplementary figure legends:**

**Supplementary figure 1. Inflammation did not affect the microgliosis at 1 dpi in aged animals** Quantified as the immunoreactivity for Iba-1, only young mice with LPS-induced inflammation had elevated levels of Iba-1 compared to vehicle-treated and aged inflamed animals **(a)**. Representative pictures from the peri-ischemic area of young vehicle **(b)**, young LPS **(c)**, aged vehicle **(d)** and aged LPS **(e)** mice. Scale bar 100 µm. The data are shown as mean ± SD. VEH = Vehicle treatment, LPS = LPS treatment, * p < 0.05 **(a)** F(1,22) = 7.638, p = 0.0113, interaction effect of Two way ANOVA followed by Tukey’s multiple comparisons test, adjusted p = 0.0161, n = 5-8).

**Supplementary figure 2. LPS treatment increased neutrophil infiltration regardless of age** Neutrophil infiltration to the lesion area was increased in both young and aged mice with peripheral inflammation compared to the animals without inflammation at 1 dpi **(a)**. Figures b-e show representative neutrophil staining in the lesion area of young vehicle **(b)**, young LPS **(c)**, aged vehicle **(d)** and aged LPS **(e)** mice. Scale bar 100 µm. The data are shown as mean ± SEM. VEH = Vehicle treatment, LPS = LPS treatment, * p < 0.05 **(a)** F(1,23) = 12.71, p = 0.0016, treatment effect of Two way ANOVA followed by Tukey’s multiple comparisons test, adjusted p = 0.0424, n = 6-8.

**Supplementary figure 3. Astrocytic activation was increased in all aged ischemic animals compared to young ones** Quantified as the immunoreactivity for GFAP at 1 dpi, peripheral inflammation did not affect to the astrocytosis **(a)**. Representative images from the peri-ischemic area of young vehicle **(b)**, young LPS **(c)**, aged vehicle **(d)** and aged LPS **(e)** mice. Scale bar 100 µm. The data are shown as mean ± SD. VEH = Vehicle treatment, LPS = LPS treatment, **** p < 0.0001 **(a)** F(1,23) = 27.08, p < 0.0001, age effect of Two way ANOVA, n = 6-8.

**Supplementary figures:**


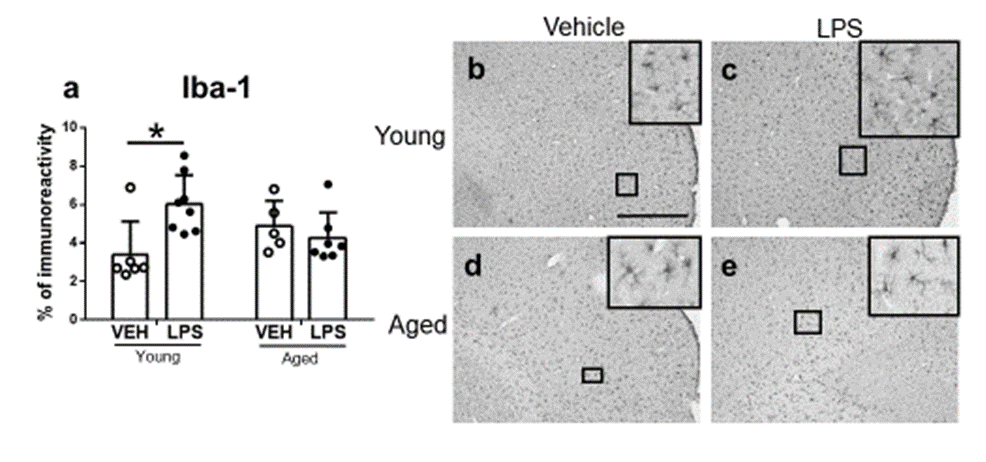
Supplementary figure 1.


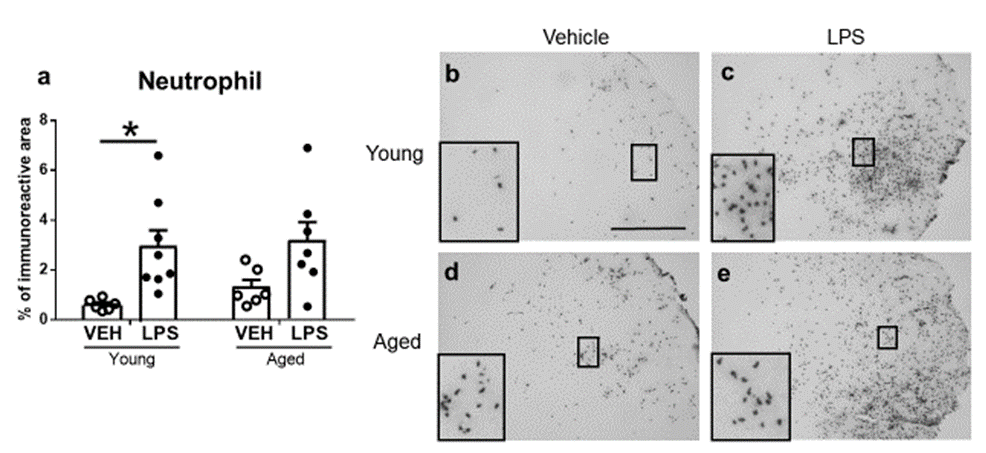
Supplementary figure 2.

Supplementary figure 3.


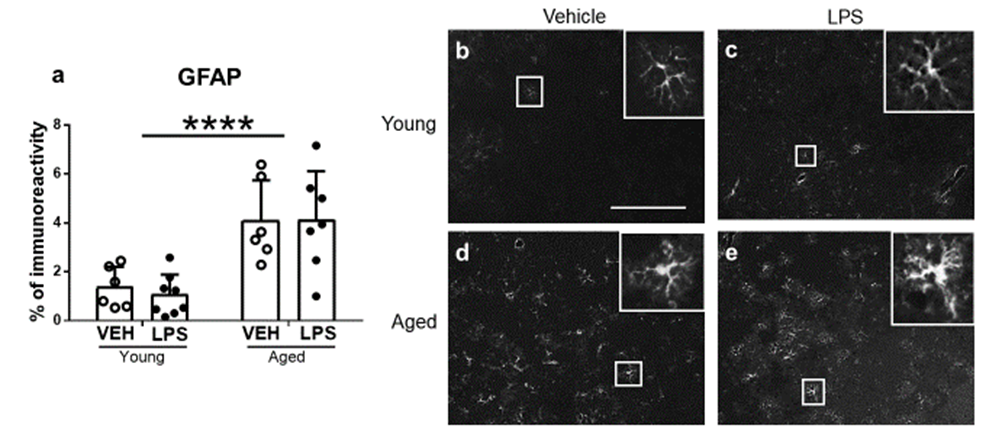

Supplement: Supplementary file 6 — Supplementary Material [file ACEL-20-e13287-s006.docx]
